# Metal-free synthesis of phosphinoylchroman-4-ones via a radical phosphinoylation–cyclization cascade mediated by K_2_S_2_O_8_

**DOI:** 10.3762/bjoc.16.164

**Published:** 2020-08-12

**Authors:** Qiang Liu, Weibang Lu, Guanqun Xie, Xiaoxia Wang

**Affiliations:** 1 Dongguan University of Technology, Dongguan 523808, P. R. China; 2 Department of Applied Chemistry, School of Science, Xi’an Jiaotong University, Xi’an 710049, P. R. China

**Keywords:** chroman-4-ones, diphenylphosphine oxide, metal-free, potassium persulfate, radical cyclization

## Abstract

A variety of chroman-4-ones bearing phosphine oxide motifs were conveniently synthesized from readily available diphenylphosphine oxides and alkenyl aldehydes via a metal-free tandem phosphinoylation/cyclization protocol. The reaction utilizes K_2_S_2_O_8_ as oxidant and proceeds in DMSO/H_2_O at environmentally benign conditions with a broad substrate scope and afforded the title compounds in moderate yields.

## Introduction

**Figure 1:**
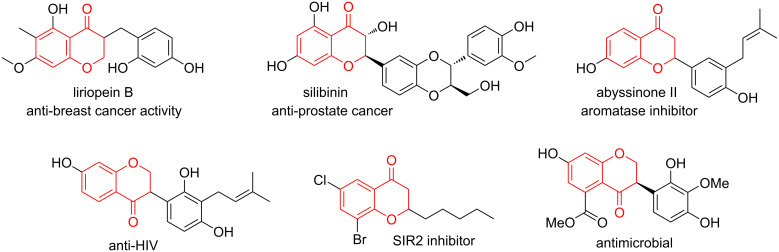
Biologically active compounds featuring the chroman-4-one framework.

The chroman-4-one framework is a privileged structural motif found in numerous natural products and pharmaceuticals with extraordinary biological and pharmaceutical activities such as anticancer activities and anti-HIV activity among others (Figure 1) [1–3]. Therefore, the preparation of functionalized chroman-4-one derivatives has attracted great attention of experts and scientists in the field of organic synthesis and pharmaceutical sciences over the last few years [1,4–5]. In general, chroman-4-one derivatives could be obtained via a polarity reversal strategy enabled by the N-heterocyclic carbene (NHC)-catalyzed intramolecular Stetter reaction [6–8]. Besides, chroman-4-one derivatives were also constructed via intramolecular oxa-Michael additions of 2’-hydroxychalcones [9–10], or through condensation cyclization reactions of *o*-hydroxyacetophenones with ketones/aldehydes [11–12], in addition to other alternative transformations [13–14]. Moreover, radical cascade cyclizations of *o*-allyloxybenzaldehydes by employing appropriate radical precursors through visible-light promoted systems [15–16], transition-metal-catalyzed systems [17–18], or transition-metal-free systems [19–20], have emerged as a powerful strategy for the synthesis of diversely functionalized chroman-4-one derivatives.

**Scheme 1:**
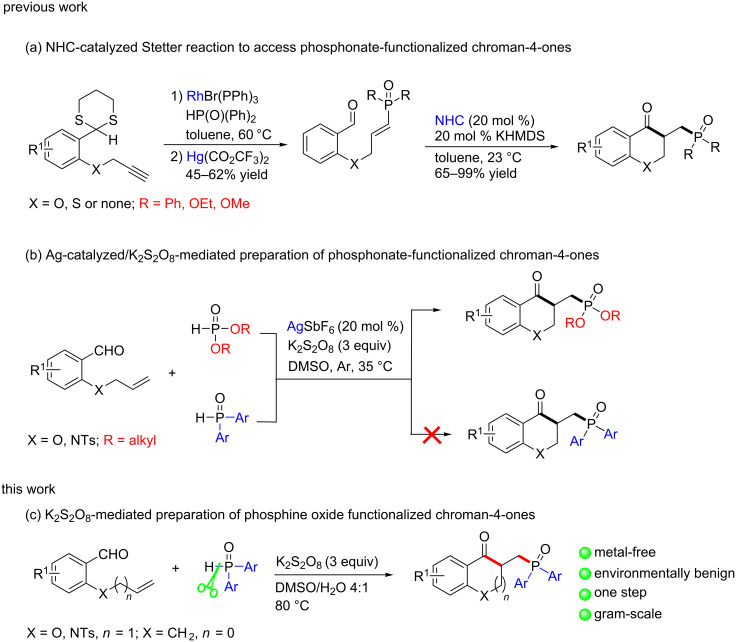
Methods to produce phosphonate-substituted chroman-4-ones.

Organophosphorus compounds are well-known for their medicinal, biological, or specific material-related properties and have found wide applications in pharmaceutical chemistry, biochemistry, and materials science [21–26]. They represent also excellent ligands for many metals and have been used in catalytic systems for a huge number of organic reactions [21–26]. Due to the importance of the chroman-4-one scaffold on one hand and that of organophosphorus compounds on the other, the development of concise and efficient approaches for the synthesis of chroman-4-one derivatives decorated with phosphorus functionalities [27–28], thus combining the characteristics of both components in one molecule may find useful applications. However, there are only few ways to prepare such compounds. For example, in 2008 Rovis et al. [27] reported an intramolecular Stetter reaction of alkenyl aldehydes to synthesize a series of phosphine oxide and phosphonate-functionalized chroman-4-ones. Unfortunately, the preparation of the substrates involved a Rh-catalyzed hydrophosphinylation of a protected functional alkyne, and the subsequent deprotection with Hg(O_2_CCF_3_)_2_, which is not environmentally benign (Scheme 1a). Besides, in 2016 Li’s group [28] reported a silver-catalyzed straightforward approach for the synthesis of phosphonate-functionalized chroman-4-ones via a phosphoryl radical-initiated cascade cyclization of 2-(allyloxy)arylaldehydes using K_2_S_2_O_8_ as an oxidant, however, diphenylphosphine oxide (DPPO) was not suitable for the transformation (Scheme 1b). So the development of metal-free and greener methods to approach chroman-4-ones bearing a phosphine oxide moiety is still highly desirable. Herein, we present a transition-metal-free radical cascade cyclization to access the desired chroman-4-one derivatives in one pot under environmentally benign conditions (Scheme 1c).

## Results and Discussion

**Figure 2:**
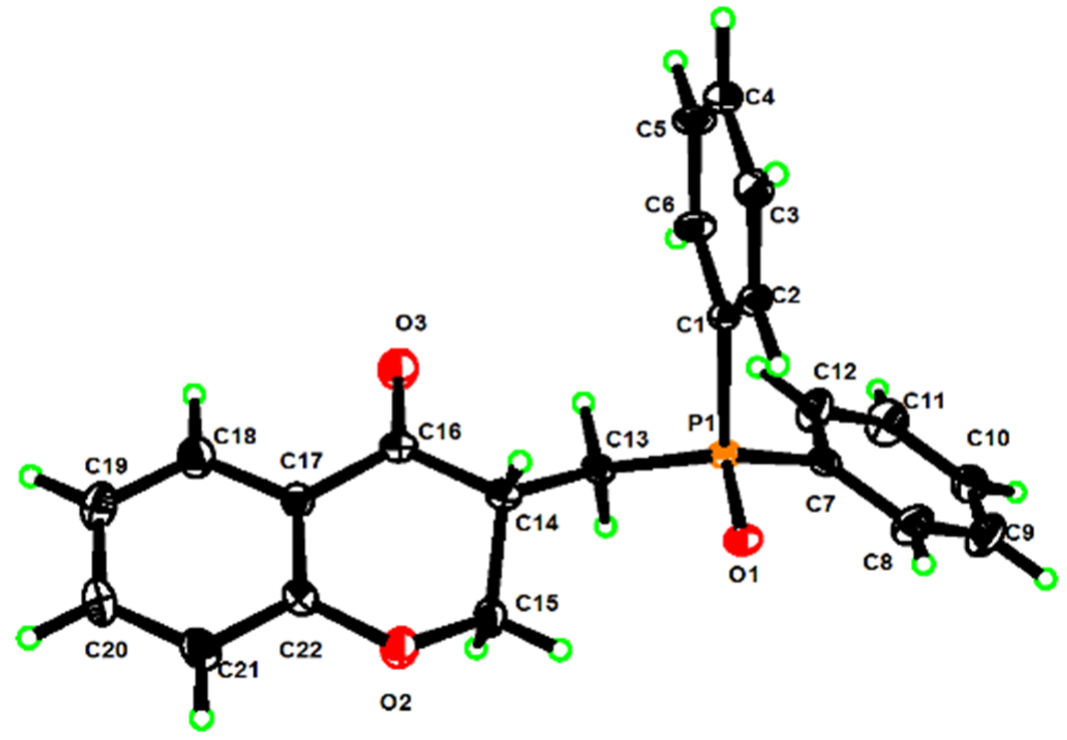
X-ray structure of compound **3aa** (CCDC 2002878).

Motivated by the desire to develop a metal-free and environmentally benign protocol for the construction of phosphine oxide-functionalized chroman-4-ones, we focused on the cascade cyclization employing 2-(allyloxy)benzaldehyde (**1a**) and diphenylphosphine oxide (DPPO, **2a**) as the model substrates with K_2_S_2_O_8_ as the oxidant, which is a cheap, readily available, and versatile oxidant. On the basis of literature reports [29–30] and our continuing interest in green chemistry [31–32], we set the temperature at 70 °C based on the fact that K_2_S_2_O_8_ thermally decomposes forming sulfate radicals (SO_4_^·−^) [29–30], which may react with the substrates to furnish such a cascade cyclization. To our delight, the anticipated product **3aa** was obtained in 42% yield in DMSO/H_2_O (4:1) as reaction medium in one pot (Table 1, entry 1). The structure of **3aa** was unambiguously confirmed by X-ray diffraction analysis of a single crystal (Figure 2) and by NMR spectroscopy (see Supporting Information File 1) [33]. The increase of the amount of K_2_S_2_O_8_ to 3 equiv resulted in the improvement of the yield of **3aa** to 52% (Table 1, entry 2). However, adjusting the amount of the oxidant K_2_S_2_O_8_ to 4 equiv (Table 1, entry 3) did not further improve the yield. By further screening of a few solvents, such as MeCN/H_2_O, DMF/H_2_O, DMA/H_2_O, dioxane/H_2_O, THF/H_2_O, EtOH/H_2_O, DCE/H_2_O, and NMP/H_2_O, it turned out that the highest yield was achieved in the DMSO/H_2_O (4:1) system (Table 1, entries 4–13). It is notable that product **3aa** was not observed at room temperature or in the absence of K_2_S_2_O_8_, indicating that the reaction was mainly mediated by K_2_S_2_O_8_ (Table 1, entries 14 and 15). Increasing the reaction temperature to 80 °C afforded better product yields as compared with the reactions performed at either 70 °C or 90 °C (Table 1, entries 2, 16, and 17). Then, various oxidants such as (NH_4_)_2_S_2_O_8_, Na_2_S_2_O_8_, TBHP (*tert*-butyl hydroperoxide), DTBP (di-*tert*-butyl peroxide), and dioxygen were tested and the results showed that K_2_S_2_O_8_ exhibited the best efficiency (Table 1, entries 18–22).

**Table 1:** Optimization of the reaction conditions.^a^

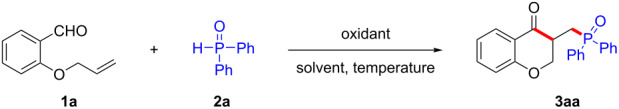				

Entry	Oxidant	Solvent	Temp. (°C)	Yield (%)^b^

1	K_2_S_2_O_8_ (2.0 equiv)	DMSO/H_2_O (4:1 v/v)	70	42
2	K_2_S_2_O_8_ (3.0 equiv)	DMSO/H_2_O (4:1v/v)	70	52
3	K_2_S_2_O_8_ (4.0 equiv)	DMSO/H_2_O (4:1v/v)	70	50
4	K_2_S_2_O_8_ (3.0 equiv)	MeCN/H_2_O (4:1 v/v)	70	32
5	K_2_S_2_O_8_ (3.0 equiv)	DMF/H_2_O (4:1 v/v)	70	24
6	K_2_S_2_O_8_ (3.0 equiv)	DMA/H_2_O (4:1 v/v)	70	21
7	K_2_S_2_O_8_ (3.0 equiv)	dioxane/H_2_O (4:1 v/v)	70	trace
8	K_2_S_2_O_8_ (3.0 equiv)	THF/H_2_O (4:1 v/v)	70	trace
9	K_2_S_2_O_8_ (3.0 equiv)	EtOH/H_2_O (4:1 v/v)	70	trace
10	K_2_S_2_O_8_ (3.0 equiv)	DCE/H_2_O (4:1 v/v)	70	trace
11	K_2_S_2_O_8_ (3.0 equiv)	NMP/H_2_O (4:1 v/v)	70	18
12	K_2_S_2_O_8_ (3.0 equiv)	DMSO/H_2_O (1:1 v/v)	70	32
13	K_2_S_2_O_8_ (3.0 equiv)	DMSO/H_2_O (8:1 v/v)	70	44
14	–	DMSO/H_2_O (4:1 v/v)	70	0
15	K_2_S_2_O_8_ (3.0 equiv)	DMSO/H_2_O (4:1 v/v)	rt^c^	0
16	K_2_S_2_O_8_ (3.0 equiv)	DMSO/H_2_O (4:1v/v)	80	58
17	K_2_S_2_O_8_ (3.0 equiv)	DMSO/H_2_O (4:1v/v)	90	54
18	(NH_4_)_2_S_2_O_8_ (3.0 equiv)	DMSO/H_2_O (4:1 v/v)	80	40
19	Na_2_S_2_O_8_ (3.0 equiv)	DMSO/H_2_O (4:1v/v)	80	50
20	DTBP (3.0 equiv)	DMSO/H_2_O (4:1 v/v)	80	0
21	TBHP (3.0 equiv)	DMSO/H_2_O (4:1 v/v)	80	0
22	O_2_	DMSO/H_2_O (4:1 v/v)	80	0

^a^Reaction conditions: **1a** (0.3 mmol, 1 equiv), **2a** (1.5 equiv), solvent (v/v, 5 mL), N_2_ atmosphere, 18 h. ^b^Isolated yields. ^c^Room temperature.

**Scheme 2:**
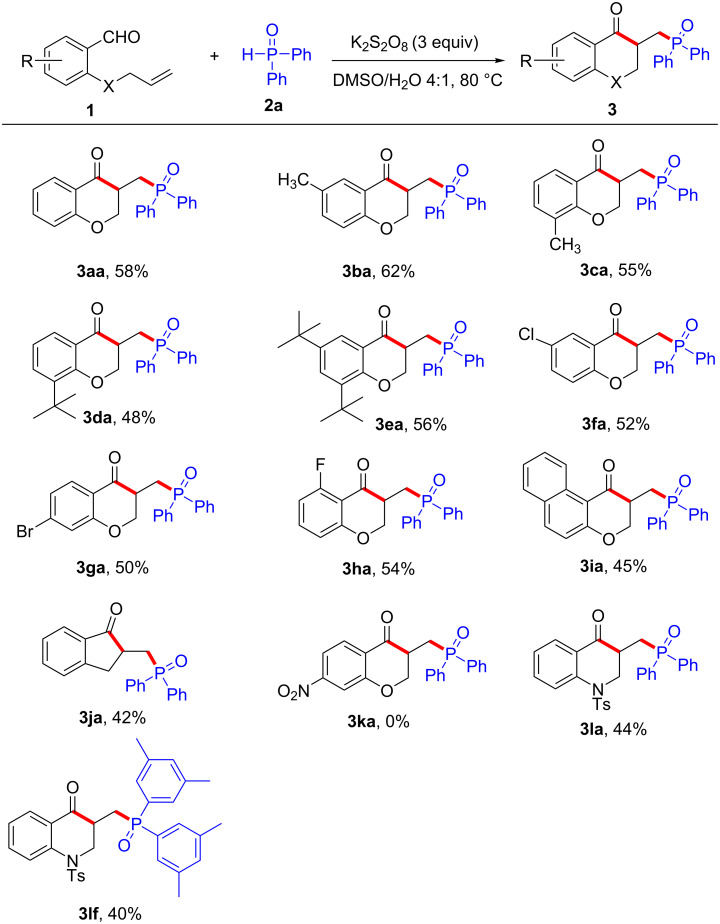
Scope of 2-(allyloxy)arylaldehydes. Reaction conditions: **1** (0.3 mmol, 1 equiv), **2a** (1.5 equiv) [**2f** for product **3lf**], DMSO/H_2_O (4:1 v/v, 5 mL), K_2_S_2_O_8_ (3.0 equiv), N_2_ atmosphere, 18 h. Yields are isolated yields.

With the optimal reaction conditions in hand (Table 1, entry 16), we next explored the scope and generality of this protocol using various 2-(allyloxy)arylaldehydes **1** for the reaction with **2a**. As shown in Scheme 2, substrates **1** with a range of functional groups, such as electron-donating groups Me- (**1b** and **1c**), *t*-Bu- (**1d** and **1e**), and electron-withdrawing groups Cl- (**1f**), Br- (**1g**), and F- (**1h**) were well tolerated in this transformation, providing the desired products **3aa**–**ha** in 48–62% yields. Furthermore, the transformation also proceeded with naphthyl substrate **1i** giving the desired product **3ia** in 45% yield. Notably, when the substrate was 2-allylbenzaldehyde (**1j**), the protocol was also compatible affording the indanone derivative **3ja** with comparable yield. This outcome is of special interest, because indanone derivatives are also privileged structural motifs found in numerous natural products and pharmaceuticals with extraordinary biological and pharmaceutical activities [34–35]. However, no desired product (**3ka**) was obtained when there was a nitro group in the substrate (**1k**). Then, *N*-allyl-*N*-(2-formylphenyl)-4-methylbenzenesulfonamide (**1l**) was examined in the cascade cyclization and the desired products **3la**, **3lf** were obtained in 44% and 40% yield.

**Scheme 3:**
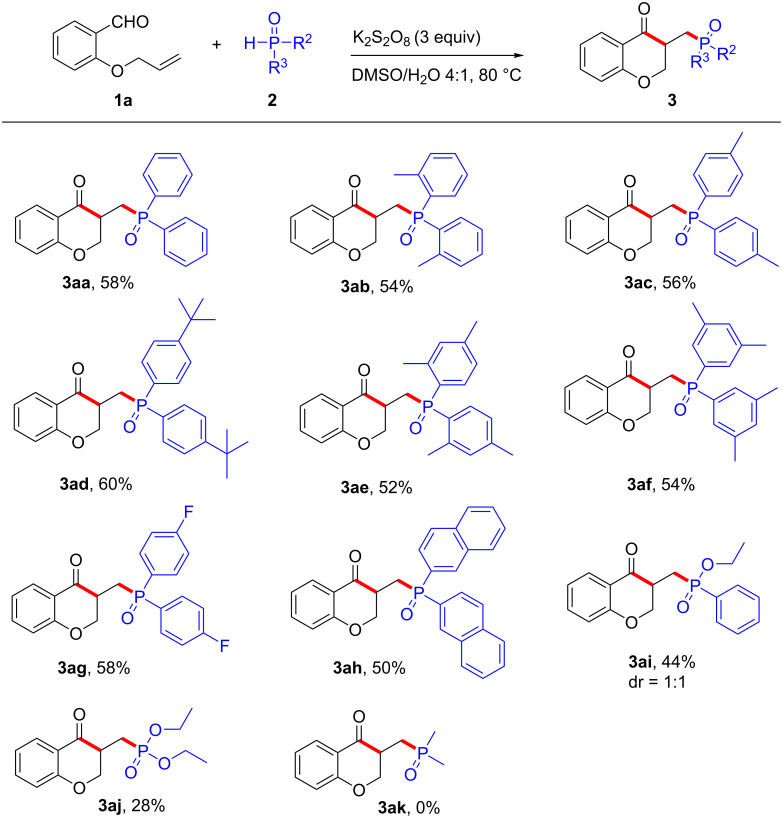
Scope of diphenylphosphine oxides. Reaction conditions: **1a** (0.3 mmol, 1 equiv), **2** (1.5 equiv), DMSO/H_2_O (4:1 v/v, 5 mL), K_2_S_2_O_8_ (3.0 equiv), N_2_ atmosphere, 18 h. Yields are isolated yields.

Next, to further demonstrate the generality of this strategy, the scope of different diphenylphosphine oxide **2** was examined as shown in Scheme 3. Simple diphenylphosphine oxides, such as 2-Me-DPPO (**2b**), 4-Me-DPPO (**2c**), and 4-*t*-Bu-DPPO (**2d**) furnished the corresponding products in good yields. Also multisubstituted diarylphosphine oxides **2e** and **2f** were well tolerated under these reaction conditions. Gratifyingly, diphenylphosphine oxides bearing fluoro-substituents (**2g**) reacted smoothly furnishing the anticipated product **3ag** in 58% yield. Furthermore, 1-naphthyl-DPPO (**2h**) was also suitable for this transformation, and afforded the expected product **3ah** in 50% yield. The reaction between diethyl phosphonate (**2j**) and **1a** proceeded less efficiently under the conditions and a low yield of **3aj** was obtained. Dimethylphosphine oxide (**2k**) did not participate in the reaction, likely due to its high oxidation potential and poor ability to undergo tautomerization [36].

**Scheme 4:**
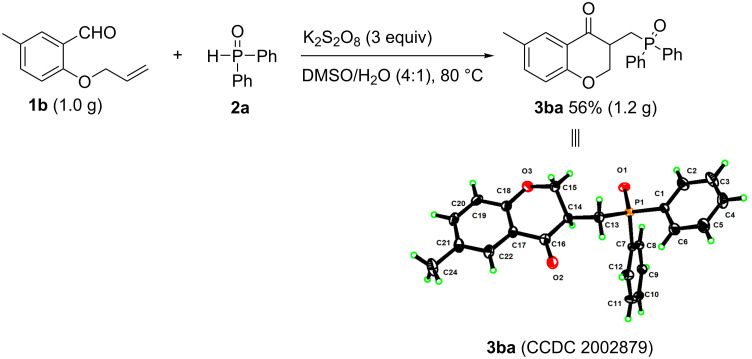
Gram-scale reaction.

To demonstrate the practicability of this methodology, a gram-scale experiment was next performed, employing **1b** and **2a** as substrates under the optimized conditions (Scheme 4). The reaction afforded the desired product **3ba** in a good yield of 56%, and the structure was also confirmed by X-ray diffraction (see Supporting Information File 1) [33].

**Scheme 5:**
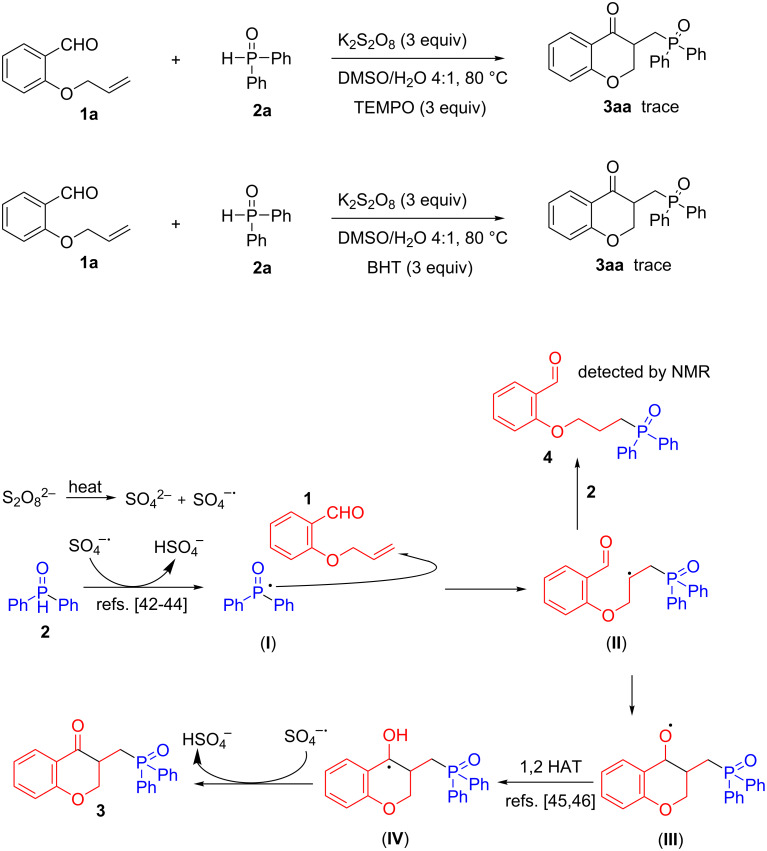
Control experiments and proposed mechanism.

To gain an insight into the reaction mechanism, we carried out some control experiments (Scheme 5). When the reaction was conducted in the presence of radical scavengers such as 2,2,6,6-tetramethyl-1-piperidinyloxyl (TEMPO) and butylated hydroxytoluene (BHT), the reactions were completely shut down, which indicated that the reaction proceeds through a radical pathway [37–41]. Also, we successfully separated a small amount of byproduct **4** which was identified by NMR spectroscopy. These experiments clearly support a phosphorus-centered radical reaction pathway. It has been reported that phosphorus-centered radicals could be generated from phosphine oxides in the presence of potassium persulfate [42–44]. Based on literature precedent [29–30,42–46] and preliminary mechanistic experiments, a plausible mechanism was proposed in Scheme 5 which was different from the predominant mechanism observed in the Ag-catalyzed radical cascade for the preparation of phosphonate-functionalized chroman-4-ones [28]. Initially, K_2_S_2_O_8_ thermally decomposes to form sulfate radical anions (SO_4_^•−^) [29–30], which react with diphenylphosphine oxide (DPPO, **2**) to give the phosphorus-centered radical **I** [42–44]. Then, the phosphorus centered radical **I** added to the C–C double bond of **1** to generate a new carbon-centered radical **II**, with sequential attack on the aldehyde group. The oxygen radical **III** thus formed undergoes a formal 1,2-H shift to generate the benzyl radical **IV** [45–46]. Finally, hydrogen abstraction by the sulfate radical anion (SO_4_^•−^) from the benzyl radical **IV** affords the final products **3** [45–46].

## Conclusion

In summary, an environmentally benign and practical radical cascade cyclization was developed to synthesize a series of phosphonate-functionalized chroman-4-ones from 2-(allyloxy)benzaldehydes and diphenylphosphine oxides. This protocol proceeds under metal-free conditions and uses cheap K_2_S_2_O_8_ as oxidant with easy handling and a broad substrate scope. The reaction proceeds through a radical phosphinoylation–cyclization via a tandem C–P and C–C-bond formation.

## Supporting Information

File 1 Experimental procedures, spectroscopic and X-ray data and copies of NMR spectra.
