# Supplementary material for: Metal-free synthesis of phosphinoylchroman-4-ones via a radical phosphinoylation–cyclization cascade mediated by K2S2O8
Source: Beilstein J Org Chem. 2020 Aug 12;16:1974–82. doi: 10.3762/bjoc.16.164 (PMC7431760; doi:10.3762/bjoc.16.164)
Supplement: File 1 — Experimental procedures, spectroscopic and X-ray data and copies of NMR spectra. [file Beilstein_J_Org_Chem-16-1974-s001.pdf]

BEILSTEIN JOURNAL OF ORGANIC CHEMISTRY

Supporting Information

for

Metal-free synthesis of phosphinoylchroman-4-ones via a radical phosphinoylation–cyclization cascade mediated by $K_2S_2O_8$

Qiang Liu, Weibang Lu, Guanqun Xie and Xiaoxia Wang

Beilstein J. Org. Chem. **2020,** *16,* 1974–1982. doi:10.3762/bjoc.16.164

Experimental procedures, spectroscopic and X-ray data and copies of NMR spectra

License and Terms: This is a supporting information file under the terms of the Creative Commons Attribution License (http://creativecommons.org/licenses/by/4.0). Please note that the reuse, redistribution and reproduction in particular requires that the authors and source are credited.
The license is subject to the *Beilstein Journal of Organic Chemistry* terms and conditions: (https://www.beilstein-journals.org/bjoc)

Table of contents

1.	General information	S2
2.	General procedures for the preparation of substrates	S2
3.	General procedures for the preparation of products.	S3
4.	Characterization of products	S4–S11
5.	References	S12
6.	^{1}H NMR, ^{13}C NMR, ^{19}F NMR and ^{31}P spectra for products	S13–S56
7.	X-ray crystallography data for **3aa** and **3ba**	S57–S65

1. General information

^{1}H NMR (600 MHz) and ^{13}C NMR (150 MHz) spectra were obtained in $CDCl_3$ or DMSO-d_6. HRMS (ESI) spectra were recorded on a 1200-6520 Q-TOF/Agilent mass spectrometer using electrospray ionization. The starting materials were purchased from Aldrich, Energy Chemical Chemicals used without further purification. Flash column chromatography was performed using 200–300 mesh silica gel.

2. General procedure for the preparation of substrates

General procedure for preparation of substrates 1 [1]

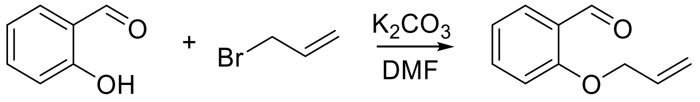

A solution of salicylaldehyde (5.0 g, 40.9 mmol) in dry DMF (30.0 mL) was treated with K_2CO_3 (6.2 g, 45.0 mmol) and allyl bromide (3.89 mL, 45.0 mmol). The mixture was stirred overnight at room temperature. Then, it was poured into saturated aqueous NH_4Cl and extracted 4 times with EtOAc. The organic phases were evaporated and then subjected to column chromatography (PE/EtOAc 50:1–10:1) to afford the starting materials **1** (6.0 g).

General procedure for the preparation of substrates 2 [2]

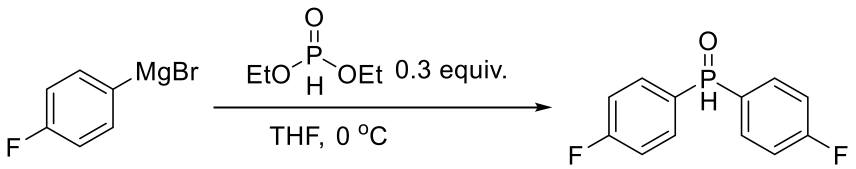

Diethyl phosphonate (0.456 g, 3.0 mmol) and anhydrous THF (20 ml) was added slowly to the Grignard reagent (purchased from Aldrich, 10.0 mmol, 1 M in THF) at 0 °C under N_2 atmosphere, then stirred at room temperature for 1.5 hours. After quenching with saturated aqueous NH_4Cl and extraction 4 times with EtOAc, the mixture was purified by silica gel column chromatography (dichloromethane/methanol 80:1–40:1) to afford the title compound as a colorless oil in 96% yield.

Procedure for the preparation of substrate 1j [3]

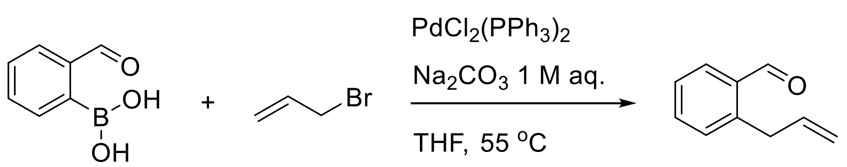

To a solution of the 2-formylphenylboronic acid and corresponding allyl halide (1.5 equiv) in THF (0.2 M) in a round-bottomed flask was added $PdCl_2(PPh_3)_2$ (2.5 mol %). The reaction mixture was heated to 50 °C, then aq Na_2CO_3 (1 M, 2 equiv) solution was added dropwise over a period of 1 h and the heating continued at reflux for 3–4 h. The reaction mixture was quenched with H_2O and extracted with DCM (three times). The combined organic phase was washed with brine, dried over $MgSO_4$, and concentrated in vacuum. The residue was purified by column chromatography on silica gel (EtOAc/PE 1:50) to afford the desired yellow product **1j**.

Procedure for the preparation of substrate **1l** according the literature [4].

3. General procedures for the preparation of products.

Under nitrogen, a reaction tube equipped with a magnetic stirring bar was charged with 2-(allyloxy)benzaldehyde (**1**, 0.30 mmol), and diphenylphosphine oxide (DPPO, **2**, 1.5 equiv), $K_2S_2O_8$ (3.0 equiv), and DMSO/H_2O 4:1 (5.0 mL), respectively. The mixture was allowed to react at 80 °C for 18 h. When the reaction was completed, the mixture was charged in 30 mL water and extracted with CH_2Cl_2 (15 mL, 3 times). The CH_2Cl_2 layers were combined and dried over Na_2SO_4, and further purified by column chromatography on silica gel (eluent, petroleum ether/ethyl acetate 1:1 or CH_2Cl_2/ MeOH 20:1) to afford the desired product **3**

Gram-scale reaction:

Under nitrogen, a reaction tube equipped with a magnetic stirring bar was charged with **1b** (1.0 g), **2a** (1.5 equiv), $K_2S_2O_8$ (3.0 equiv), and DMSO/H_2O 4:1 (50.0 mL), respectively. The mixture was allowed to react at 80 °C for 12 h. When the reaction was completed, the mixture was charged in 400 mL water and then extracted with CH_2Cl_2 (50 mL, 3 times). The CH_2Cl_2 layers were combined and dried over Na_2SO_4, and the solvent was removed to afford the crude product, which was further purified by column chromatography on silica gel (eluent, petroleum ether/ethyl acetate 1:1) to obtain the desired product **3ba**.

4. Characterization of products

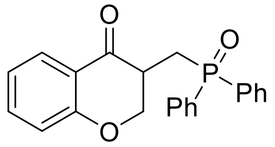

3-((Diphenylphosphoryl)methyl)chroman-4-one (**3aa**).[5]

White solid, 63 mg, 58 % yield; R_f = 0.28 (Petroleum ether/EtOAc = 1:1 v/v); ^{1}H NMR (600 MHz, DMSO-d6) δ 7.98 – 7.77 (m, 4H), 7.74 (dd, *J* = 7.8, 1.4 Hz, 1H), 7.56 (m, 7H), 7.17 – 6.87 (m, 2H), 4.65 (dd, *J* = 11.3, 5.2 Hz, 1H), 4.36 (t, *J* = 11.5 Hz, 1H), 3.13 (ddd, *J* = 15.6, 9.3, 2.4 Hz, 1H), 3.07 – 2.93 (m, 1H), 2.38 (ddd, *J* = 15.6, 12.1, 10.5 Hz, 1H). ^{13}C NMR (150 MHz, DMSO-D6) δ 192.17 (d, *J* = 12.2 Hz), 161.6, 136.7, 134.5 (d, *J* = 98.0 Hz), 133.1 (d, *J* = 97.9 Hz), 132.3, 131.0 (d, *J* = 9.3 Hz), 130.7 (d, *J* = 9.5 Hz), 129.3, 129.3, 129.2, 127.3, 121.9, 120.3, 118.2, 70.3, 40.8 (d, *J* = 2.5 Hz), 24.6 (d, *J* = 72.3 Hz). ^{31}P NMR (243 MHz, DMSO-d6) δ 29.5. HRMS (ESI-TOF) m/z calcd for $C_{22}H_{20}O_3P$, 363.1145, [M + H]+, found 363.1146.

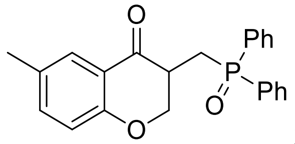

3-((Diphenylphosphoryl)methyl)-6-methylchroman-4-one (**3ba**).

White solid. 70 mg, 62 % yield; R_f = 0.35 (PE:EtOAc, 1:1); ^{1}H NMR (600 MHz, DMSO-d6) δ 7.96 – 7.72 (m, 4H), 7.62 – 7.47 (m, 7H), 7.36 (dd, *J* = 8.5, 2.3 Hz, 1H), 6.90 (d, *J* = 8.4 Hz, 1H), 4.61 (dd, *J* = 11.3, 5.1 Hz, 1H), 4.30 (t, *J* = 11.4 Hz, 1H), 3.11 (ddd, *J* = 15.6, 9.3, 2.5 Hz, 1H), 3.00 – 2.87 (m, 1H), 2.37 (ddd, *J* = 15.6, 12.1, 10.4 Hz, 1H), 2.24 (s, 3H). ^{13}C NMR (150 MHz, DMSO-d6) δ 192.2 (d, *J* = 12.1 Hz), 159.6, 137.6, 134.5 (d, *J* = 98.0 Hz), 133.1 (d, *J* = 97.6 Hz), 132.3 (d, *J* = 2.4 Hz), 131.0 (d, *J* = 9.2 Hz), 130.9, 130.7 (d, *J* = 9.4 Hz), 129.37, 129.29, 129.22, 126.7, 119.9, 118.0, 70.3, 40.8 (d, *J* = 2.6 Hz), 24.6 (d, *J* = 72.2 Hz), 20.3. ^{31}P NMR (243 MHz, DMSO-d6) δ 29.5. HRMS (ESI-TOF) m/z calcd for $C_{23}H_{22}O_3P$, 377.1301, [M + H]+, found 377.1302.

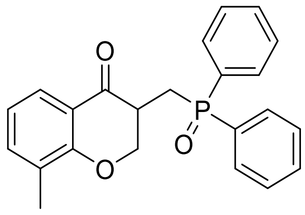

3-((Diphenylphosphoryl)methyl)-8-methylchroman-4-one (**3ca**).

White solid, 62 mg, 55% yield; R_f = 0.37 (Petroleum ether/EtOAc = 1:1 v/v); ^{1}H NMR (600 MHz, DMSO-D6) δ 7.85 (m, 4H), 7.56 (m, 7H), 7.42 (m, 1H), 6.95 (m, 1H), 4.68 (m, 1H), 4.35 (m, 1H), 3.11 (m, 1H), 2.98 (m, 1H), 2.39 (m, 1H), 2.14 (s, 3H). ^{13}C NMR (150 MHz, DMSO-D6) δ 192.4 (d, *J* = 11.9 Hz), 159.8, 137.2, 134.5 (d, *J* = 96.3 Hz), 133.2 (d, *J* = 98.2 Hz), 132.3, 131.0 (d, *J* = 9.1 Hz), 130.7 (d, *J* = 9.4 Hz), 129.3 (d, *J* = 10.8 Hz), 129.2 (d, *J* = 10.5 Hz), 127.1, 124.9, 121.3, 119.9, 70.3, 40.6, 24.6 (d, *J* = 72.5 Hz), 15.6. ^{31}P NMR (243 MHz, DMSO-D6) δ 29.5. HRMS (ESI-TOF) m/z calcd for $C_{23}H_{22}O_3P$, 377.1301, [M + H]+, found 377.1302.

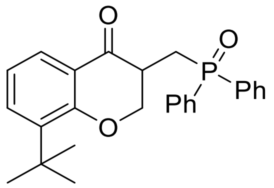

8-(*tert*-Butyl)-3-((diphenylphosphoryl)methyl)chroman-4-one (**3da**).
White solid, 60 mg, 48 % yield; R_f = 0.42 (Petroleum ether/EtOAc = 1:1 v/v); ^{1}H NMR (600 MHz, $CDCl_3$) δ 7.93 – 7.81 (m, 2H), 7.82 – 7.63 (m, 3H), 7.65 – 7.36 (m, 7H), 6.93 (t, *J* = 7.7 Hz, 1H), 5.07 (dd, *J* = 11.3, 5.4 Hz, 1H), 4.24 (t, *J* = 11.7 Hz, 1H), 3.34 (ddd, *J* = 15.8, 7.5, 1.7 Hz, 1H), 3.24 – 2.97 (m, 1H), 2.18 – 1.94 (m, 1H), 1.35 (s, 9H). ^{13}C NMR (150 MHz, $CDCl_3$) δ 193.0 (d, *J* = 12.5 Hz), 161.0, 139.1, 133.6 (d, *J* = 100.2 Hz), 133.1, 132.1, 132.0 (d, *J* = 2.6 Hz), 131.2 (d, *J* = 98.9 Hz), 131.0 (d, *J* = 9.2 Hz), 130.4 (d, *J* = 9.5 Hz), 129.0 (d, *J* = 11.7 Hz), 128.8 (d, *J* = 11.8 Hz), 125.5, 121.0, 120.8, 70.2, 40.6 (d, *J* = 2.4 Hz), 34.9, 29.5, 25.0 (d, *J* = 73.6 Hz). ^{31}P NMR (243 MHz, $CDCl_3$) δ 32.3. HRMS (ESI-TOF) m/z calcd for $C_{26}H_{28}O_3P$, 419.1771, [M + H]+, found 419.1772.

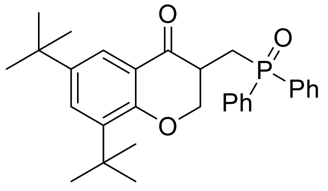

6,8-Di-*tert*-butyl-3-((diphenylphosphoryl)methyl)chroman-4-one (**3ea**).
Oil, 80 mg, 56 % yield; ^{1}H NMR (600 MHz, DMSO-D6) δ 7.85 (d, *J* = 4.7 Hz, 4H), 7.56 (m, 8H), 4.70 (dd, *J* = 10.9, 4.6 Hz, 1H), 4.33 (t, *J* = 11.1 Hz, 1H), 3.19 – 3.04 (m, 1H), 2.99 (d, *J* = 8.5 Hz, 1H), 2.45 – 2.32 (m, 1H), 1.31 (s, 9H), 1.23 (s, 9H). ^{13}C NMR (150 MHz, DMSO-D6) δ 192.9, 158.8, 143.2, 138.3, 134.6 (d, *J* = 97.4 Hz), 133.3 (d, *J* = 96.7 Hz), 132.3, 131.0 (d, *J* = 9.3 Hz), 130.7 (d, *J* = 9.6 Hz), 130.5, 129.3, 129.2, 129.2, 121.1, 120.4, 70.0, 40.7, 35.1, 34.5, 31.4, 29.9, 24.8 (d, *J* = 71.1 Hz). ^{31}P NMR (243 MHz, DMSO-D6) δ 29.5. HRMS (ESI-TOF) m/z calcd for $C_{30}H_{36}O_3P$, 475.2397, [M + H]+, found 475.2398.

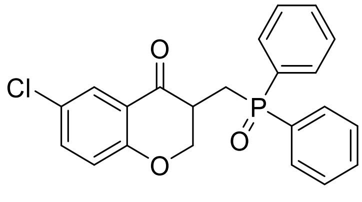

6-Chloro-3-((diphenylphosphoryl)methyl)chroman-4-one (**3fa**).[5]
White solid, 62 mg, 52 % yield; ^{1}H NMR (600 MHz, DMSO-D6) δ 7.95 – 7.73 (m, 4H), 7.73 – 7.42 (m, 8H), 7.05 (d, *J* = 8.9 Hz, 1H), 4.70 (dd, *J* = 11.4, 5.2 Hz, 1H), 4.40 (t, *J* = 11.5 Hz, 1H), 3.24 – 3.09 (m, 1H), 3.07 (m, 1H), 2.45 – 2.29 (m, 1H). ^{13}C NMR (150 MHz, DMSO -d6) δ 191.3 (d, *J* = 12.3 Hz), 160.2, 136.1, 134.4 (d, *J* = 98.3 Hz), 133.0 (d, *J* = 97.7 Hz), 132.3, 131.1 (d, *J* = 9.3 Hz), 130.7 (d, *J* = 9.5 Hz), 129.36, 129.29, 129.21, 126.1, 126.0, 121.3, 120.6, 70.5, 40.5 (d, *J* = 2.4 Hz), 24.6 (d, *J* = 72.2 Hz). ^{31}P NMR (243 MHz, DMSO-d6) δ 29.4. HRMS (ESI-TOF) m/z calcd for $C_{22}H_{19}O_3PCl$, 397.0755, [M + H]+, found 397.0754.

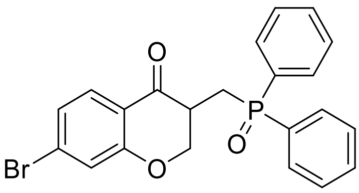

7-Bromo-3-((diphenylphosphoryl)methyl)chroman-4-one (**3ga**).

White solid, 66 mg, 50 % yield; ^{1}H NMR (600 MHz, DMSO-D6) δ 7.92 – 7.81 (m, 4H), 7.65 (d, *J* = 8.4 Hz, 1H), 7.61 – 7.46 (m, 6H), 7.31 (d, *J* = 1.8 Hz, 1H), 7.25 (dd, *J* = 8.4, 1.8 Hz, 1H), 4.67 (dd, *J* = 11.4, 5.2 Hz, 1H), 4.40 (t, *J* = 11.4 Hz, 1H), 3.11 (ddd, *J* = 15.5, 9.3, 2.7 Hz, 1H), 3.08 – 2.98 (m, 1H), 2.38 (ddd, *J* = 15.6, 12.2, 10.2 Hz, 1H). ^{13}C NMR (151 MHz, DMSO-D6) δ 191.5 (d, *J* = 12.1 Hz), 161.9, 134.5 (d, *J* = 98.3 Hz), 133.1 (d, *J* = 97.7 Hz), 132.3 (d, *J* = 2.3 Hz), 131.4 (d, *J* = 10.3 Hz), 131.0 (d, *J* = 9.3 Hz), 130.7 (d, *J* = 9.5 Hz), 129.8, 129.5 (d, *J* = 12.7 Hz), 129.3 (s), 129.3 (d, *J* = 1.2 Hz), 129.2, 129.0, 125.2, 121.0, 119.6, 70.7, 40.6, 24.6 (d, *J* = 72.0 Hz). ^{31}P NMR (243 MHz, DMSO-D6) δ 29.3. HRMS (ESI-TOF) m/z calcd for $C_{22}H_{19}O_3PBr$, 441.0250, [M + H]+, found 441.0245.

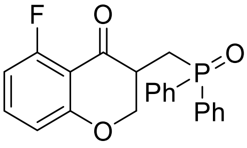

3-((Diphenylphosphoryl)methyl)-5-fluorochroman-4-one (**3ha**).

White solid, 61 mg, 54 % yield; ^{1}H NMR (600 MHz, DMSO-D6) δ 7.84 (dt, *J* = 11.6, 6.9 Hz, 4H), 7.70 – 7.31 (m, 7H), 6.83 (t, *J* = 9.8 Hz, 2H), 4.67 (dd, *J* = 11.2, 5.0 Hz, 1H), 4.40 (t, *J* = 11.3 Hz, 1H), 3.11 (dd, *J* = 13.2, 9.4 Hz, 1H), 3.00 (m, 1H), 2.35 (m, 1H). ^{13}C NMR (150 MHz, DMSO-D6) δ 189.7 (d, *J* = 12.5 Hz), 162.3 (d, *J* = 2.7 Hz), 161.3 (d, *J* = 262.9 Hz), 136.9 (d, *J* = 11.8 Hz), 134.5 (d, *J* = 98.4 Hz), 133.0 (d, *J* = 97.7 Hz), 132.3, 131.0 (d, *J* = 9.3 Hz), 130.7 (d, *J* = 9.5 Hz), 129.3 (d, *J* = 11.0 Hz), 129.2 (d, *J* = 11.1 Hz), 114.2, 110.4 (d, *J* = 8.9 Hz), 109.0 (d, *J* = 20.7 Hz), 70.1, 41.3, 24.5 (d, *J* = 72.1 Hz). ^{19}F NMR (565 MHz, DMSO-D6) δ -111.5. ^{31}P NMR (243 MHz, DMSO-D6) δ 29.5. HRMS (ESI-TOF) m/z calcd for $C_{22}H_{19}O_3PF$, 381.1050, [M + H]+, found 381.1052.

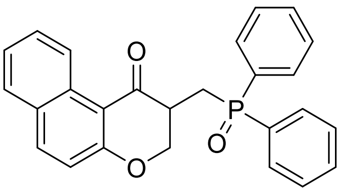

2-((Diphenylphosphoryl)methyl)-2,3-dihydro-1*H*-benzo[*f*]chromen-1-one (**3ia**).

Oil, 56 mg, 45 % yield; ^{1}H NMR (600 MHz, DMSO-D6) δ 9.29 (d, *J* = 8.6 Hz, 1H), 8.12 (d, *J* = 9.1 Hz, 1H), 8.00 – 7.80 (m, 5H), 7.71 – 7.62 (m, 1H), 7.62 – 7.51 (m, 6H), 7.47 (dd, *J* = 11.0, 3.9 Hz, 1H), 7.18 (d, *J* = 9.0 Hz, 1H), 4.77 (dd, *J* = 11.3, 5.1 Hz, 1H), 4.52 (t, *J* = 11.2 Hz, 1H), 3.19 (ddd, *J* = 15.5, 9.3, 2.7 Hz, 1H), 3.13 – 3.01 (m, 1H), 2.46 (ddd, *J* = 15.5, 12.2, 10.5 Hz, 1H). ^{13}C NMR (150 MHz, DMSO-D6) δ 193.2 (d, *J* = 12.1 Hz), 163.6, 138.1, 134.6 (d, *J* = 98.0 Hz), 133.2 (d, *J* = 97.4 Hz), 132.33, 132.32, 131.4, 131.1 (d, *J* = 9.3 Hz), 130.7 (d, *J* = 9.4 Hz), 129.9, 129.37, 129.30, 129.3 (d, *J* = 2.5 Hz), 129.2 (d, *J* = 2.3 Hz), 125.3, 125.2, 119.2, 111.4, 70.2, 41.2, 25.0 (d, *J* = 71.9 Hz). ^{31}P NMR (243 MHz, DMSO-D6) δ 29.6. HRMS (ESI-TOF) m/z calcd for $C_{26}H_{22}O_3P$, 413.1301, [M + H]+, found 413.1300.

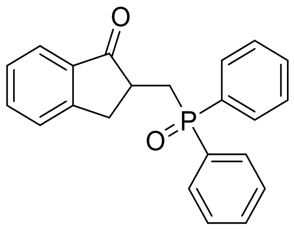

2-((Diphenylphosphoryl)methyl)-2,3-dihydro-1*H*-inden-1-one (**3ja**).[5] Oil, 44 mg, 42 % yield; ^{1}H NMR (600 MHz, DMSO-D6) δ 7.91 – 7.82 (m, 4H), 7.65 (t, *J* = 8.5 Hz, 2H), 7.61 – 7.52 (m, 6H), 7.49 (d, *J* = 7.5 Hz, 1H), 7.41 (t, *J* = 7.4 Hz, 1H), 3.11 (dd, *J* = 17.2, 7.9 Hz, 1H), 3.03 – 2.92 (m, 1H), 2.84 (ddd, *J* = 16.4, 13.6, 6.2 Hz, 2H), 2.63 (dt, *J* = 15.0, 10.9 Hz, 1H). ^{13}C NMR (150 MHz, DMSO-D6) δ 206.9 (d, *J* = 14.4 Hz), 154.0, 135.6, 135.5, 134.51 (d, *J* = 96.7 Hz), 134.00 (d, *J* = 96.7 Hz), 132.2, 131.0 (d, *J* = 9.5 Hz), 130.9 (d, *J* = 9.3 Hz), 129.3 (d, *J* = 11.4 Hz), 129.1 (d, *J* = 11.4 Hz), 127.9, 127.2, 123.7, 41.6 (d, *J* = 3.9 Hz), 33.7, 30.6 (d, *J* = 72.8 Hz). ^{31}P NMR (243 MHz, DMSO-D6) δ 29.2. HRMS (ESI-TOF) m/z calcd for $C_{22}H_{20}O_2P$, 347.1195, [M + H]+, found 347.1202.

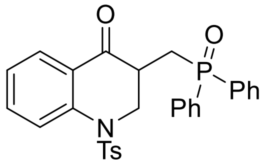

3-((Diphenylphosphoryl)methyl)-1-tosyl-2,3-dihydroquinolin-4(1*H*)-one (**3la**). White solid. 67 mg. M.p. = 122-123 ℃. Rf (EtOAc/PE 1:1) = 0.44. ^{1}H NMR (600 MHz, DMSO-d_6) δ 7.92 – 7.77 (m, 6H), 7.73 – 7.64 (m, 2H), 7.64 – 7.56 (m, 3H), 7.53 (t, *J* = 6.0 Hz, 2H), 7.38 – 7.28 (m, 3H), 7.04 (d, *J* = 8.1 Hz, 2H), 4.97 (dd, *J* = 14.3, 5.2 Hz, 1H), 3.91 (t, *J* = 13.8 Hz, 1H), 3.18 (dd, *J* = 14.9, 7.8 Hz, 1H), 2.56 – 2.40 (m, 1H), 2.31 – 2.23 (m, 1H), 2.20 (s, 3H). ^{13}C NMR (150 MHz, DMSO-d_6) δ 193.22 (d, *J* = 12.7 Hz), 144.7, 142.0, 136.2, 135.4, 134.6 (d, *J* = 98.3 Hz), 132.8 (d, *J* = 97.4 Hz), 132.4, 132.2, 131.1 (d, *J* = 9.1 Hz), 130.6 (d, *J* = 9.4 Hz), 130.4, 129.4 (d, *J* = 11.3 Hz), 129.3, 129.25 (d, *J* = 11.5 Hz), 128.1, 127.9, 126.8, 126.1, 124.6, 124.2, 50.6, 40.1, 26.2 (d, *J* = 72.9 Hz), 21.4. ^{31}P NMR (243 MHz, DMSO-d_6) δ 29.7. HRMS (ESI-TOF) m/z calcd for $C_{29}H_{27}NO_4PS$, 515.1320, [M + H]+, found 515.1322.

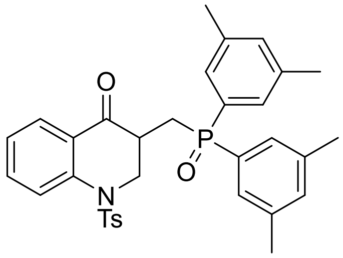

3-((Bis(3,5-dimethylphenyl)phosphoryl)methyl)-1-tosyl-2,3-dihydroquinolin-4(1*H*)-one (**3lf**). White solid. 68 mg. M.P. = 192-193℃ Rf (EtOAc/PE 1:1) = 0.64. 1H NMR (600 MHz, DMSO-d6) δ 7.89 – 7.78 (m, 2H), 7.76 – 7.60 (m, 1H), 7.43 (dd, J = 19.1, 11.5 Hz, 4H), 7.34 (dd, J = 7.3, 5.3 Hz, 3H), 7.28 (s, 1H), 7.20 (s, 1H), 7.04 (d, J = 8.2 Hz, 2H), 4.92 (dd, J = 14.3, 5.2 Hz, 1H), 3.85 (t, J = 13.8 Hz, 1H), 3.08 (dd, J = 13.9, 8.6 Hz, 1H), 2.50 – 2.41 (m, 1H), 2.36 (s, 6H), 2.32 (s, 6H), 2.23 (s, 3H), 2.18 – 2.02 (m, 1H). ^{13}C NMR (150 MHz, DMSO-d6) δ 193.3 (d, *J* = 12.7 Hz), 144.6, 142.0, 138.6 (d, *J* = 12.2 Hz), 138.5 (d, *J* = 12.0 Hz),

135.8 (d, *J* = 114.7 Hz), 134.6 (d, *J* = 97.4 Hz), 133.7, 133.5, 133.3, 132.7, 130.3, 128.5 (d, *J* = 8.9 Hz), 128.2, 128.1 (d, *J* = 9.5 Hz), 126.9 126.1, 124.6, 124.0, 50.5, 26.2 (d, *J* = 72.8 Hz), 21.38, 21.34, 21.31. ^{31}P NMR (243 MHz, DMSO-d6) δ 29.4. HRMS (ESI-TOF) m/z calcd for HRMS (ESI-TOF) m/z calcd for $C_{33}H_{35}NO_4PS$, 572.2019, [M + H]+, found 572.2020.

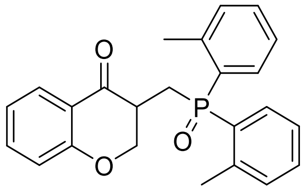

3-((Di-*o*-tolylphosphoryl)methyl)chroman-4-one (**3ab**).
White solid, 63 mg, 54% yield; ^{1}NMR (600 MHz, DMSO-D6) δ 7.84 (dd, *J* = 12.0, 7.6 Hz, 1H), 7.76 (m, 2H), 7.57 (m, 1H), 7.50 (dd, *J* = 13.8, 7.4 Hz, 2H), 7.39 (q, *J* = 7.6 Hz, 2H), 7.32 – 7.23 (m, 2H), 7.13 – 7.05 (m, 1H), 7.03 (d, *J* = 8.3 Hz, 1H), 4.66 (dd, *J* = 11.3, 5.3 Hz, 1H), 4.39 (t, *J* = 11.5 Hz, 1H), 3.16 (ddd, *J* = 15.6, 8.9, 1.9 Hz, 1H), 3.09 – 2.87 (m, 1H), 2.42 (m, 1H), 2.24 (s, 3H), 2.23 (s, 3H). ^{13}C NMR (150 MHz, DMSO-D6) δ 192.3 (d, *J* = 12.0 Hz), 161.7, 141.2 (d, *J* = 2.8 Hz), 141.1 (d, *J* = 3.5 Hz), 136.7, 132.5 (d, *J* = 4.4 Hz), 132.5 (d, *J* = 3.2 Hz), 132.4 (d, *J* = 96.1 Hz), 132.3 (d, *J* = 10.2 Hz), 132.2 (d, *J* = 10.1 Hz), 131.8, 131.7, 131.0 (d, *J* = 94.3 Hz), 127.3, 126.4 (d, *J* = 2.5 Hz), 126.4 (d, *J* = 2.7 Hz), 121.9, 120.3, 118.3, 70.4, 40.8, 23.7 (d, *J* = 72.7 Hz), 21.0 (d, *J* = 4.0 Hz), 20.9(d, *J* = 4.3 Hz). ^{31}P NMR (243 MHz, DMSO-D6) δ 32.1. HRMS (ESI-TOF) m/z calcd for $C_{24}H_{24}O_3P$, 391.1458, [M + H]+, found 391.1460.

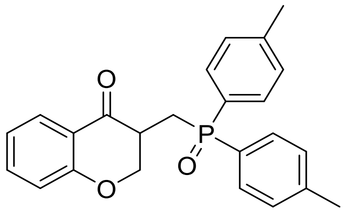

3-((Di-*p*-tolylphosphoryl)methyl)chroman-4-one (**3ac**).

White solid, 66 mg, 56 % yield; ^{1}H NMR (600 MHz, DMSO-d6) δ 7.86 – 7.62 (m, 5H), 7.55 (t, *J* = 7.4 Hz, 1H), 7.34 (d, *J* = 7.4 Hz, 4H), 7.06 (t, *J* = 7.4 Hz, 1H), 7.01 (d, *J* = 8.3 Hz, 1H), 4.65 (dd, *J* = 11.3, 5.1 Hz, 1H), 4.35 (t, *J* = 11.5 Hz, 1H), 3.12 – 3.03 (m, 1H), 2.97 (d, *J* = 11.3 Hz, 1H), 2.41 – 2.05 (m, 7H). ^{13}C NMR (150 MHz, DMSO-D6) δ 192.2 (d, *J* = 12.3 Hz), 161.6, 142.3, 136.7, 131.4 (d, *J* = 101.7 Hz), 131.0 (d, *J* = 9.6 Hz), 130.7 (d, *J* = 9.7 Hz), 130.0 (d, *J* = 97.4 Hz), 129.9 (d, *J* = 11.8 Hz), 129.8 (d, *J* = 11.8 Hz), 127.3, 121.9, 120.3, 118.2, 70.3, 40.8, 24.7 (d, *J* = 72.7 Hz), 21.5. ^{31}P NMR (243 MHz, DMSO-D6) δ 29.7. HRMS (ESI-TOF) m/z calcd for $C_{24}H_{24}O_3P$, 391.1458, [M + H]+, found 391.1459.

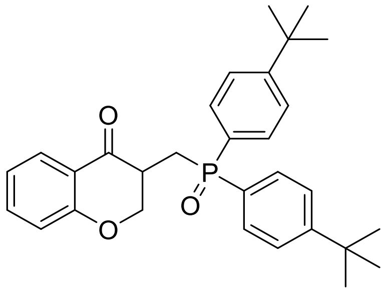

3-((Bis(4-(*tert*-butyl)phenyl)phosphoryl)methyl)chroman-4-one (**3ad**).

Oil, 85 mg, 60 % yield; ^{1}H NMR (600 MHz, DMSO-D6) δ 8.02 – 7.36 (m, 10H), 7.06 (t, *J* = 7.4 Hz, 1H), 7.00 (d, *J* = 8.3 Hz, 1H), 4.71 (dd, *J* = 11.3, 5.2 Hz, 1H), 4.38 (t, *J* = 11.5 Hz, 1H), 3.13 (m, 1H), 2.99 (d, *J* = 8.6 Hz, 1H), 2.43 – 2.10 (m, 1H), 1.26 (s, 9H), 1.25 (s, 9H). ^{13}C NMR (150 MHz, DMSO-D6) δ 192.2 (d, *J* = 12.3 Hz), 161.6, 155.0, 136.7, 131.6 (d, *J* = 100.2 Hz), 130.9 (d, *J* = 9.5 Hz), 130.5 (d, *J* = 9.9 Hz), 130.1 (d, *J* = 99.4 Hz), 127.3, 126.2 (d, *J* = 11.6 Hz), 126.0 (d, *J* = 11.7 Hz), 121.9, 120.3 118.2, 70.3, 40.8, 35.1, 31.2, 31.2, 24.6 (d, *J* = 72.9 Hz). ^{31}P NMR (243 MHz, DMSO-D6) δ 29.3. HRMS (ESI-TOF) m/z calcd for $C_{30}H_{36}O_3P$, 475.2397, [M + H]+, found 475.2398.

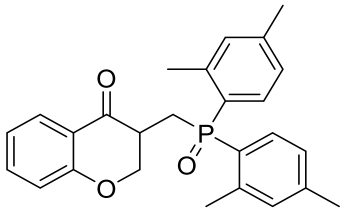

3-((Bis(2,4-dimethylphenyl)phosphoryl)methyl)chroman-4-one (**3ae**).

Oil, 65 mg, 52 % yield; ^{1}H NMR (600 MHz, DMSO-D6) δ 7.74 (dd, *J* = 7.9, 1.7 Hz, 1H), 7.55 (ddd, *J* = 8.6, 7.2, 1.7 Hz, 1H), 7.43 (d, *J* = 11.6 Hz, 4H), 7.18 (d, *J* = 4.3 Hz, 2H), 7.10 – 7.03 (m, 1H), 7.00 (d, *J* = 8.3 Hz, 1H), 4.65 (dd, *J* = 11.4, 5.1 Hz, 1H), 4.34 (t, *J* = 11.4 Hz, 1H), 3.06 (ddd, *J* = 15.5, 9.3, 2.5 Hz, 1H), 3.02 – 2.93 (m, 1H), 2.33 – 2.28 (m, 13H). ^{13}C NMR (150 MHz, DMSO-D6) δ 192.3 (d, *J* = 12.2 Hz), 161.6, 138.5 (d, *J* = 6.8 Hz), 138.4 (d, *J* = 6.9 Hz), 136.6, 134.6 (d, *J* = 97.4 Hz), 133.6 (d, *J* = 2.5 Hz), 133.6 (d, *J* = 2.6 Hz), 133.2 (d, *J* = 96.6 Hz), 128.4 (d, *J* = 9.1 Hz), 128.1 (d, *J* = 9.4 Hz), 127.3, 121.9, 120.3, 118.2, 70.3, 40.8, 24.6 (d, *J* = 72.3 Hz), 21.3, 21.2. ^{31}P NMR (243 MHz, DMSO-D6) δ 29.4. HRMS (ESI-TOF) m/z calcd for $C_{26}H_{28}O_3P$, 419.1771, [M + H]+, found 419.1772.

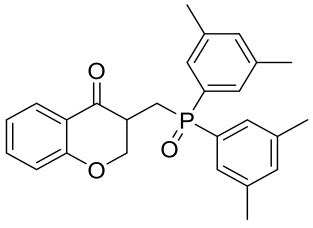

3-((Bis(3,5-dimethylphenyl)phosphoryl)methyl)chroman-4-one (**3af**).

Oil, 68 mg, 54% yield; ^{1}H NMR (600 MHz, DMSO -d_6) δ 7.75 (dd, *J* = 7.8, 1.2 Hz, 1H), 7.72 (dd, *J* = 12.2, 7.9 Hz, 1H), 7.61 (dd, *J* = 12.5, 7.9 Hz, 1H), 7.57 – 7.48 (m, 1H), 7.16 (t, *J* = 7.2 Hz, 2H), 7.12 – 6.92 (m, 4H), 4.69 (dd, *J* = 11.2, 5.3 Hz, 1H), 4.36 (t, *J* = 11.6 Hz, 1H), 3.13 (dd, *J* = 14.5, 8.6 Hz, 1H), 2.98 (q, *J* = 14.6 Hz, 1H), 2.41 – 2.31 (m, 1H), 2.29 (s, 3H), 2.27 (s, 3H), 2.21 (s, 3H), 2.19 (s, 3H). ^{13}C NMR (150 MHz, DMSO-d_6) δ 191.8 (d, *J* = 12.0 Hz), 161.1, 140.6 (d, *J* = 8.6 Hz), 140.5 (d, *J* = 8.3 Hz), 136.1, 132.4 (d, *J* = 10.8 Hz), 132.3 (d, *J* = 10.6 Hz), 132.1 (d, *J* = 10.1 Hz), 131.3 (d, *J* = 11.2 Hz), 128.9 (d, *J* = 97.8 Hz), 127.4 (d, *J* = 97.0 Hz), 126.8, 126.48 (d, *J* = 11.4 Hz), 126.40 (d, *J* = 11.7 Hz), 121.3, 119.8, 117.7, 69.9, 40.4, 23.3 (d, *J* = 72.3 Hz), 20.78 (d, *J* = 2.9 Hz), 20.76 (d, *J* = 2.9 Hz), 20.4 (d, *J* = 3.8 Hz), 20.3 (d, *J* = 4.2 Hz). ^{31}P NMR (243 MHz, DMSO-d6) δ 31.9. HRMS (ESI-TOF) m/z calcd for $C_{26}H_{28}O_3P$, 419.1771, [M + H]+, found 419.1772.

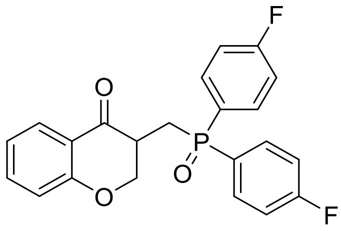 3-((Bis(4-fluorophenyl)phosphoryl)methyl)chroman-4-one (**3ag**).

Oil, 69 mg, 58% yield; ^{1}H NMR (600 MHz, DMSO-D6) δ 7.79 – 7.69 (m, 5H), 7.61 (qd, *J* = 8.6, 3.5 Hz, 2H), 7.55 (ddd, *J* = 8.7, 7.3, 1.7 Hz, 1H), 7.44 (td, *J* = 8.5, 4.2 Hz, 2H), 7.18 – 7.03 (m, 1H), 7.00 (d, *J* = 8.3 Hz, 1H), 4.68 (dd, *J* = 11.4, 5.1 Hz, 1H), 4.38 (t, *J* = 11.3 Hz, 1H), 3.24 (ddd, *J* = 15.7, 9.3, 3.0 Hz, 1H), 3.15 – 2.98 (m, 1H), 2.64 – 2.33 (m, 1H). ^{13}C NMR (150 MHz, DMSO-D6) δ 192.0 (d, *J* = 11.9 Hz), 162.56 (dd, *J* = 247.7, 6.7 Hz), 162.45 (dd, *J* = 247.8, 6.8 Hz), 161.5, 136.97 (dd, *J* = 96.9, 5.5 Hz), 136.6, 135.68 (dd, *J* = 96.9, 5.5 Hz), 131.85 (dd, *J* = 13.3, 6.3 Hz), 131.81 (dd, *J* = 13.3, 7.1 Hz), 127.34(dd, *J* = 8.8, 2.8 Hz), 127.3, 127.03 (dd, *J* = 8.8, 2.8 Hz), 121.9, 120.3, 119.6 (d, *J* = 21.0 Hz), 118.2, 117.75 (dd, *J* = 22.3, 10.6 Hz), 117.4 (dd, *J* = 22.3, 10.7 Hz), 70.3, 40.6 (d, *J* = 2.5 Hz), 24.5 (d, *J* = 73.1 Hz). ^{19}F NMR (565 MHz, DMSO-D6) δ -111.16 (d, *J* = 5.7 Hz), -111.18 (d, *J* = 5.7 Hz). ^{31}P NMR (243 MHz, DMSO-D6) δ 28.3 (t, *J* = 5.5 Hz). HRMS (ESI-TOF) m/z calcd for $C_{22}H_{18}O_{3}PF_{2}$, 399.0956, [M + H]+, found 399.0958.

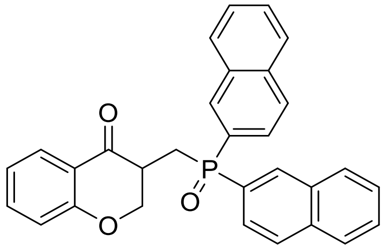

3-((Di(naphthalen-2-yl)phosphoryl)methyl)chroman-4-one (**3ah**).

White solid, 69 mg, 50 % yield; ^{1}H NMR (600 MHz, DMSO-D6) δ 8.60 (dd, *J* = 12.9, 8.9 Hz, 2H), 8.10 (d, *J* = 7.9 Hz, 2H), 8.06 (dd, *J* = 8.4, 2.1 Hz, 2H), 7.98 (t, *J* = 7.0 Hz, 2H), 7.90 (dd, *J* = 19.2, 9.4 Hz, 2H), 7.73 (dd, *J* = 7.9, 1.5 Hz, 1H), 7.67 – 7.57 (m, 4H), 7.56 – 7.49 (m, 1H), 7.04 (t, *J* = 7.5 Hz, 1H), 6.99 (d, *J* = 8.3 Hz, 1H), 4.73 (dd, *J* = 11.3, 5.1 Hz, 1H), 4.43 (t, *J* = 11.4 Hz, 1H), 3.35 (ddd, *J* = 15.5, 9.1, 2.4 Hz, 1H), 3.22 – 3.01 (m, 1H), 2.58 (m, 1H). ^{13}C NMR (150 MHz, DMSO-D6) δ 192.2 (d, *J* = 12.0 Hz), 161.6, 136.6, 134.6 (d, *J* = 1.8 Hz), 134.6 (d, *J* = 2.2 Hz), 132.9 (d, *J* = 8.5 Hz), 132.6 (d, *J* = 4.0 Hz), 132.5 (d, *J* = 3.8 Hz), 132.3 (d, *J* = 9.0 Hz), 131.8 (d, *J* = 98.1 Hz), 130.5 (d, *J* = 97.3 Hz), 129.5, 129.3, 129.0 (d, *J* = 11.5 Hz), 128.9 (d, *J* = 11.3 Hz), 128.7, 128.2, 127.7, 127.5 (d, *J* = 3.9 Hz), 127.3, 126.1 (d, *J* = 10.7 Hz), 126.0 (d, *J* = 10.5 Hz), 121.9, 120.3, 118.2, 70.4), 40.8, 24.6 (d, *J* = 73.0 Hz). ^{31}P NMR (243 MHz, DMSO-D6) δ 29.9. HRMS (ESI-TOF) m/z calcd for $C_{30}H_{24}O_{3}P$, 463.1458, [M + H]+, found 463.1458.

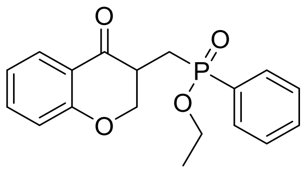 Ethyl ((4-oxochroman-3-yl)methyl)(phenyl)phosphinate (**3ai**).

Oil, 44 mg, 44 % yield; ^{1}H NMR (600 MHz, DMSO-D6) δ 7.86 – 7.46 (m, 7H), 7.18 – 6.88 (m,

2H), 4.68 (m, 1H), 4.35 (m, 1H), 4.09 – 3.70 (m, 2H), 3.29 – 2.83 (m, 1H), 2.75 – 2.54 (m, 1H), 2.13 – 1.81 (m, 1H), 1.22 (td, *J* = 7.0, 2.1 Hz, 3H). ^{13}C NMR (150 MHz, DMSO-D6) δ 192.0 (dd, *J* = 13.1, 11.7 Hz), 161.6 (d, *J* = 3.5 Hz), 136.6 (d, *J* = 3.5 Hz), 133.0 (d, *J* = 2.6 Hz), 131.8 (d, *J* = 9.9 Hz), 131.6 (d, *J* = 10.1 Hz), 130.8 (d, *J* = 122.6 Hz), 129.3 (dd, *J* = 12.1, 0.8 Hz), 127.3 (d, *J* = 6.5 Hz), 121.9 (d, *J* = 2.1 Hz), 120.3 (d, *J* = 8.6 Hz), 118.2 (d, *J* = 1.4 Hz), 70.2 (d, *J* = 9.2 Hz), 60.8 (d, *J* = 6.2 Hz), 40.7 (d, *J* = 3.3 Hz), 24.6 (dd, *J* = 101.9, 65.7 Hz), 16.7 (dd, *J* = 6.0, 1.6 Hz). ^{31}P NMR (243 MHz, DMSO-D6) δ 42.3 or 41.5. HRMS (ESI-TOF) m/z calcd for $C_{18}H_{20}O_4P$, 331.1094, [M + H]+, found 331.1092.

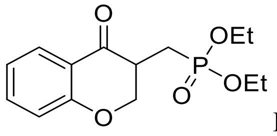

Diethyl ((4-oxochroman-3-yl)methyl)phosphonate (**3aj**).[5,6]

Oil, 25 mg, 28% yield; ^{1}H NMR (600 MHz, DMSO-D6) δ 7.78 (dd, *J* = 7.8, 1.7 Hz, 1H), 7.59 (ddd, *J* = 8.9, 7.2, 1.7 Hz, 1H), 7.08 (ddd, *J* = 16.6, 11.4, 4.6 Hz, 2H), 4.72 (dd, *J* = 11.3, 5.3 Hz, 1H), 4.36 (t, *J* = 11.7 Hz, 1H), 4.16 – 3.85 (m, 4H), 3.24 – 3.01 (m, 1H), 2.40 (ddd, *J* = 18.9, 15.8, 3.2 Hz, 2H), 1.83 (td, *J* = 16.3, 9.8 Hz, 1H), 1.25 (t, *J* = 7.1 Hz, 6H). ^{13}C NMR (150 MHz, DMSO) δ 192.1 (d, *J* = 14.6 Hz), 161.6, 136.6, 127.3, 121.9, 120.3, 118.2, 70.2, 61.9 (d, *J* = 6.3 Hz), 61.7 (d, *J* = 6.3 Hz), 40.82, 20.7 (d, *J* = 143.7 Hz), 16.6 (d, *J* = 5.8 Hz). ^{31}P NMR (243 MHz, DMSO-D6) δ 29.4. HRMS (ESI-TOF) m/z calcd for $C_{14}H_{20}O_5P$, 299.1043, [M + H]+, found 299.1048.

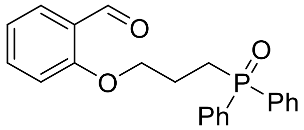

2-(3-(Diphenylphosphoryl)propoxy)benzaldehyde, **4**. Colorless oil. 8% yield. 9 mg. ^{1}H NMR (600 MHz, DMSO-D6) δ 10.40 (s, 1H), 7.89 – 7.79 (m, 4H), 7.69 (dd, *J* = 7.6, 1.6 Hz, 1H), 7.63 – 7.58 (m, 1H), 7.57 – 7.42 (m, 6H), 7.12 (d, *J* = 8.4 Hz, 1H), 7.05 (t, *J* = 7.4 Hz, 1H), 4.18 (t, *J* = 6.1 Hz, 2H), 2.85 – 2.55 (m, 2H), 1.96 (dd, *J* = 15.5, 9.9 Hz, 2H). ^{13}C NMR (150 MHz, DMSO-D6-d6) δ 189.9, 161.1, 136.7, 134.2 (d, *J* = 96.4 Hz), 132.0 (d, *J* = 2.1 Hz), 130.8 (d, *J* = 9.3 Hz), 129.1 (d, *J* = 11.2 Hz), 128.2, 124.7, 121.0, 113.7, 68.4 (d, *J* = 14.2 Hz), 25.3 (d, *J* = 72.3 Hz), 21.8 (d, *J* = 3.1 Hz). ^{31}P NMR (243 MHz, DMSO-d6) δ 30.3. HRMS (ESI-TOF) m/z calcd for $C_{22}H_{22}O_3P$, 365.1301, [M + H]+, found 365.1302.

5. References

[1] a) N. J. Parmar, B. D. Parmar, T. R. Sutariya, R. Kant, V. K. Gupta, *Tetrahedron Letters,* **2014**, 55, 6060-6064; b) K. Hirano, A. T. Biju, I. Piel and F. Glorius, *J. Am. Chem. Soc.* **2009**, *131*, 14190-14191; c) G. Bashiardes, I. Safir, A. S. Mohamed, F. Barbot, J. Laduranty, *Org. Lett.* **2003**, *5*, 4915-4918.

[2] D. Zhang, M. Lian, J. Liu, S. Tang, G. Liu, C. Ma, Q. Meng, H. Peng, D. Zhu, *Org. Lett.* **2019**, 21, 2597-2601.

[3] S. K. Thummanapelli, S. Hosseyni, Y. Su, N. G. Akhmedov, X. Shi, *Chem. Commun.* **2016**, 52, 7687-7690.

[4] R. Rohlmann, C.-G. Daniliuc, O. G. Mancheno, *Chem. Commun.* **2013**, *49*, 11665-11667.

[5] S. C. Cullen and T. Rovis, *Org. Lett.*, 2008, **10**, 3141-3144.

[6] J. Zhao, P. Li, X. Li, C. Xia and F. Li. *Chem. Commun.*, 2016, **52**, 3661-3664.

6. ^{1}H NMR, ^{13}C NMR, ^{31}P NMR and ^{19}F NMR spectra for products

Compound 3aa ^{1}H NMR, ^{13}C NMR and ^{31}P NMR

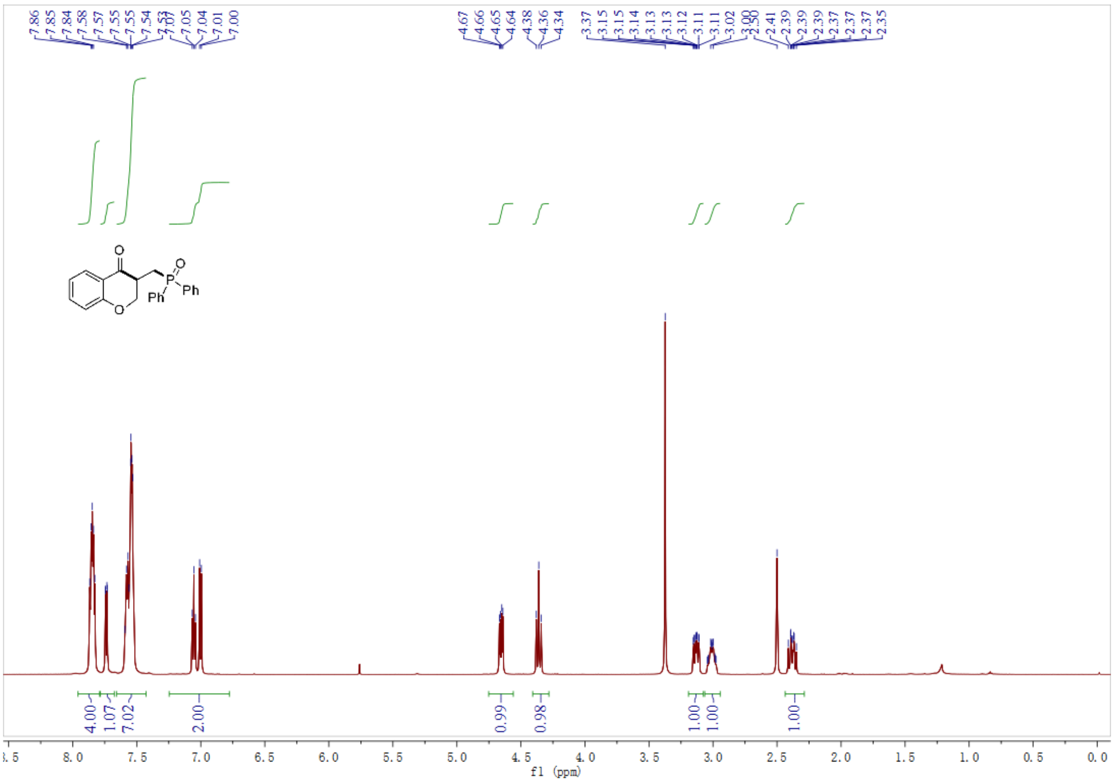

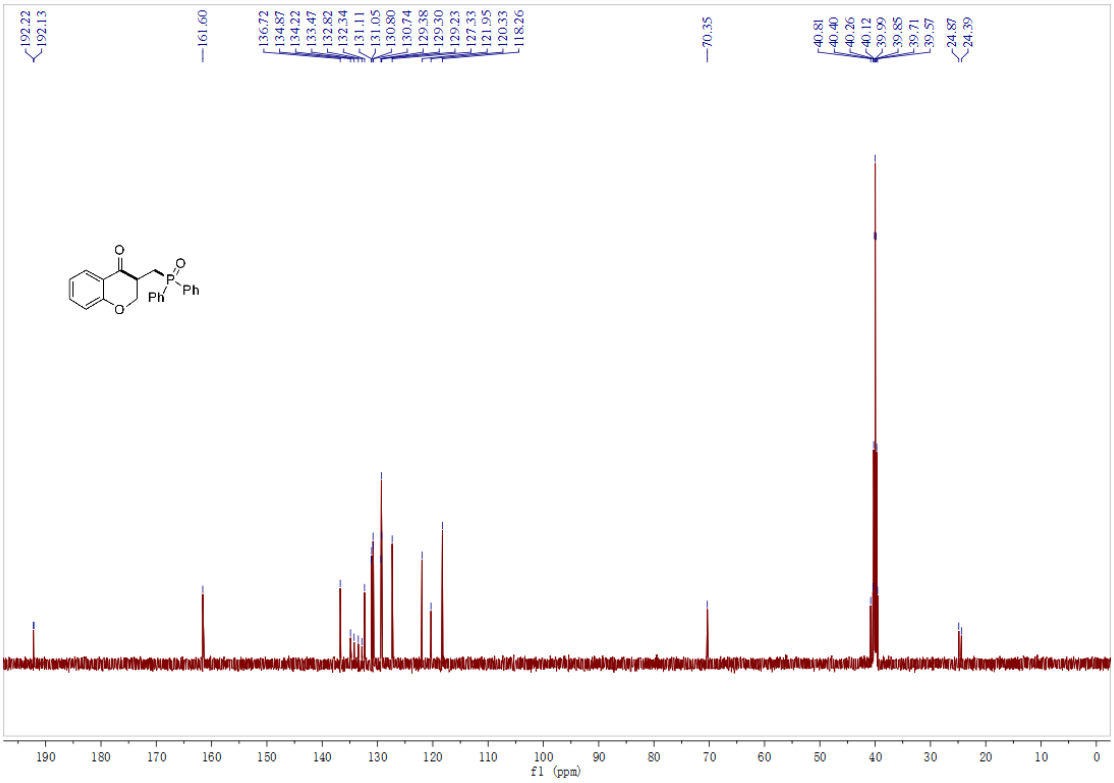

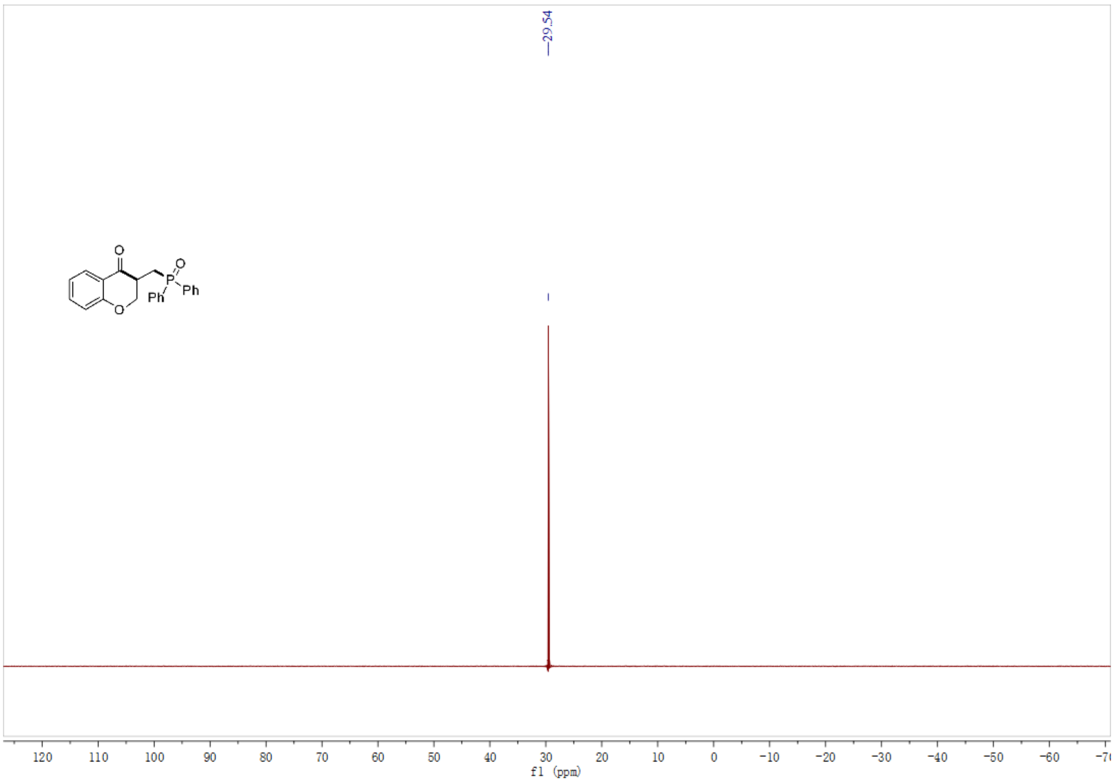

29.54
O
O
P
Ph
Ph
O
120 110 100 90 80 70 60 50 40 30 20 10 0 -10 -20 -30 -40 -50 -60
f1 (ppm)

Compound 3ba ^{1}H NMR, ^{13}C NMR and ^{31}P NMR

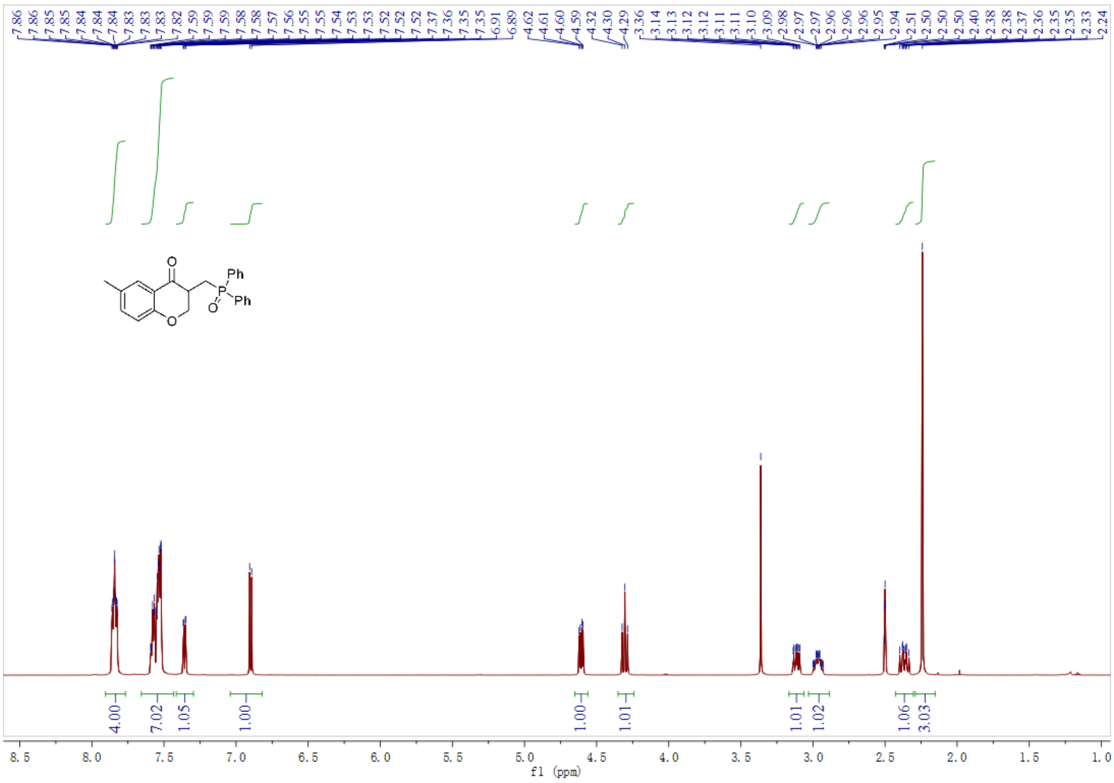

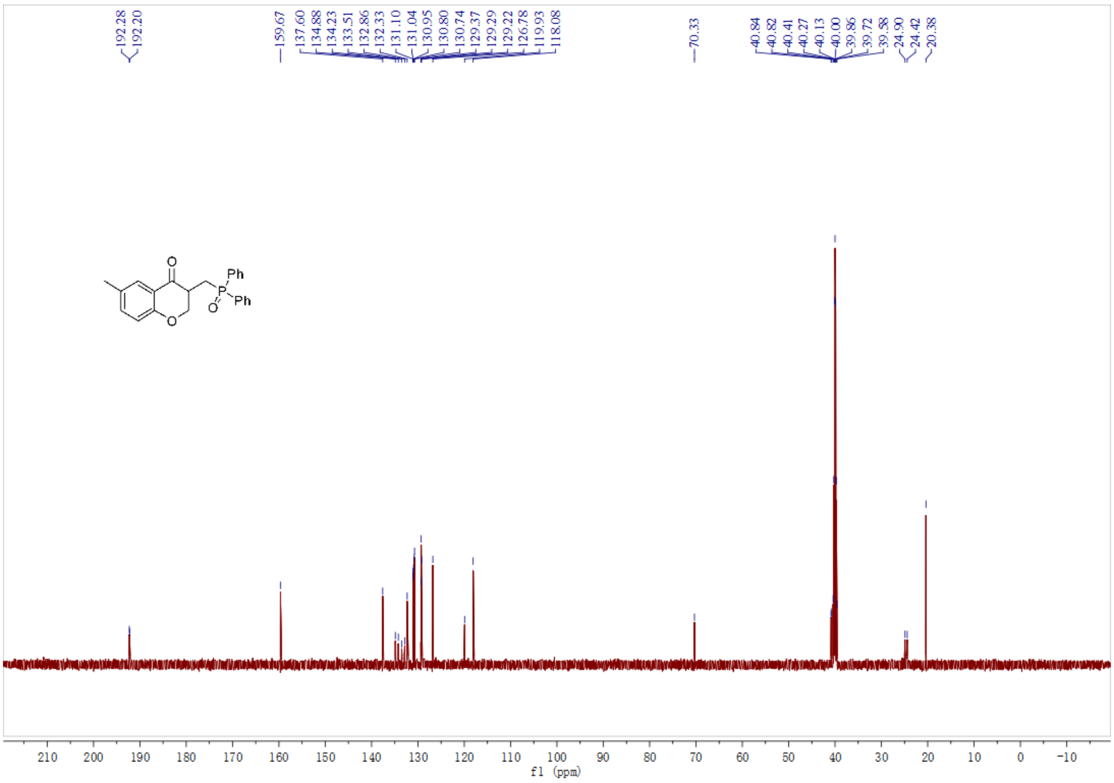

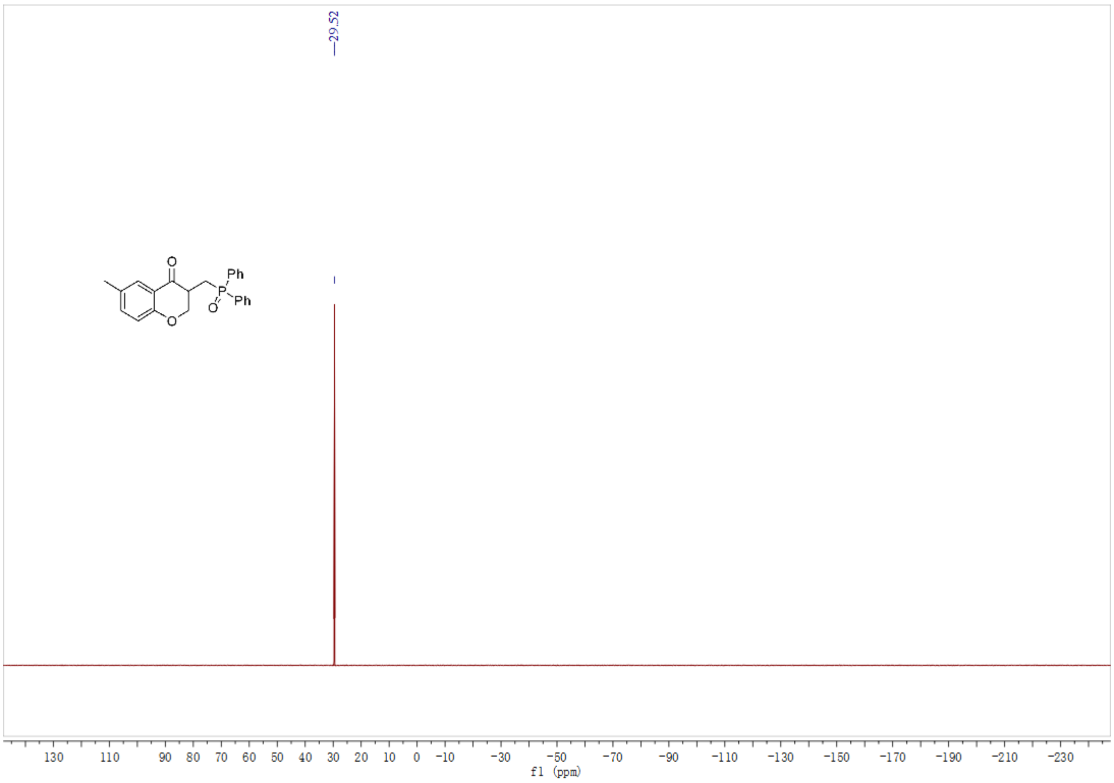

29.52
Ph
Ph
O
O
O
P
130
110
90
80
70
60
50
40
30
20
10
0
−10
−30
−50
−70
−90
−110
−130
−150
−170
−190
−210
−230
f1 (ppm)

Compound 3ca ^{1}H NMR, ^{13}C NMR and ^{31}P NMR

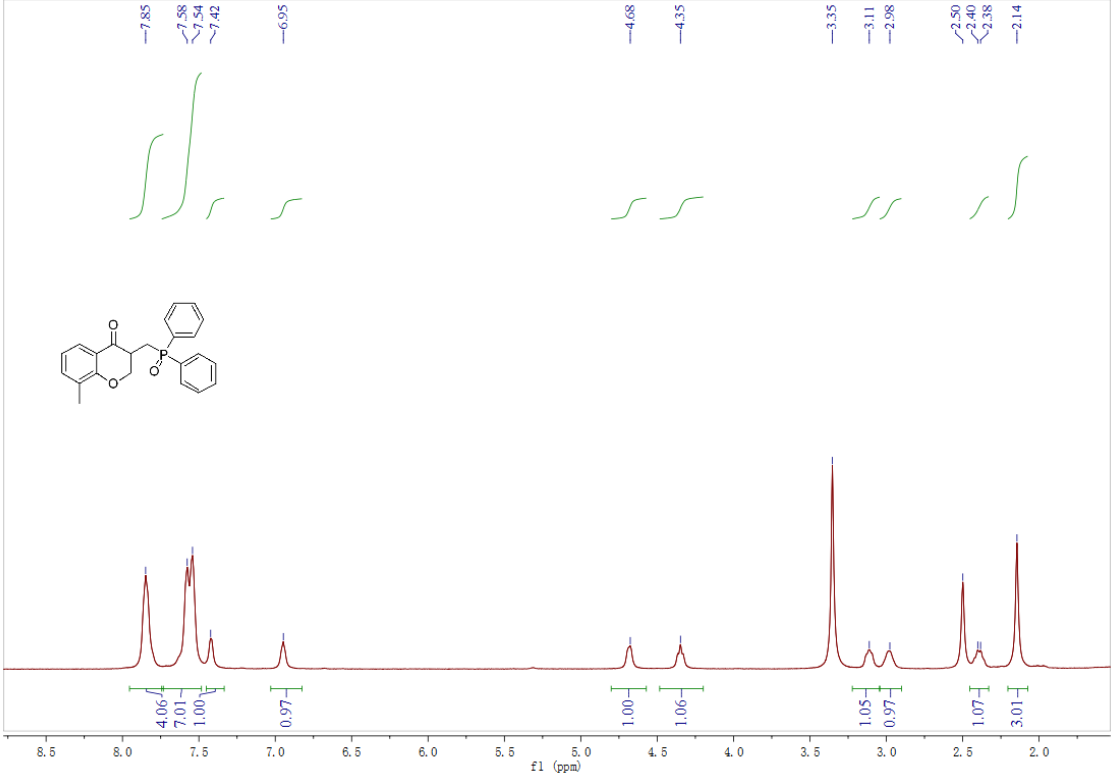

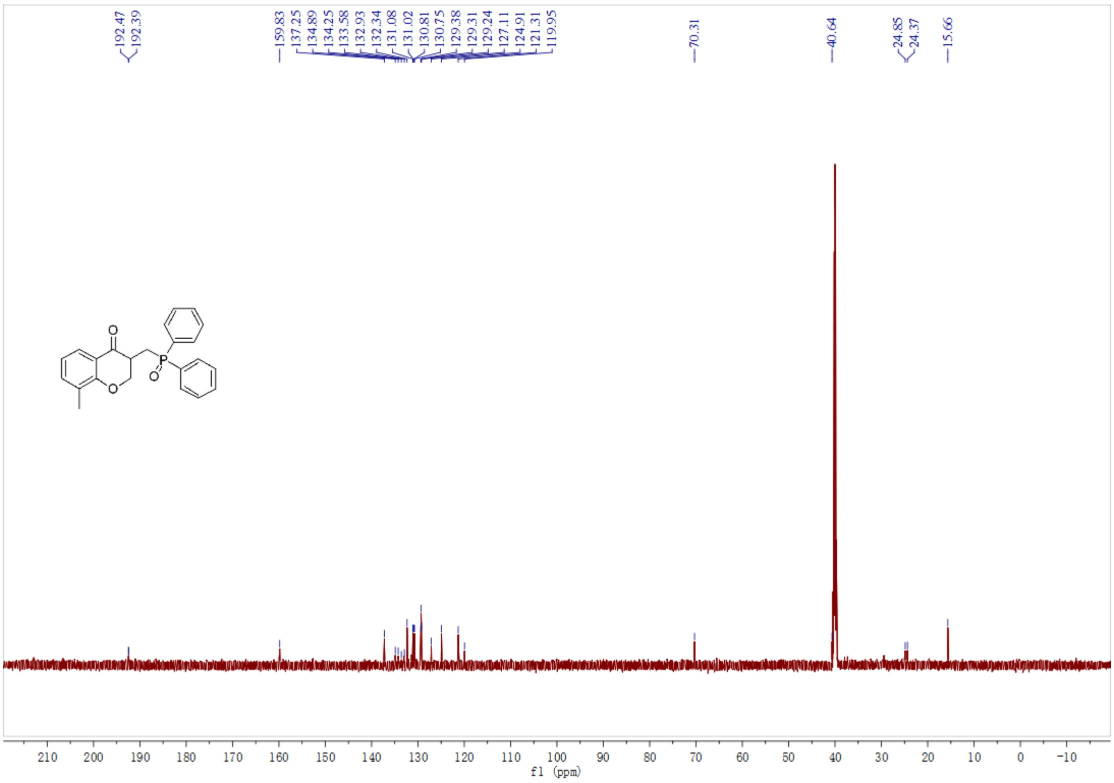

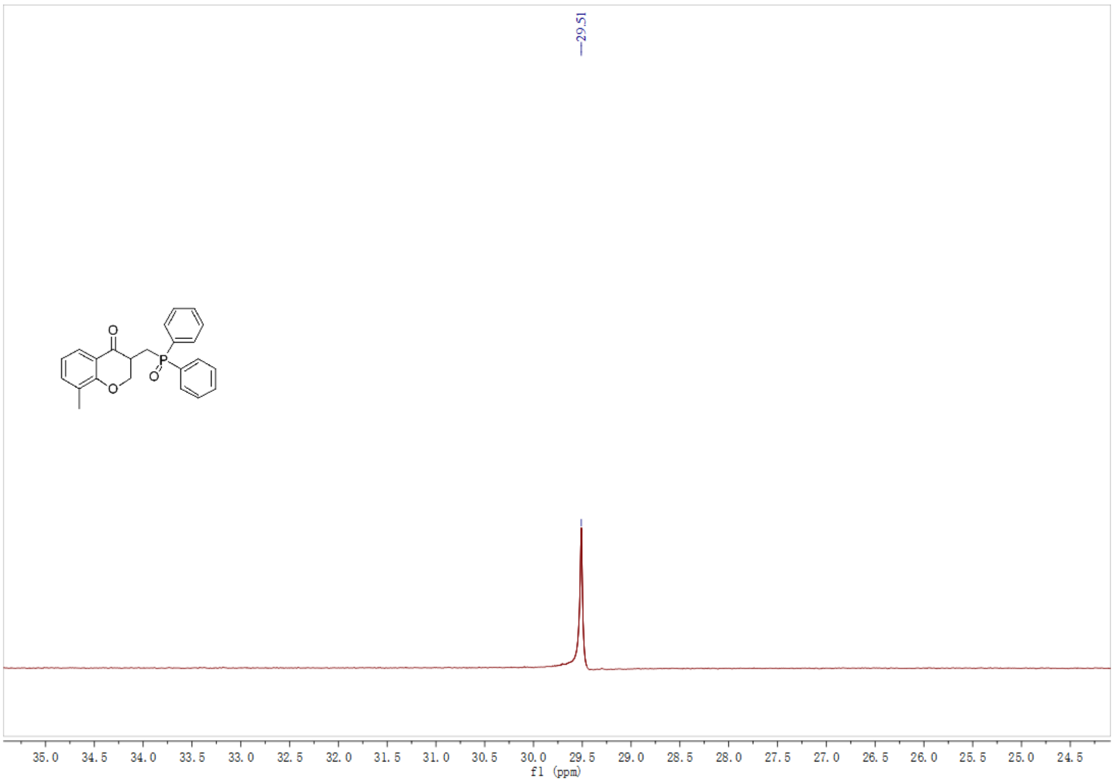
29.51
35.0 34.5 34.0 33.5 33.0 32.5 32.0 31.5 31.0 30.5 30.0 29.5 29.0 28.5 28.0 27.5 27.0 26.5 26.0 25.5 25.0 24.5
f1 (ppm)

Compound 3da ^{1}H NMR, ^{13}C NMR and ^{31}P NMR

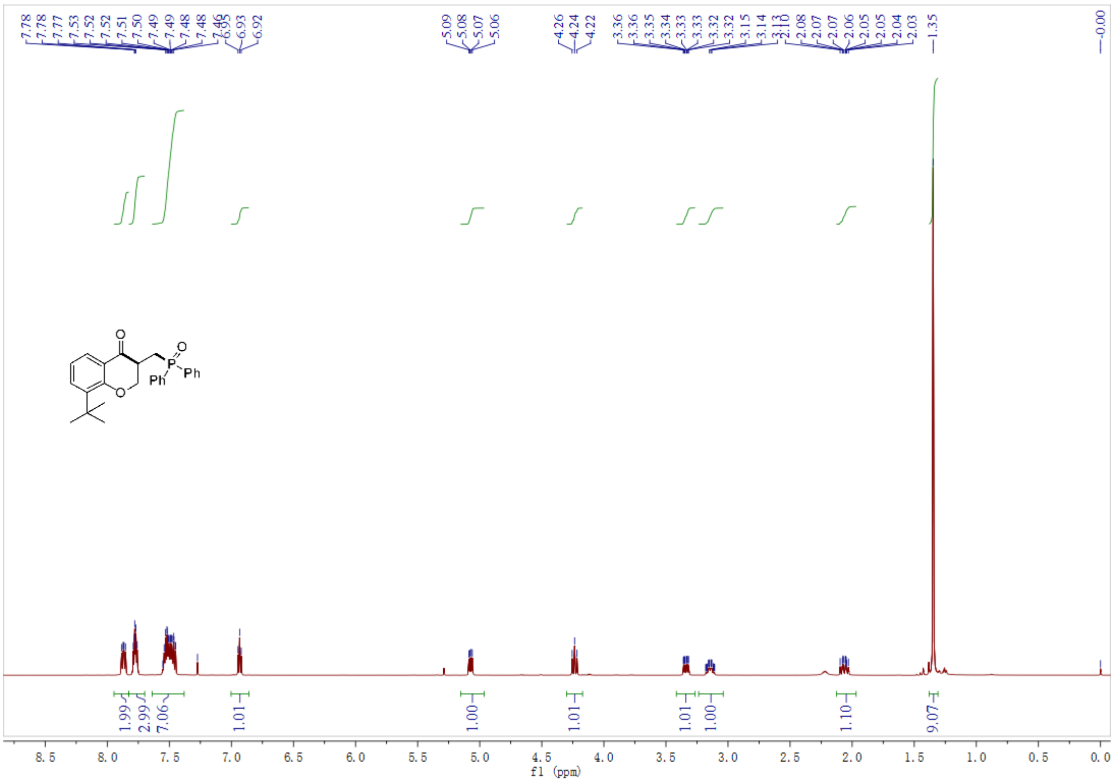

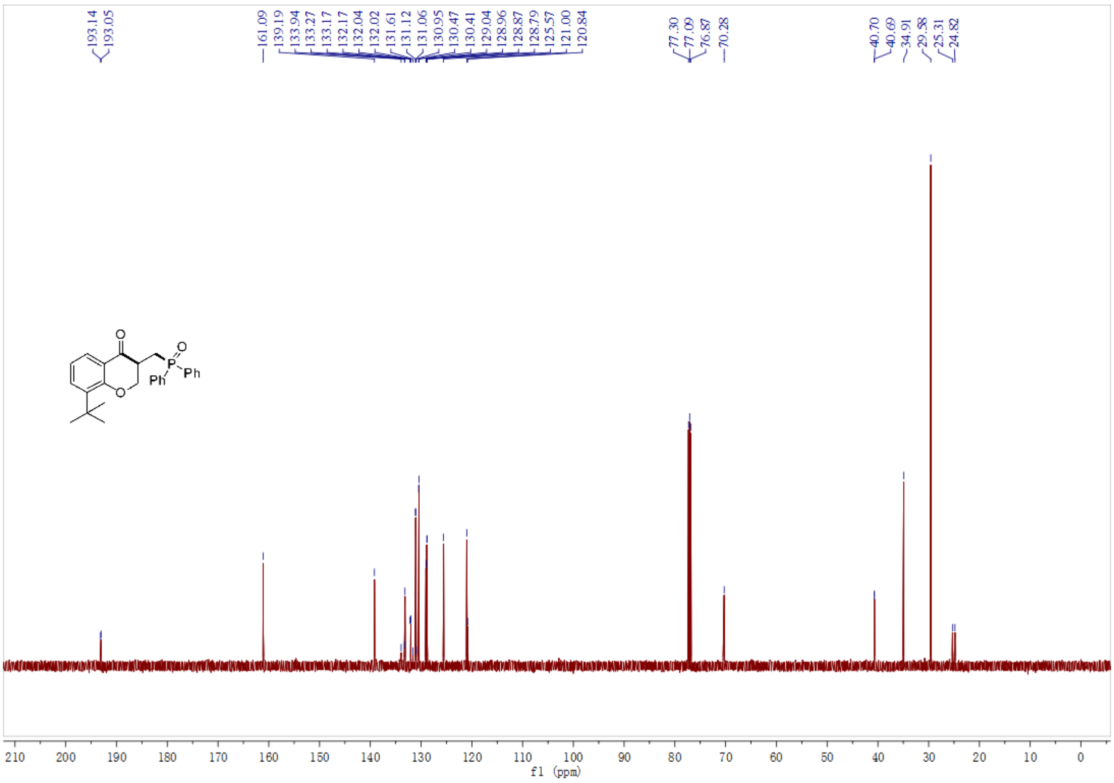

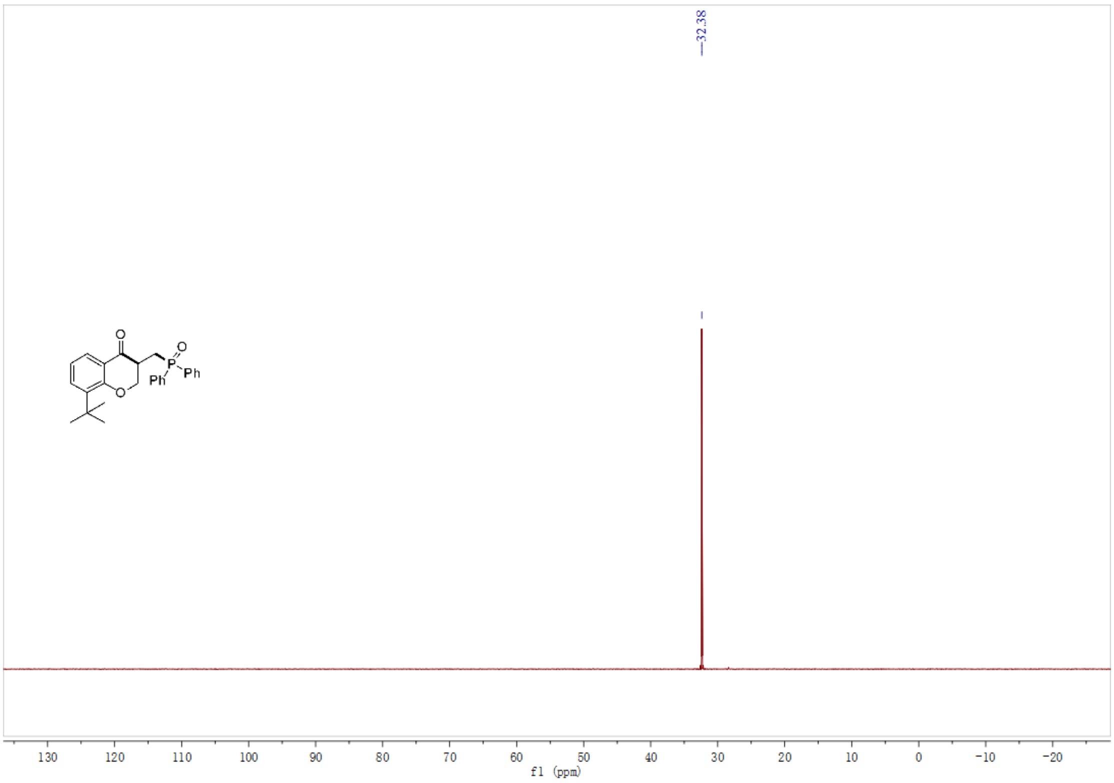

32.38
O
O
P
Ph
Ph
O
130
120
110
100
90
80
70
60
50
40
30
20
10
0
-10
-20
f1 (ppm)

Compound 3ea ^{1}H NMR, ^{13}C NMR and ^{31}P NMR

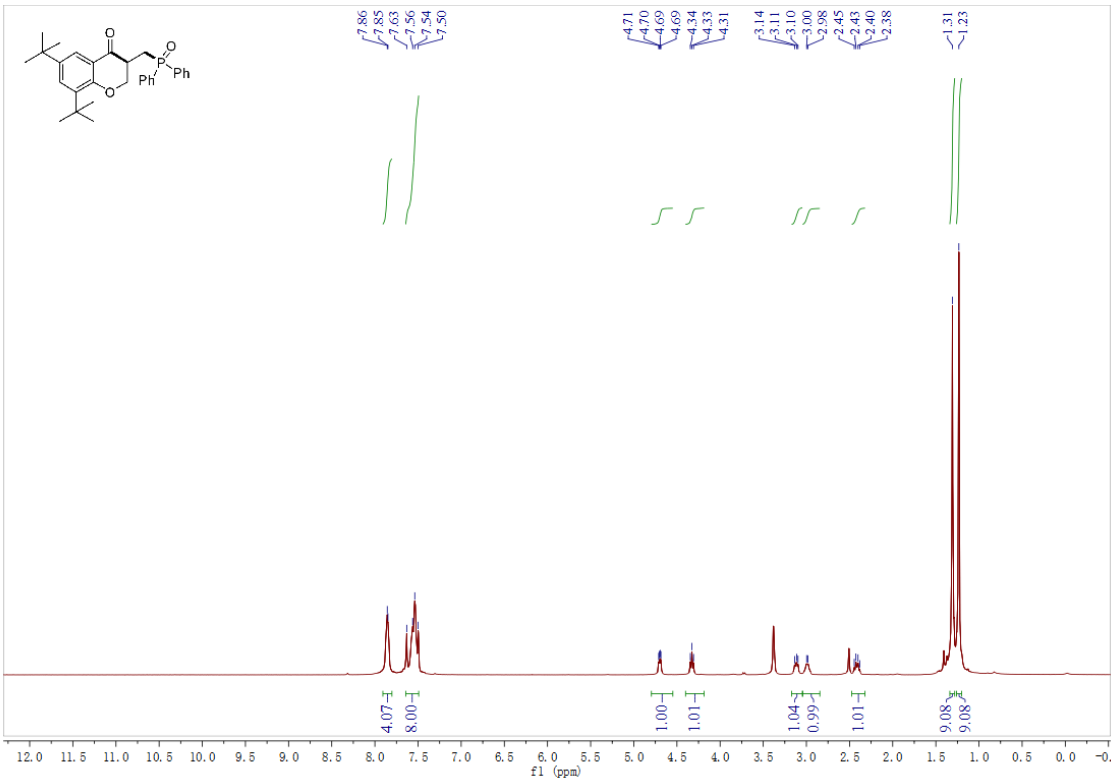

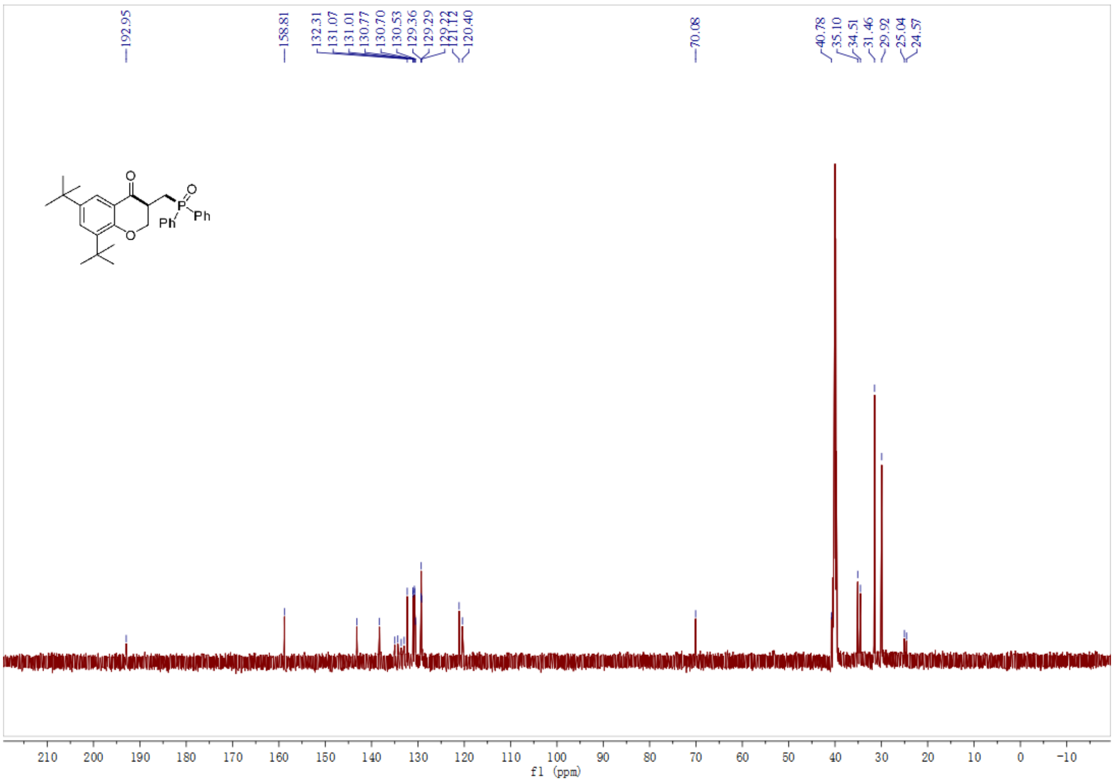

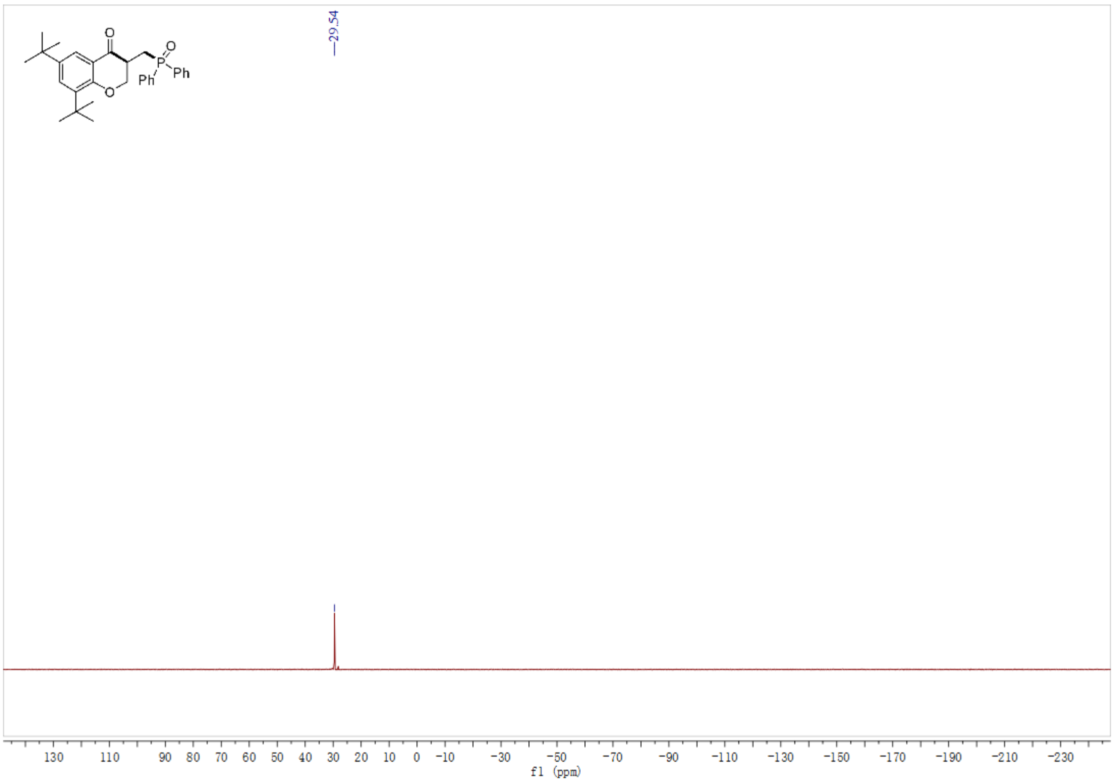

29.54
O
O
P
Ph
Ph
O
130 110 90 80 70 60 50 40 30 20 10 0 −10 −30 −50 −70 −90 −110 −130 −150 −170 −190 −210 −230
f1 (ppm)

Compound 3fa ^{1}H NMR, ^{13}C NMR and ^{31}P NMR

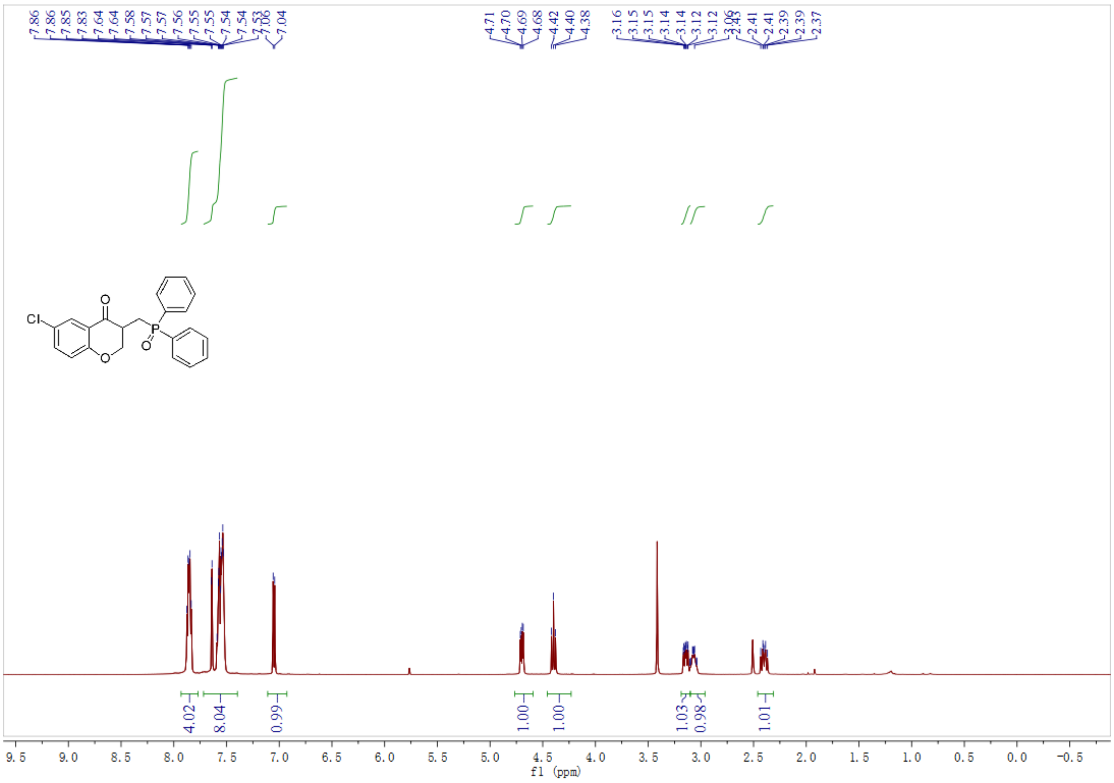

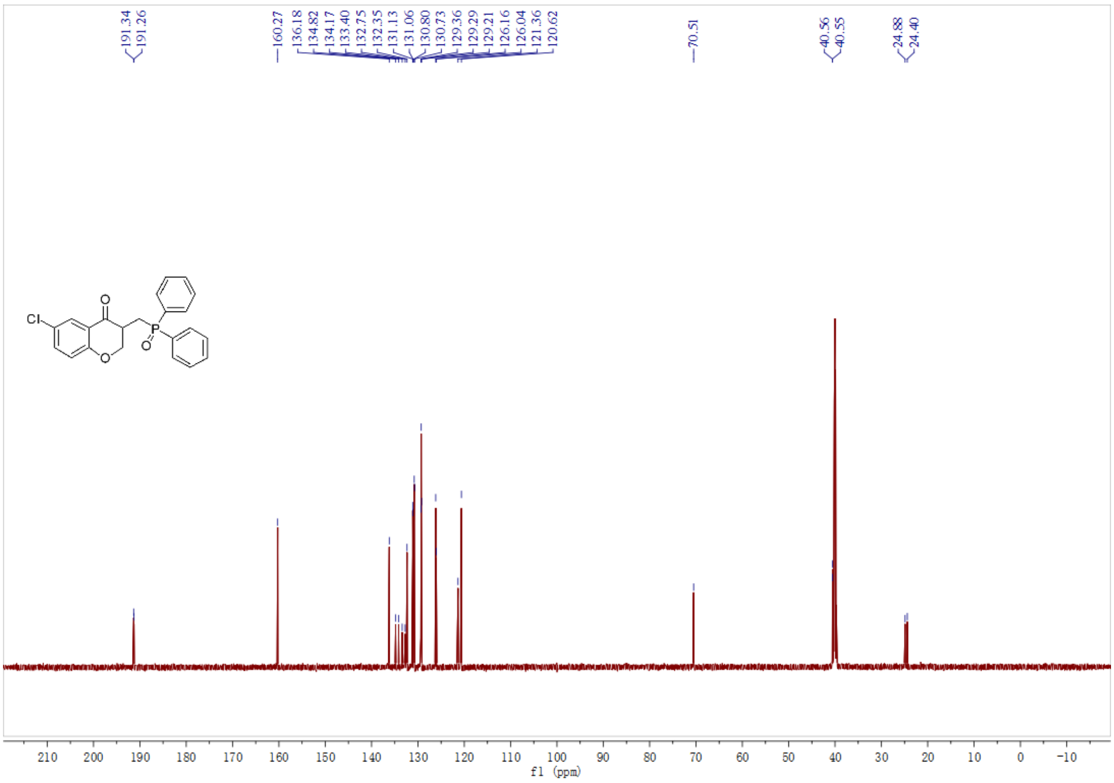

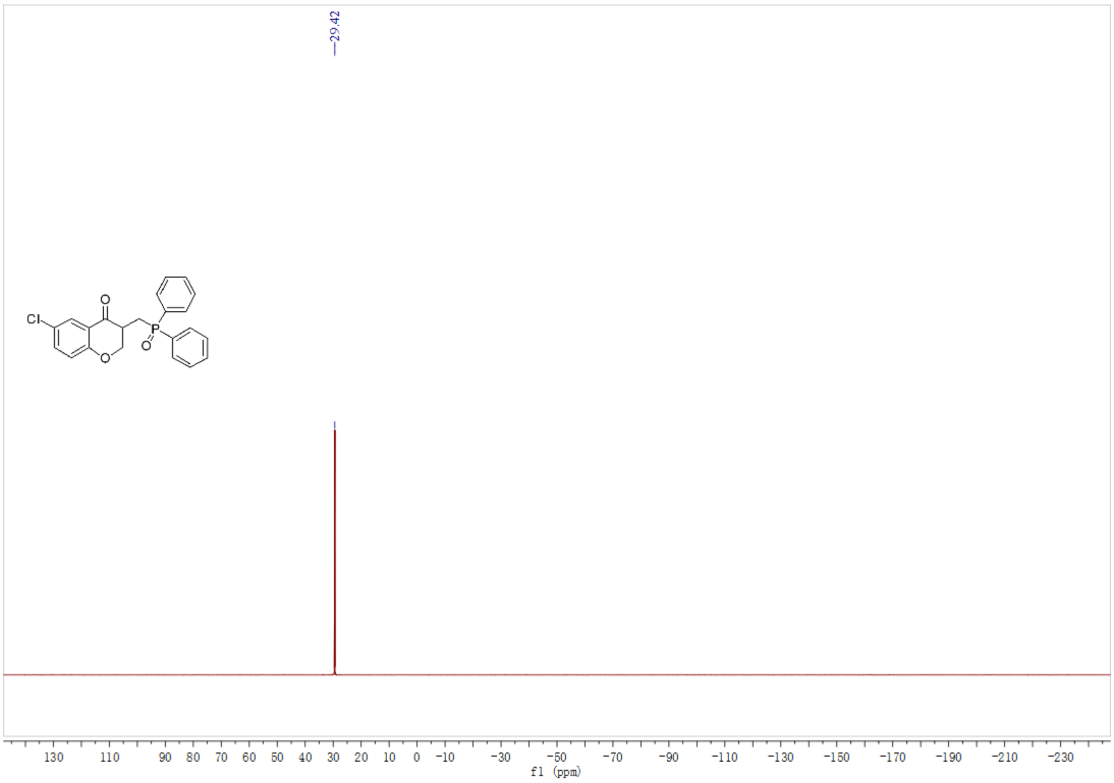

29.42
Cl
O
O
P
O
130 110 90 80 70 60 50 40 30 20 10 0 −10 −30 −50 −70 −90 −110 −130 −150 −170 −190 −210 −230
f1 (ppm)

Compound 3ga ^{1}H NMR, ^{13}C NMR and ^{31}P NMR

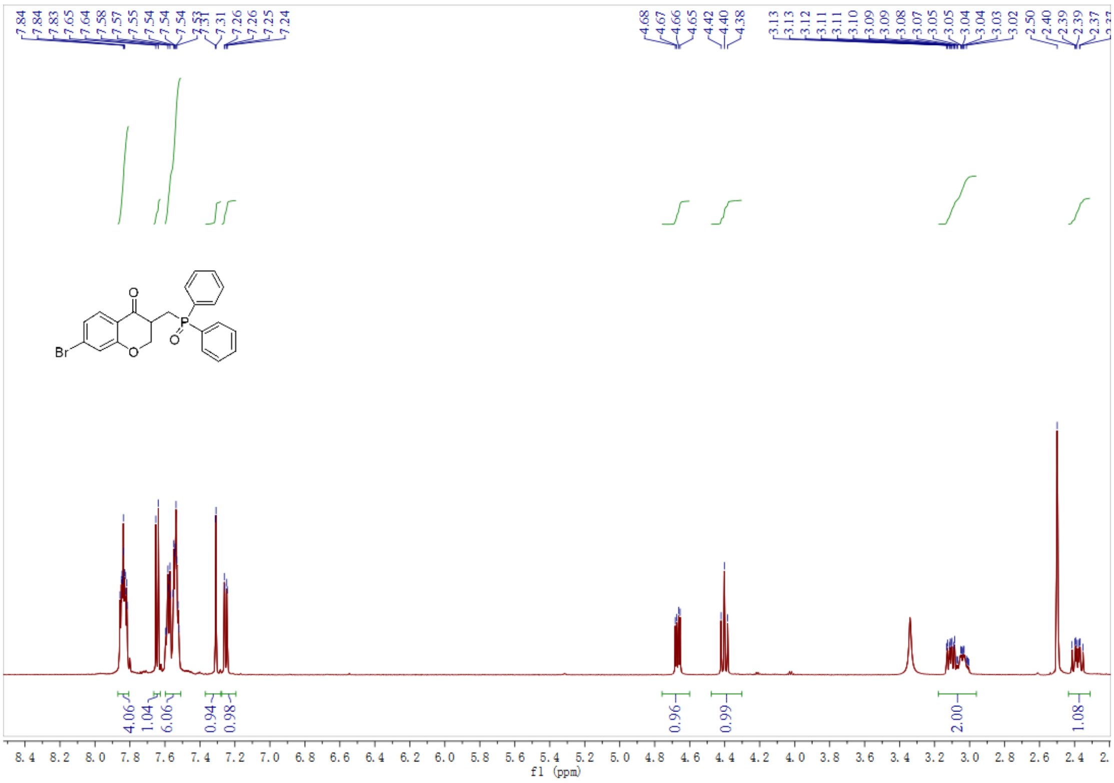

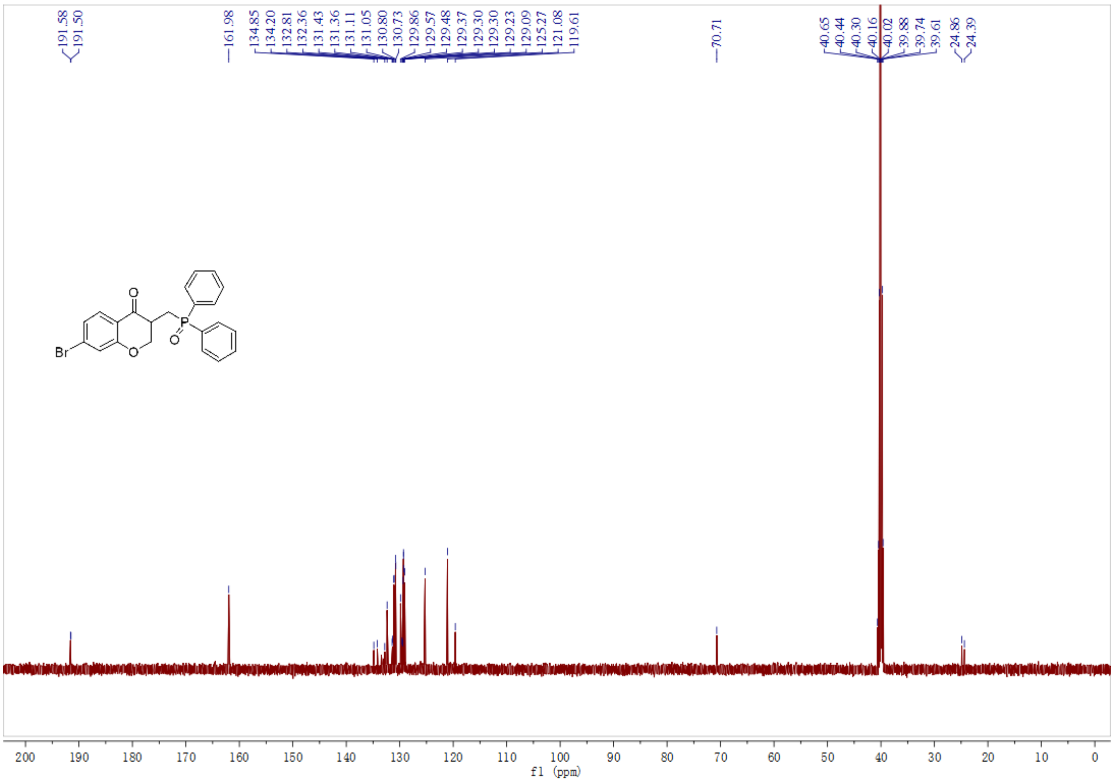

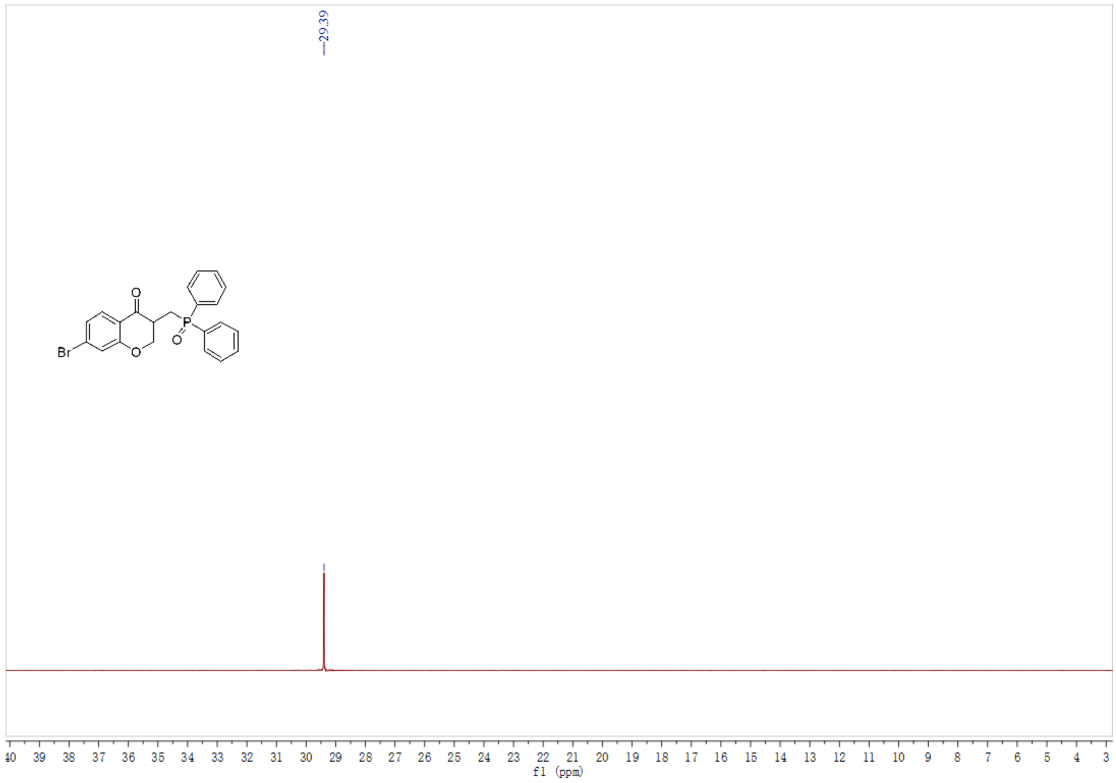

29.39
Br
O
O
P
O
f1 (ppm)

Compound 3ha ^{1}H NMR, ^{13}C NMR, ^{19}F NMR and ^{31}P NMR

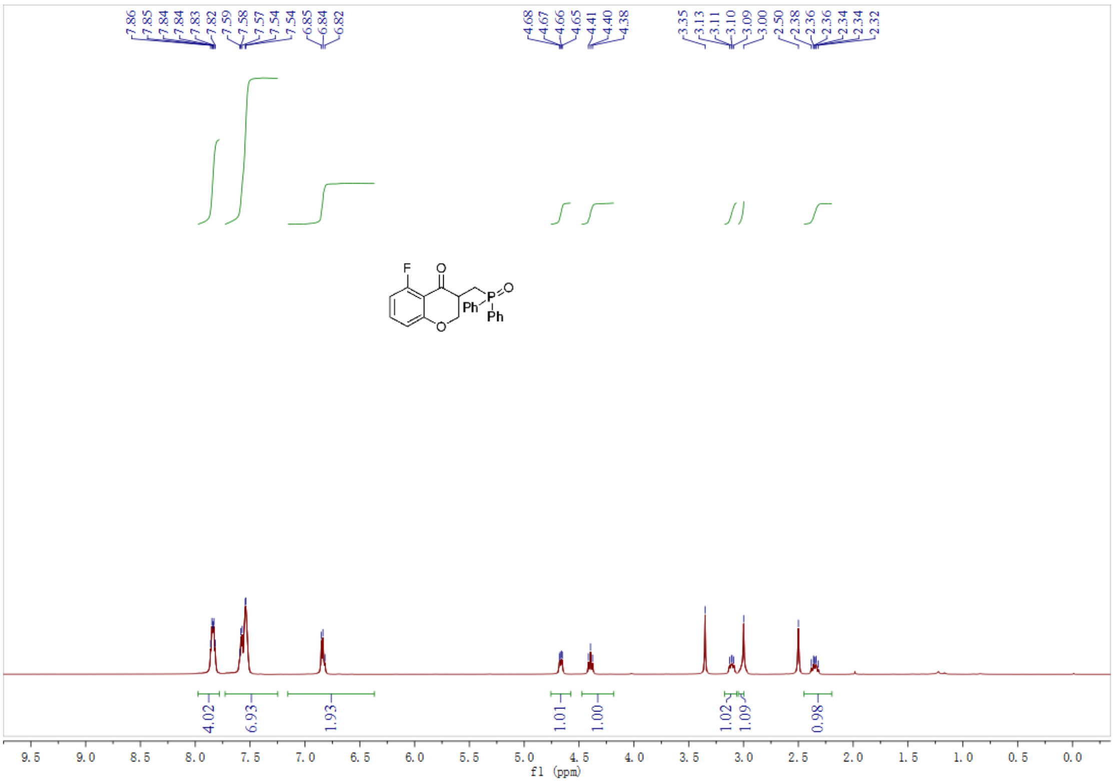

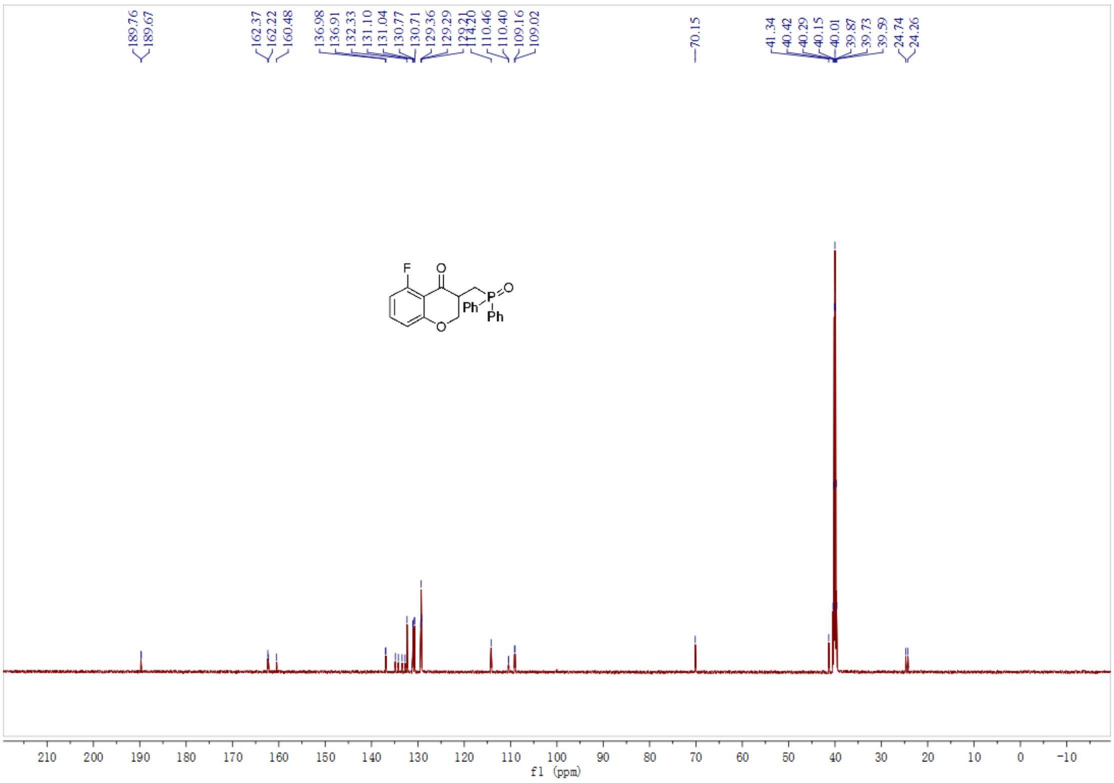

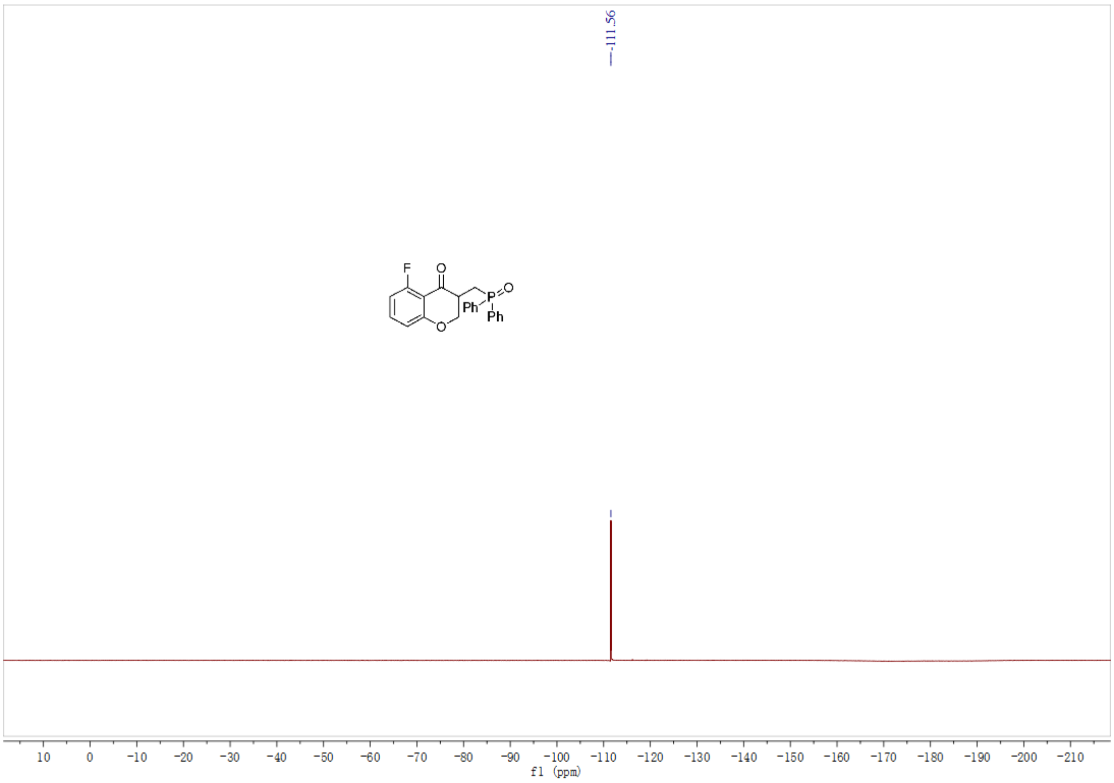
-111.56
F
O
Ph
P
O
Ph
O
10 0 −10 −20 −30 −40 −50 −60 −70 −80 −90 −100 −110 −120 −130 −140 −150 −160 −170 −180 −190 −200 −210
f1 (ppm)

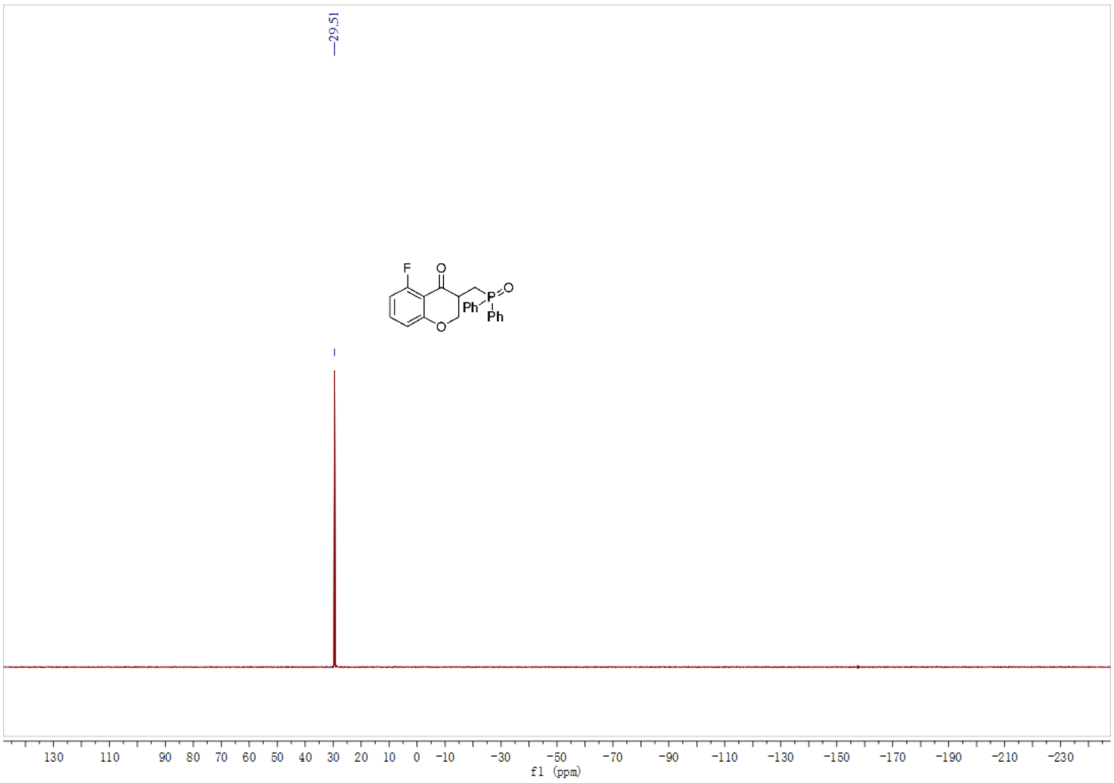
29.51
F
O
Ph
P
O
Ph
O
130 110 90 80 70 60 50 40 30 20 10 0 −10 −30 −50 −70 −90 −110 −130 −150 −170 −190 −210 −230
f1 (ppm)

Compound 3ia ^{1}H NMR, ^{13}C NMR and ^{31}P NMR

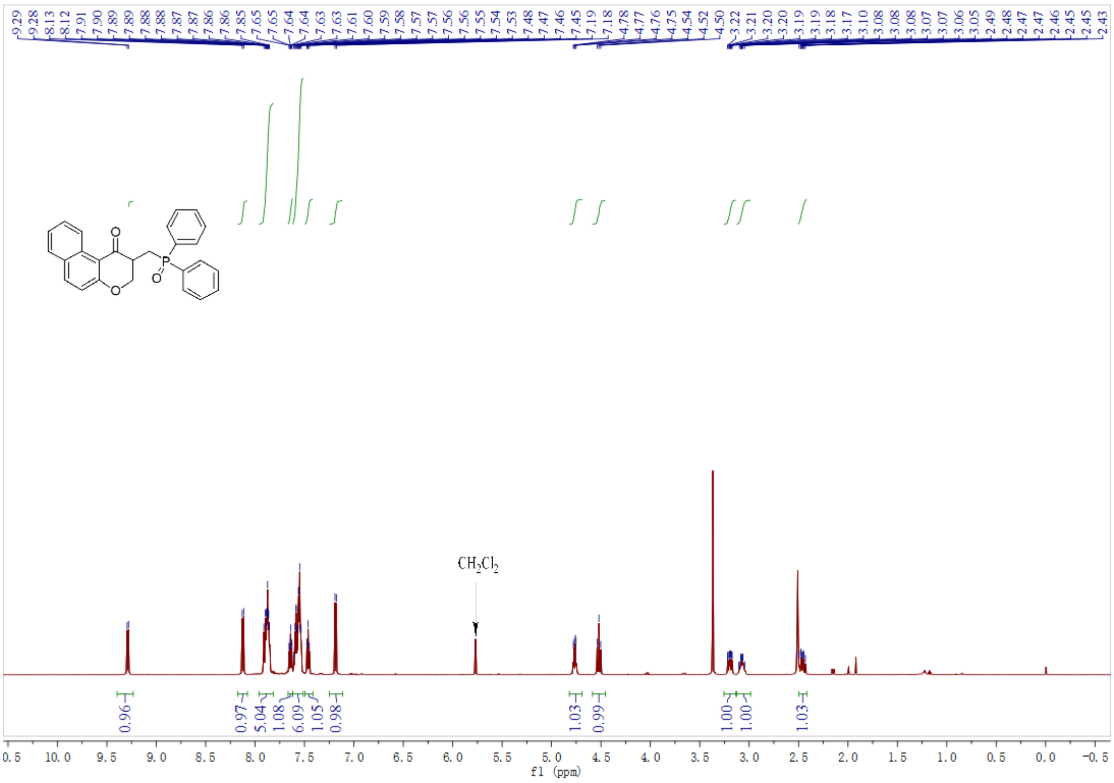

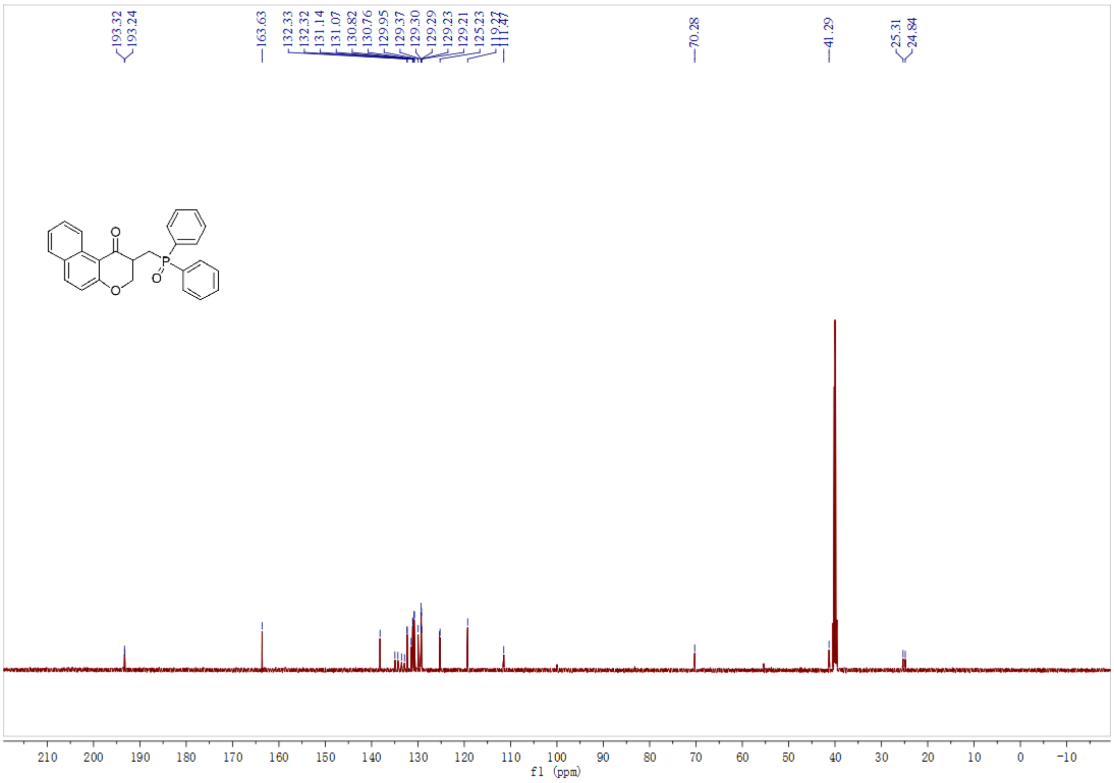

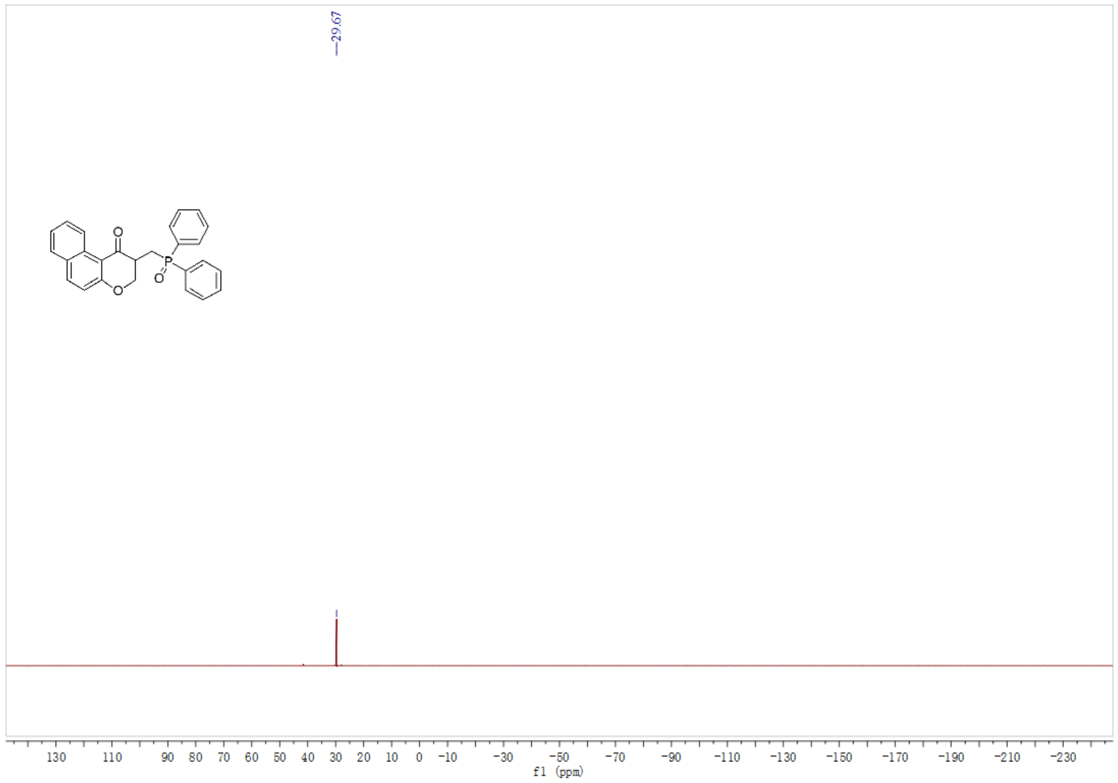

29.67
130
110
90
80
70
60
50
40
30
20
10
0
−10
−30
−50
−70
−90
−110
−130
−150
−170
−190
−210
−230
f1 (ppm)

Compound 3ja ^{1}H NMR, ^{13}C NMR and ^{31}P NMR

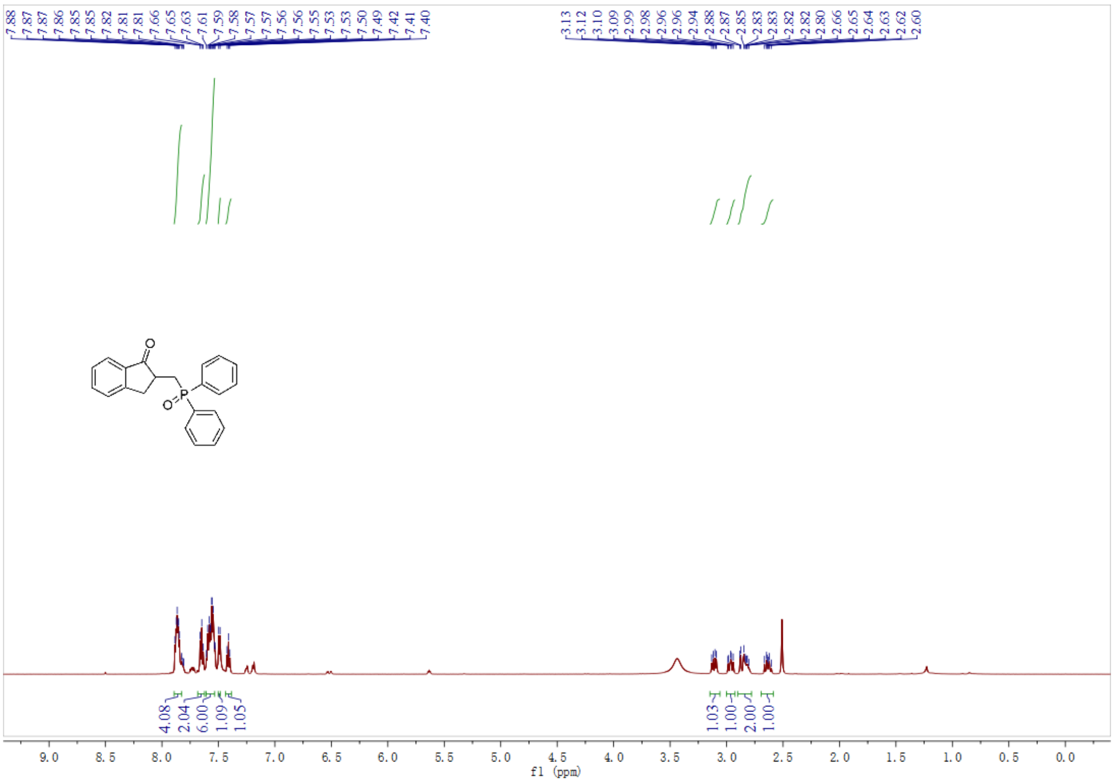

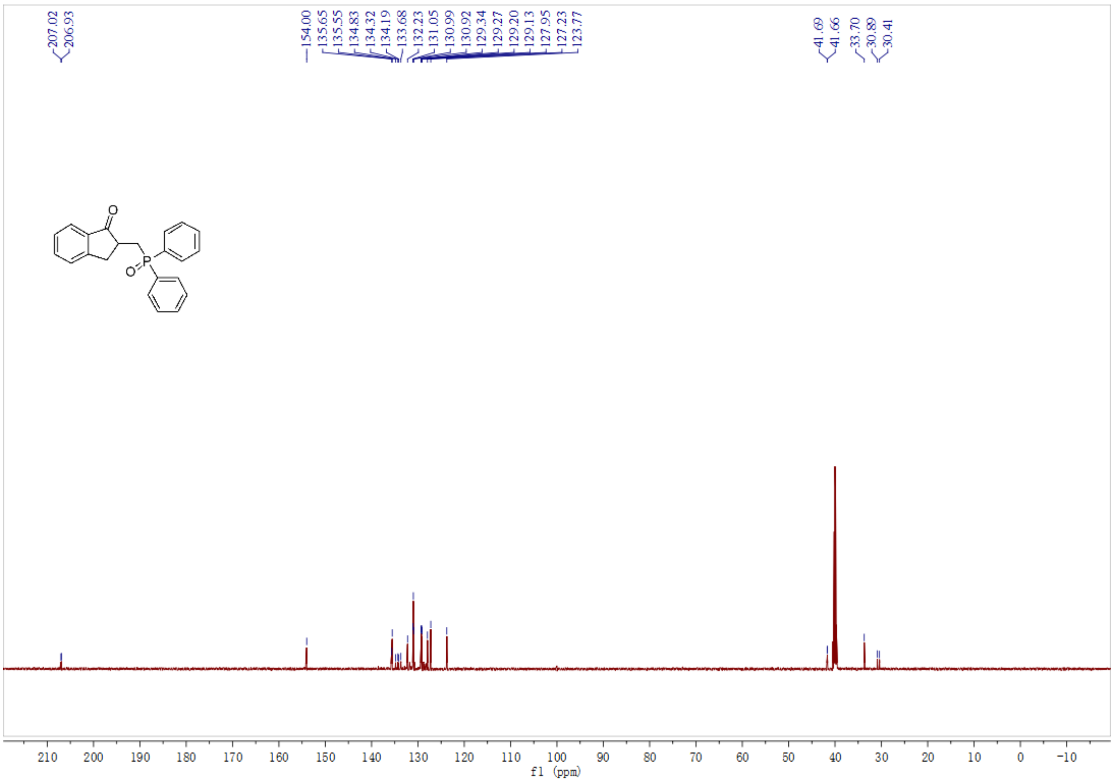

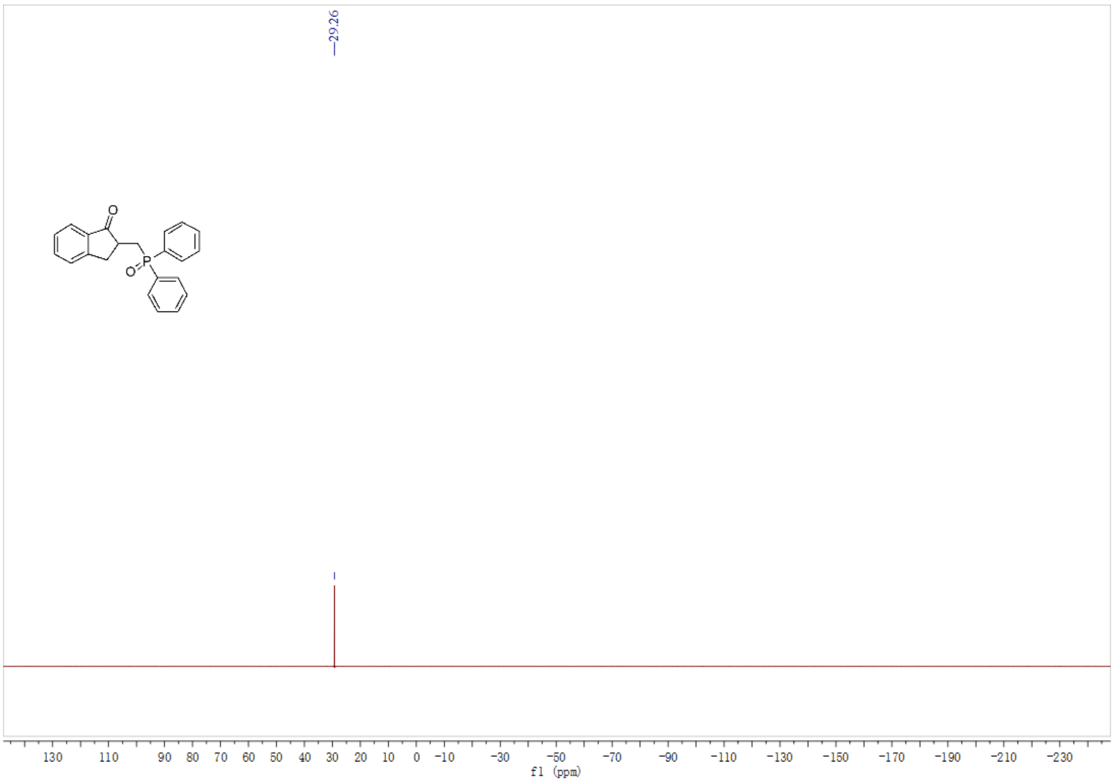

Compound 3la ^{1}H NMR, ^{13}C NMR and ^{31}P NMR

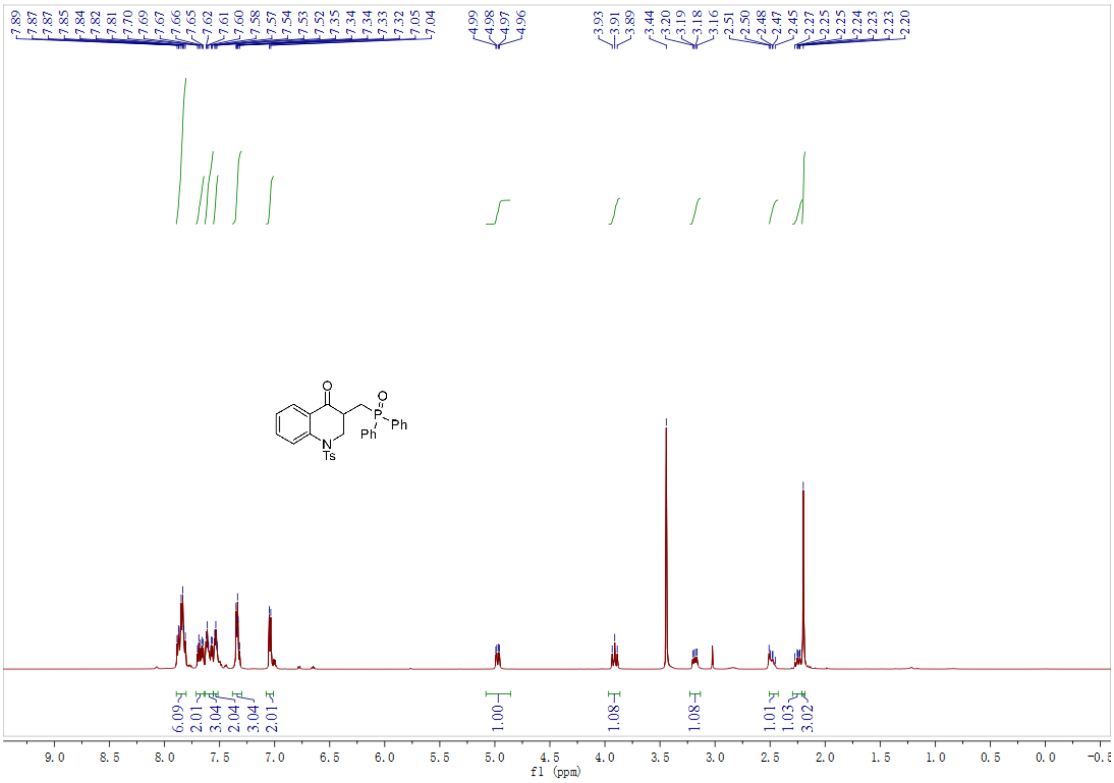

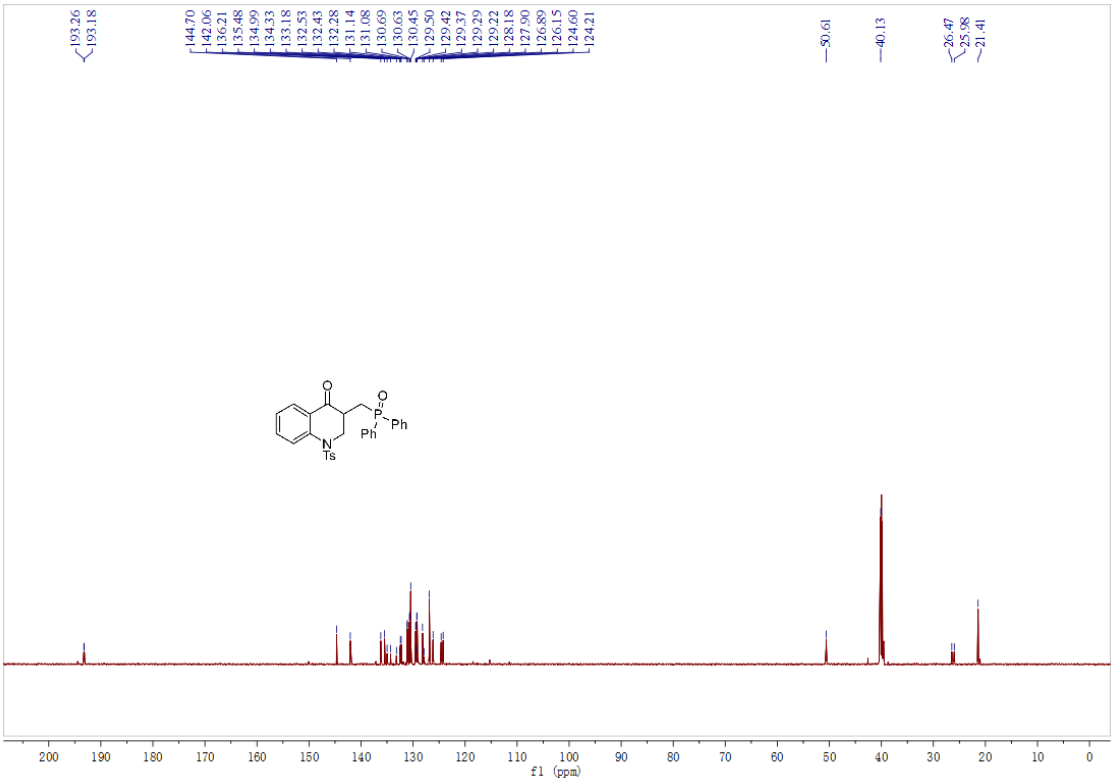
193.26
193.18
144.70
142.06
136.21
135.48
134.99
134.33
133.18
132.53
132.43
132.28
131.14
131.08
130.69
130.63
130.45
129.50
129.42
129.37
129.29
129.22
128.18
127.90
126.89
126.15
124.60
124.21
50.61
40.13
26.47
25.98
21.41
O
P
Ph
Ph
N
Ts
f1 (ppm)

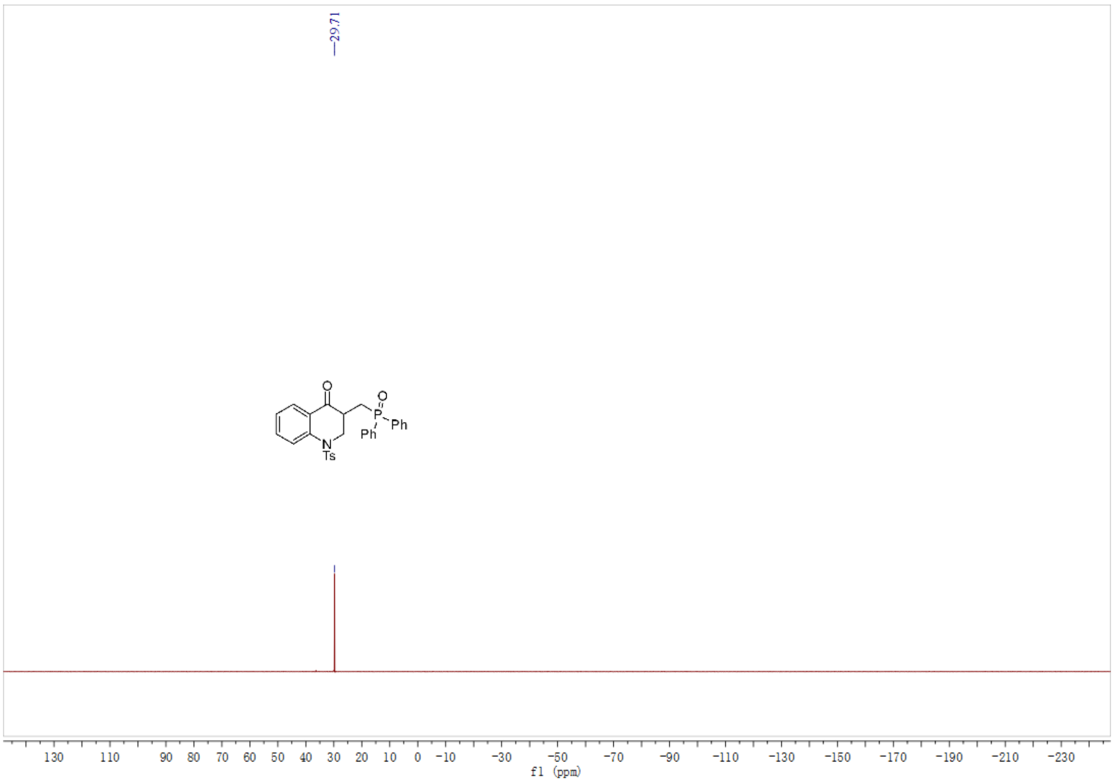
29.71
O
P
Ph
Ph
N
Ts
f1 (ppm)

Compound 3lf ^{1}H NMR, ^{13}C NMR and ^{31}P NMR

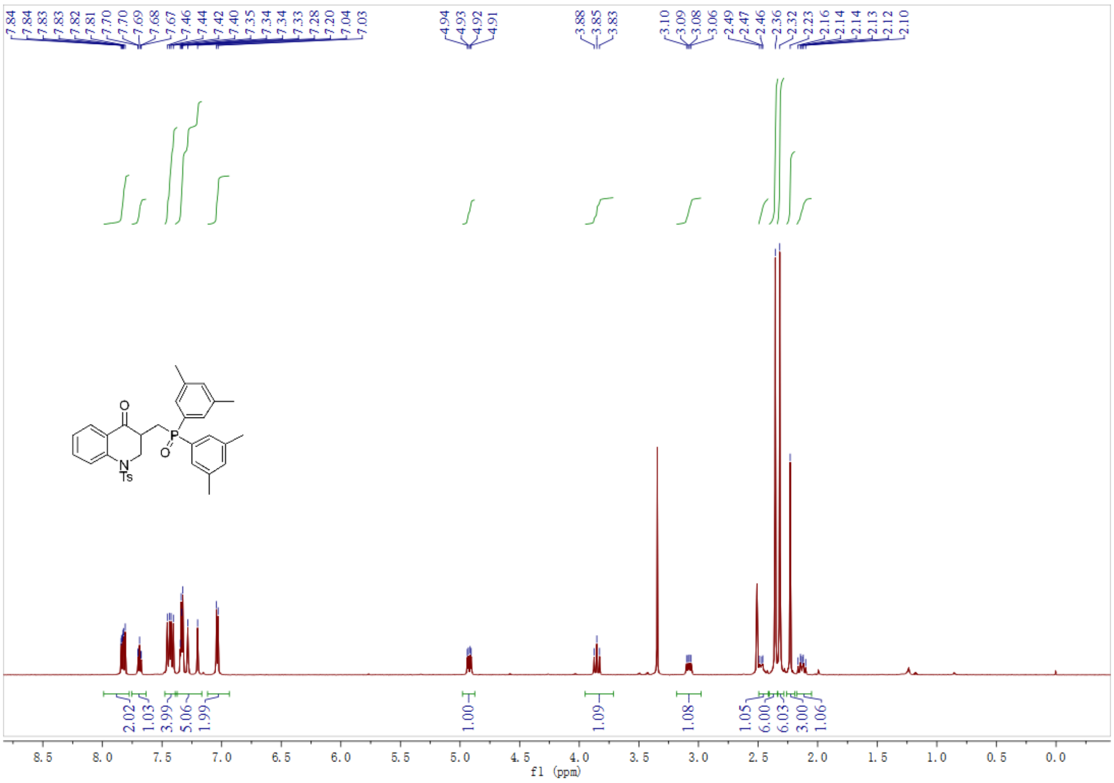

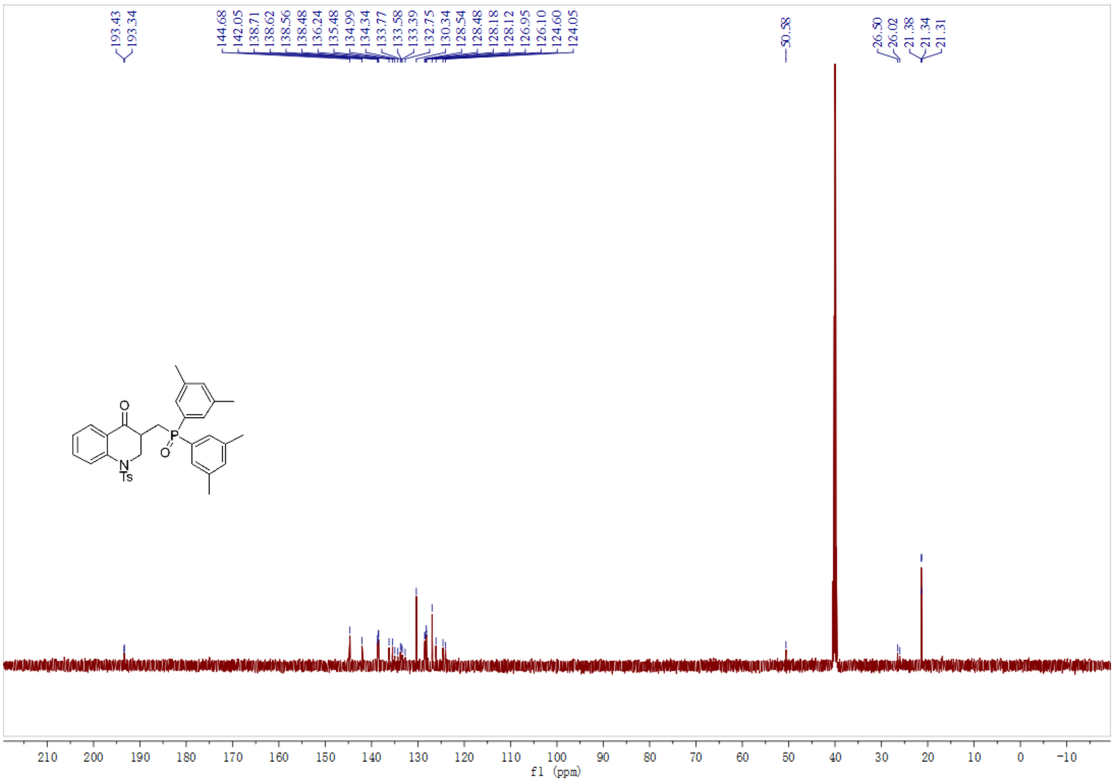

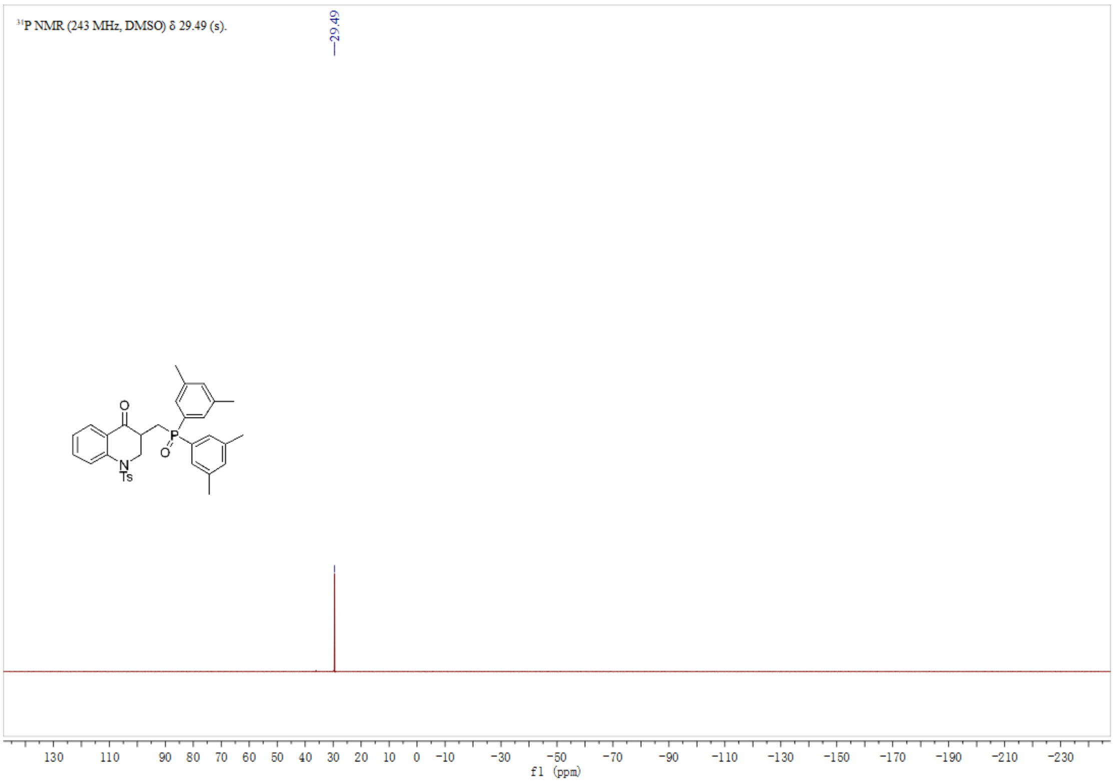
31P NMR (243 MHz, DMSO) δ 29.49 (s).
29.49
O
P
O
N
Ts
130 110 90 80 70 60 50 40 30 20 10 0 −10 −30 −50 −70 −90 −110 −130 −150 −170 −190 −210 −230
f1 (ppm)

Compound 3ab ^{1}H NMR, ^{13}C NMR and ^{31}P NMR

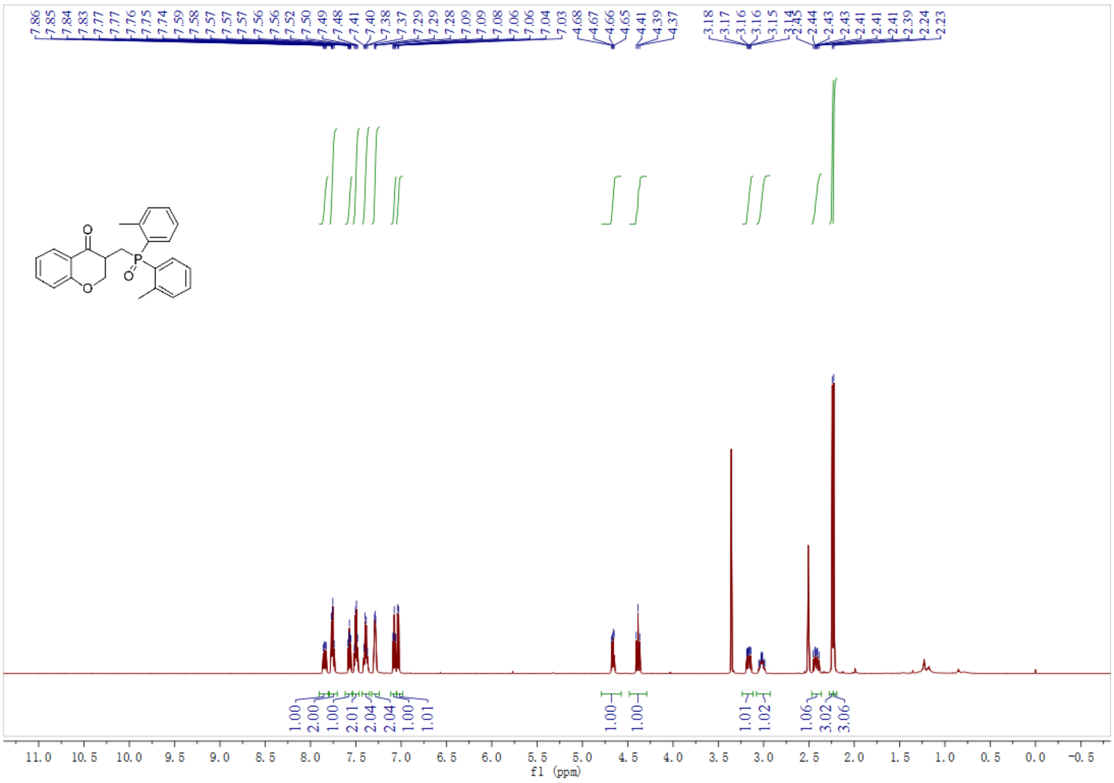

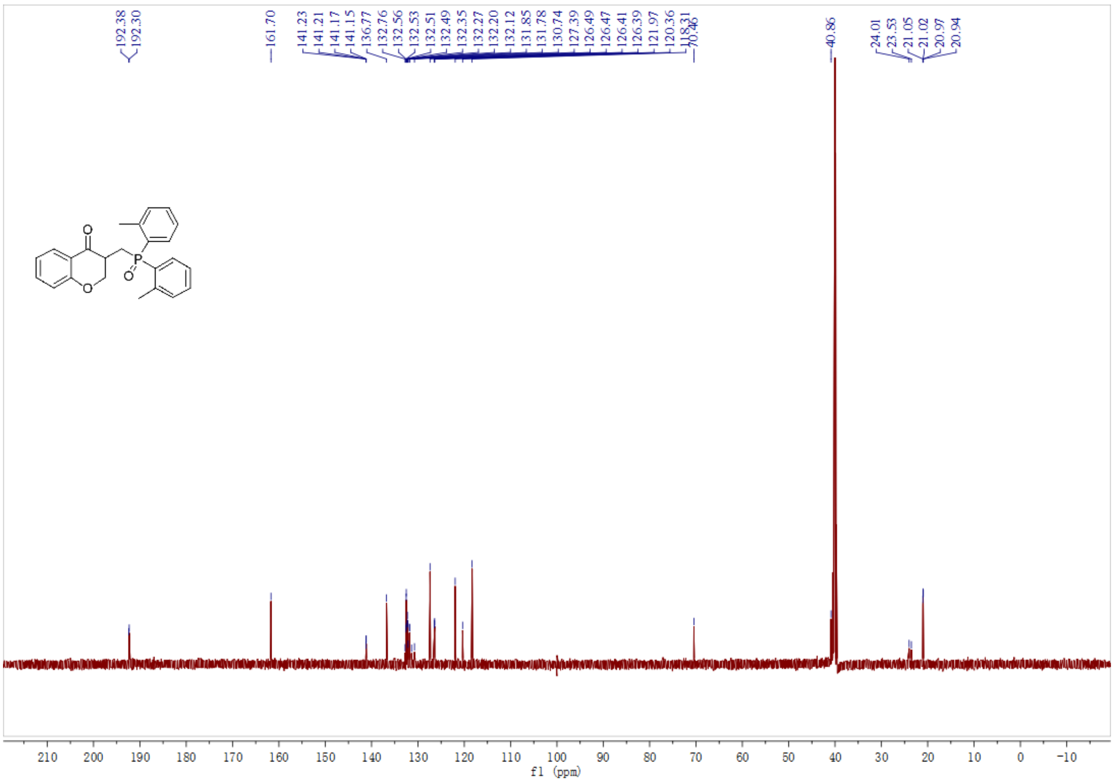

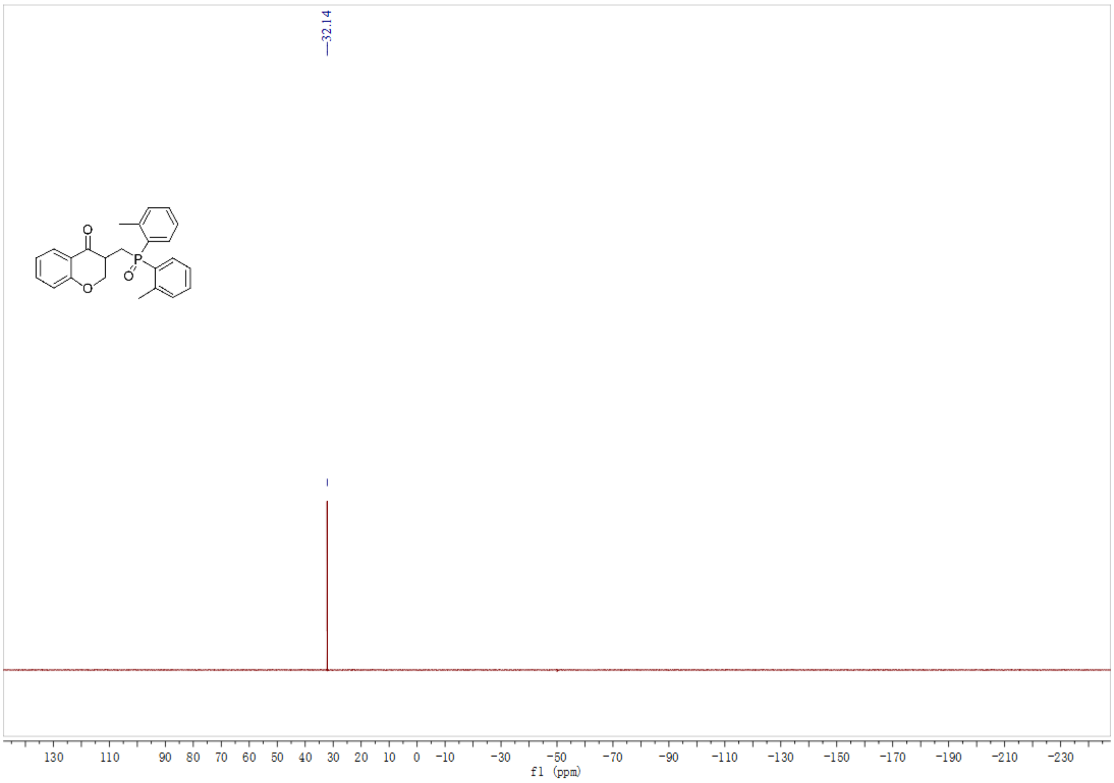
32.14
130
110
90
80
70
60
50
40
30
20
10
0
−10
−30
−50
−70
−90
−110
−130
−150
−170
−190
−210
−230
f1 (ppm)

Compound 3ac ^{1}H NMR, ^{13}C NMR and ^{31}P NMR

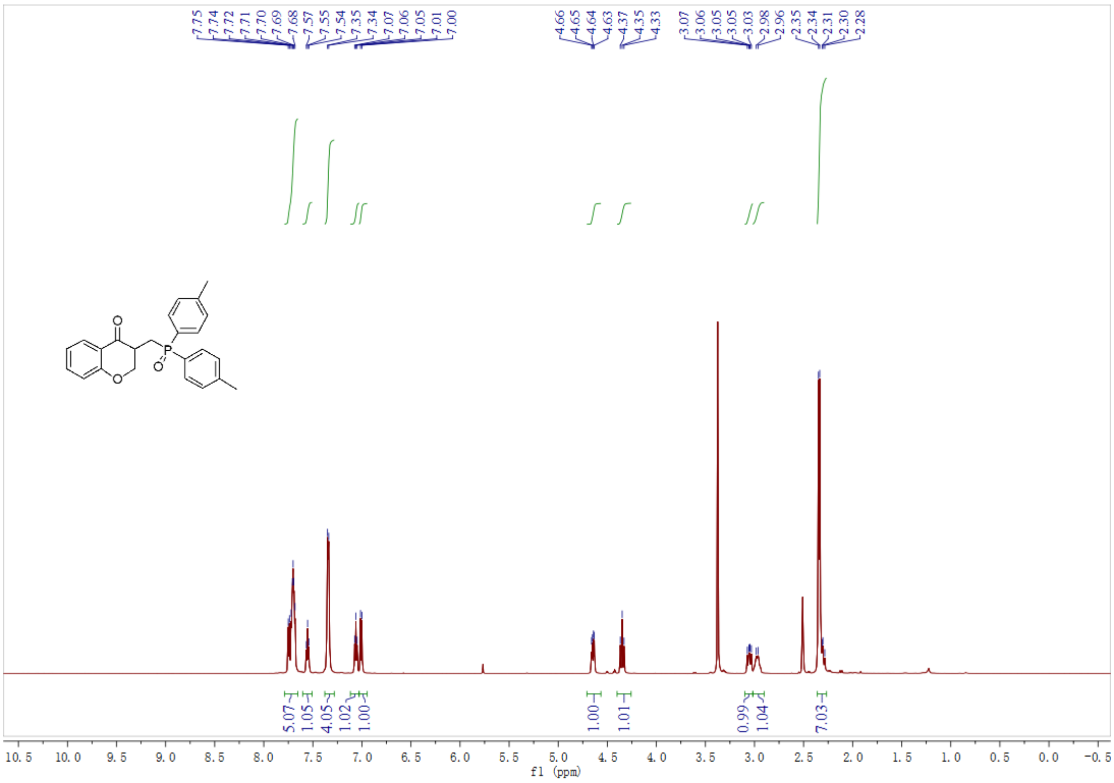

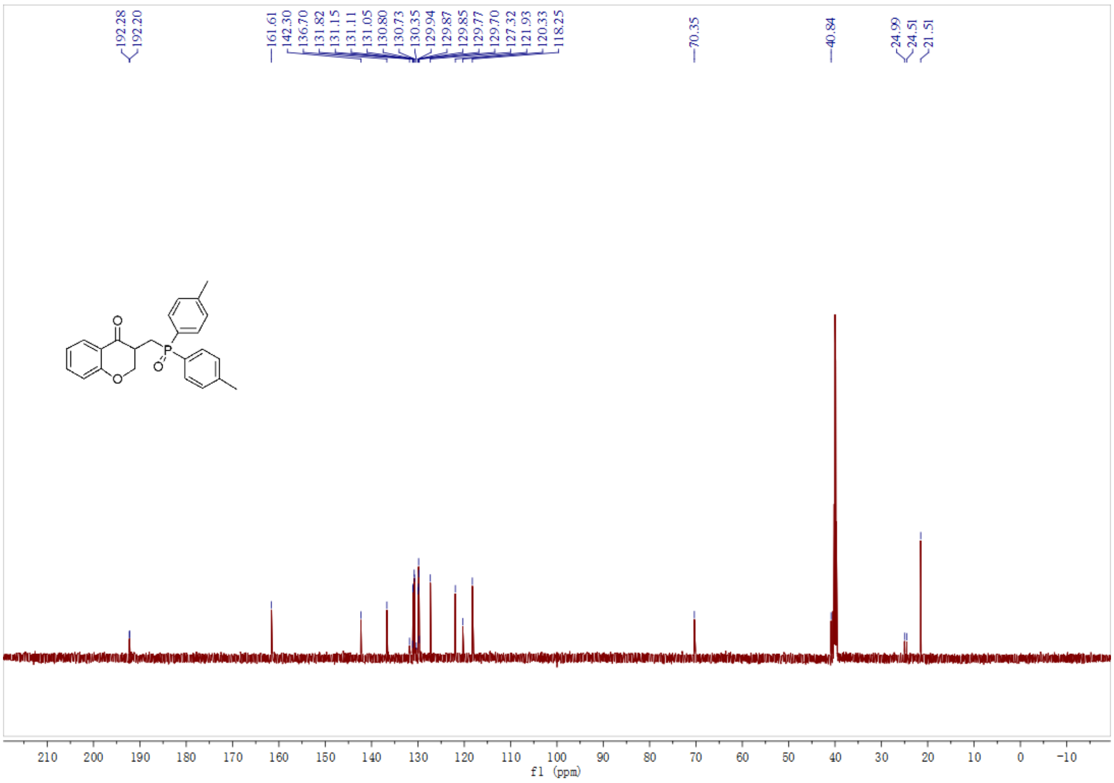

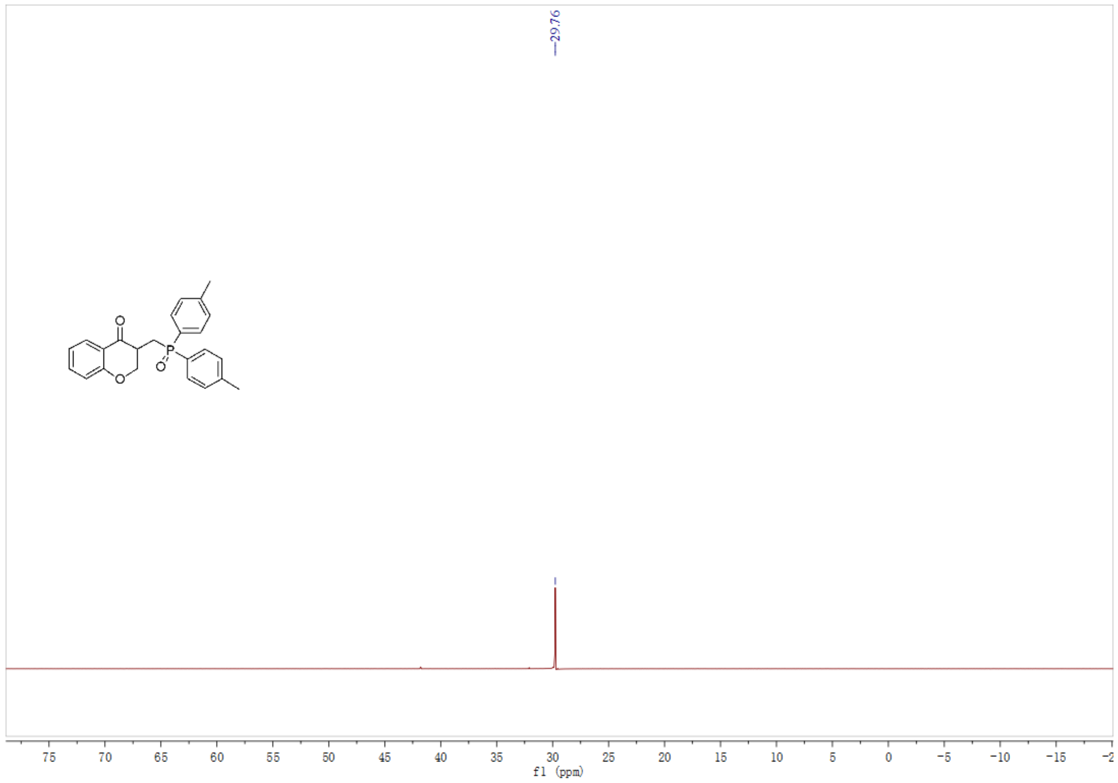
29.76
75
70
65
60
55
50
45
40
35
30
25
20
15
10
5
0
-5
-10
-15
f1 (ppm)

Compound 3ad ^{1}H NMR, ^{13}C NMR and ^{31}P NMR

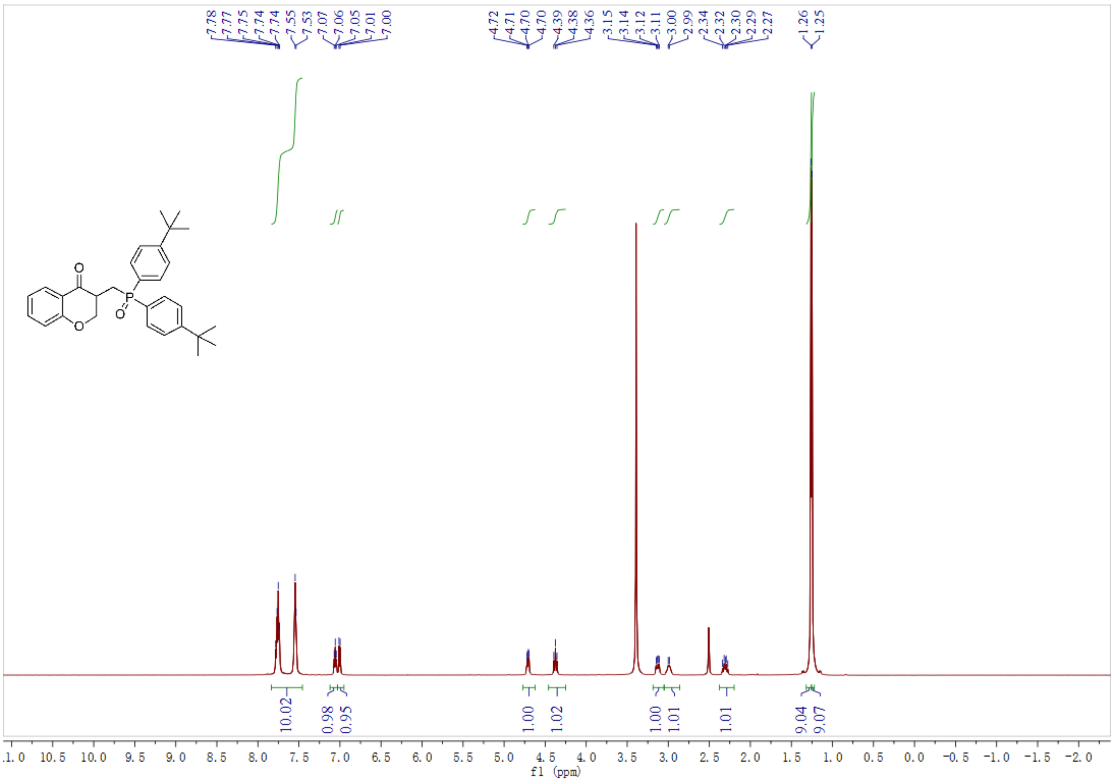

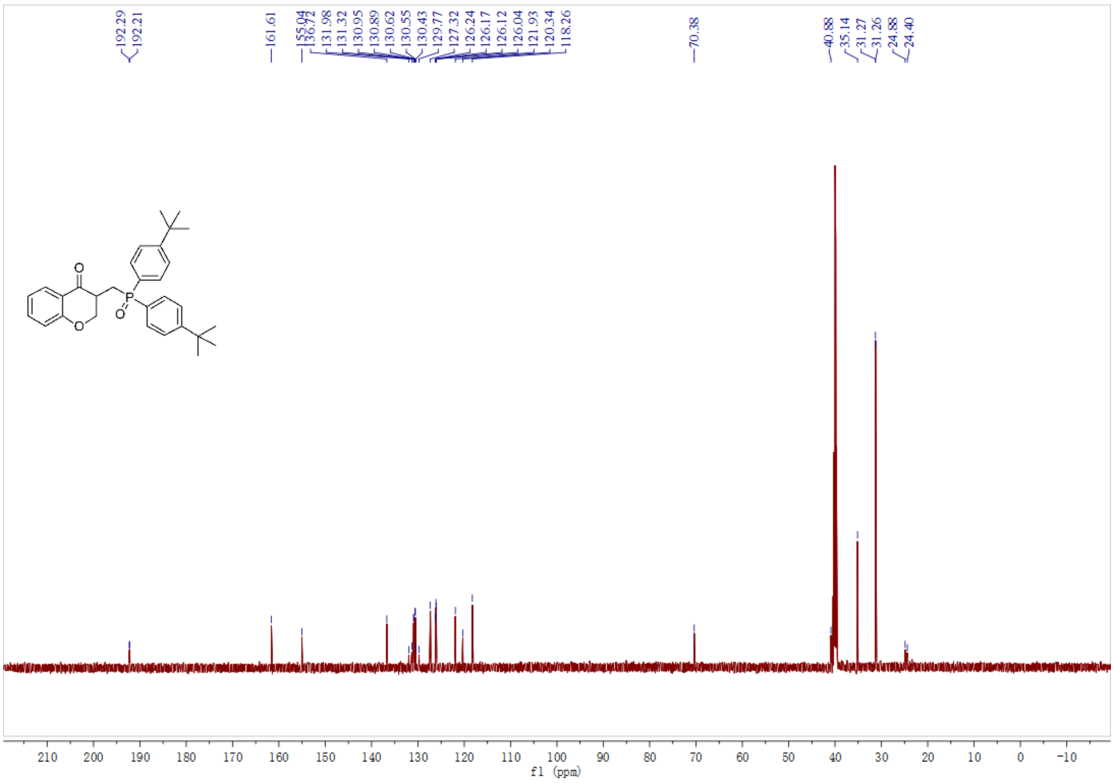

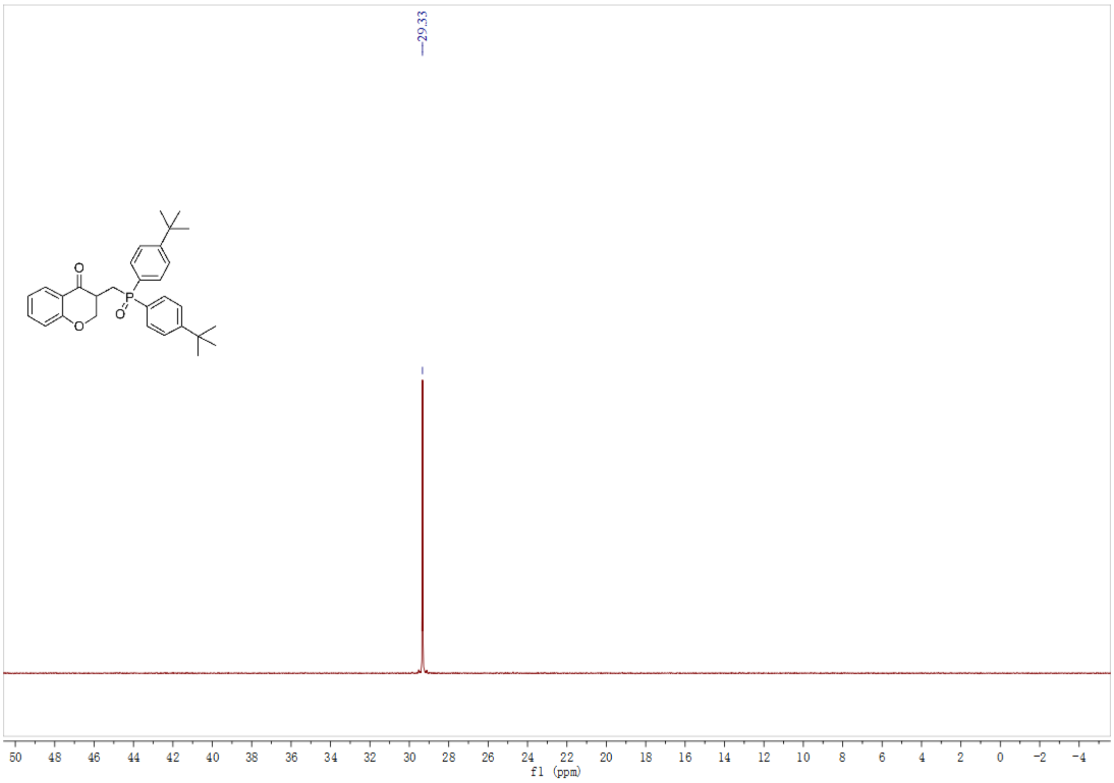

29.33
f1 (ppm)

Compound 3ae ^{1}H NMR, ^{13}C NMR and ^{31}P NMR

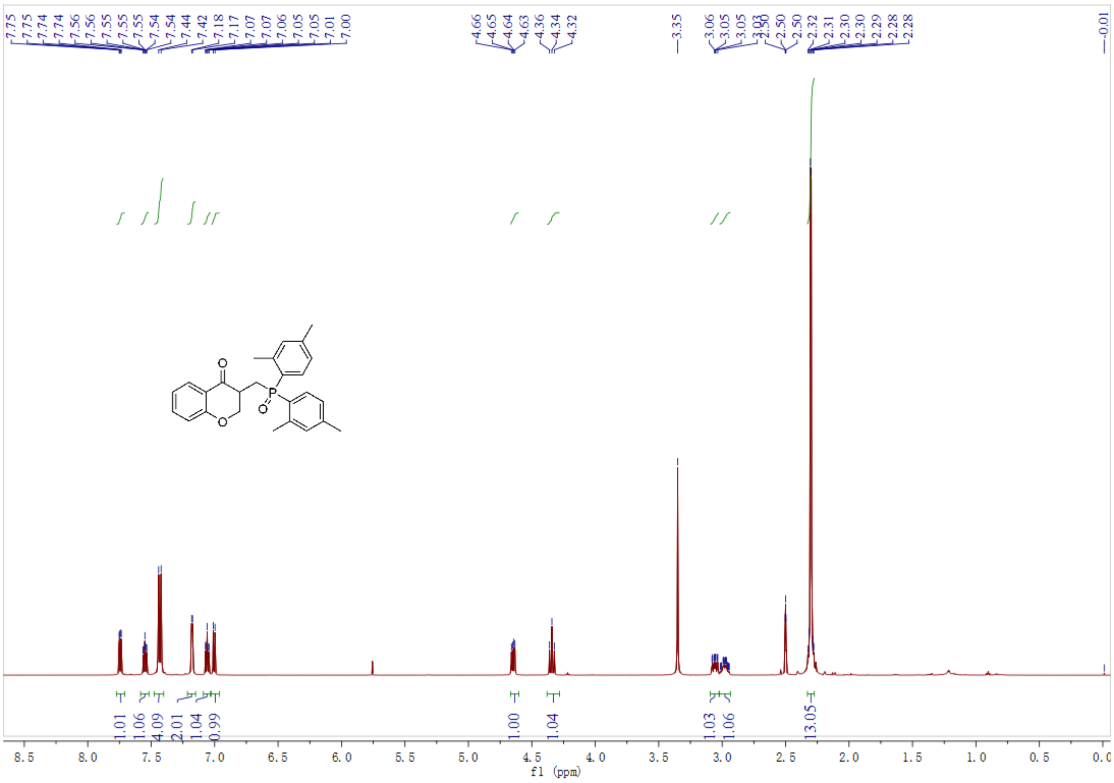

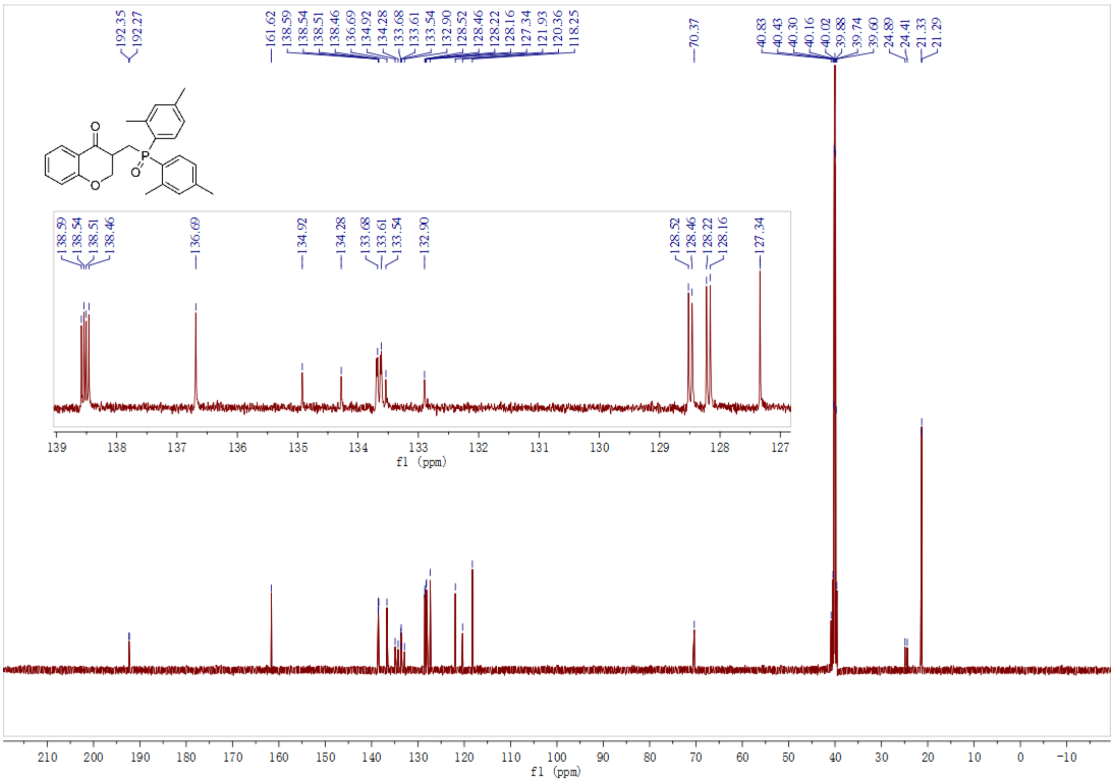

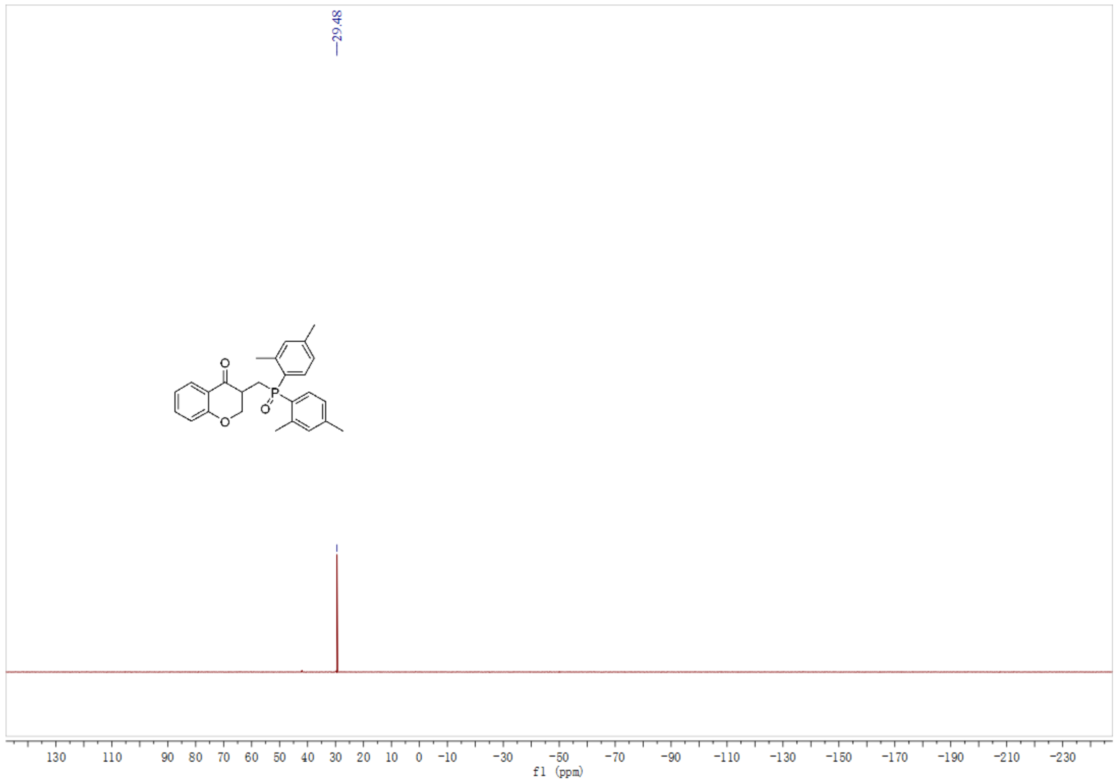

—29.48
130
110
90
80
70
60
50
40
30
20
10
0
-10
-30
-50
-70
-90
-110
-130
-150
-170
-190
-210
-230
f1 (ppm)

Compound 3af ^{1}H NMR, ^{13}C NMR and ^{31}P NMR

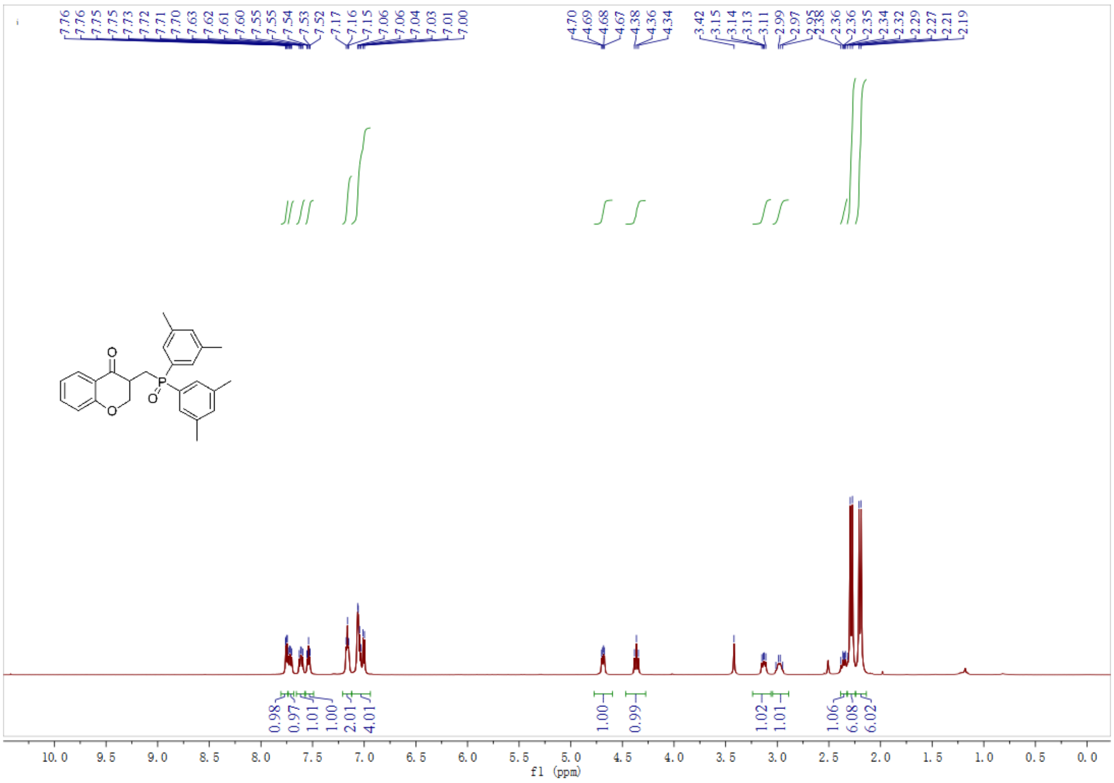

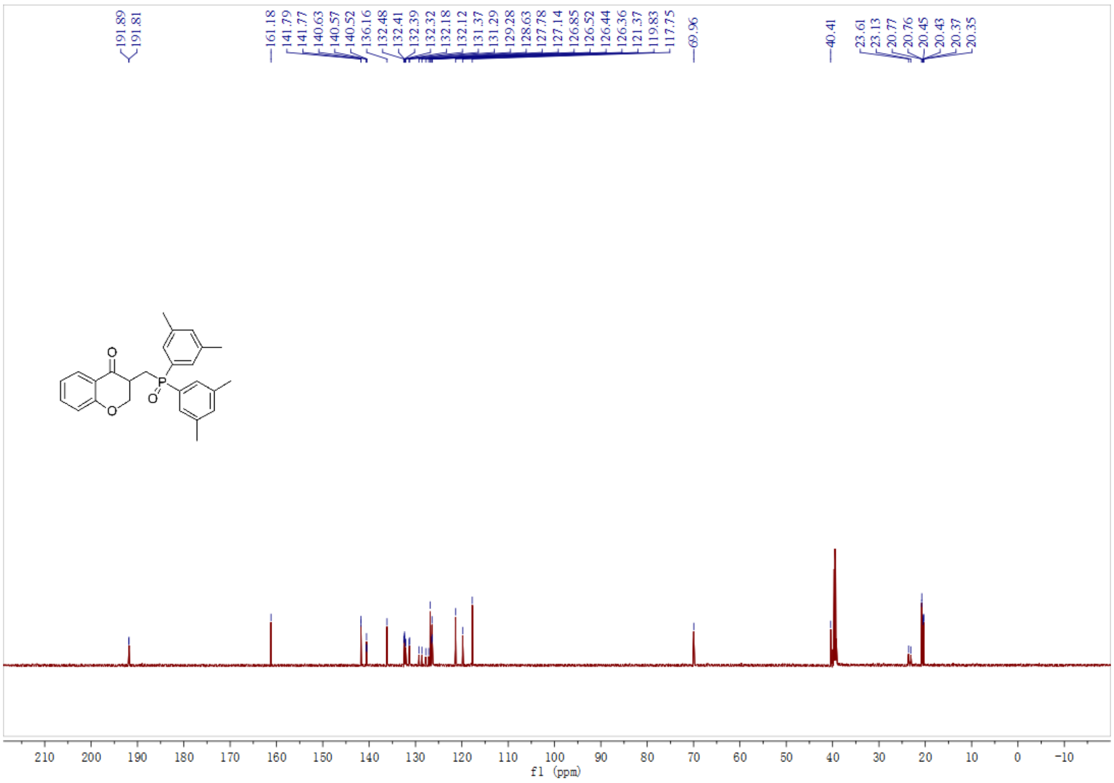

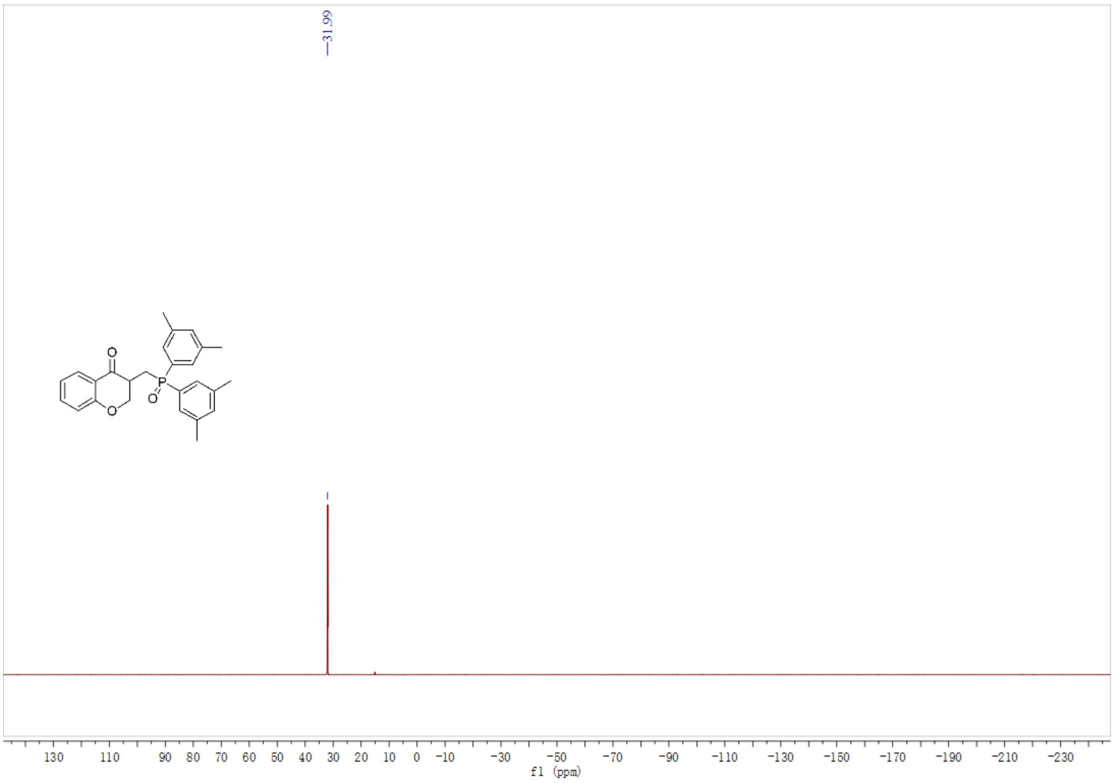

31.99
O
P
O
O
130
110
90
80
70
60
50
40
30
20
10
0
-10
-30
-50
-70
-90
-110
-130
-150
-170
-190
-210
-230
f1 (ppm)

Compound 3ag ^{1}H NMR, ^{13}C NMR, ^{19}F NMR and ^{31}P NMR

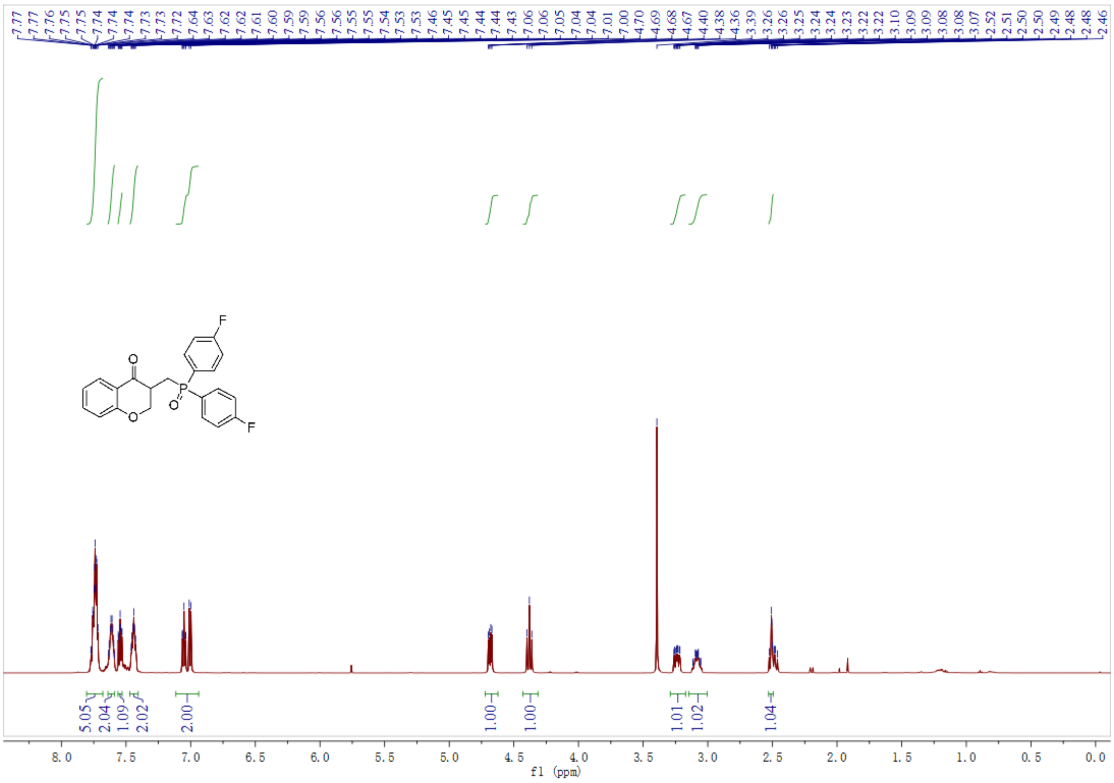

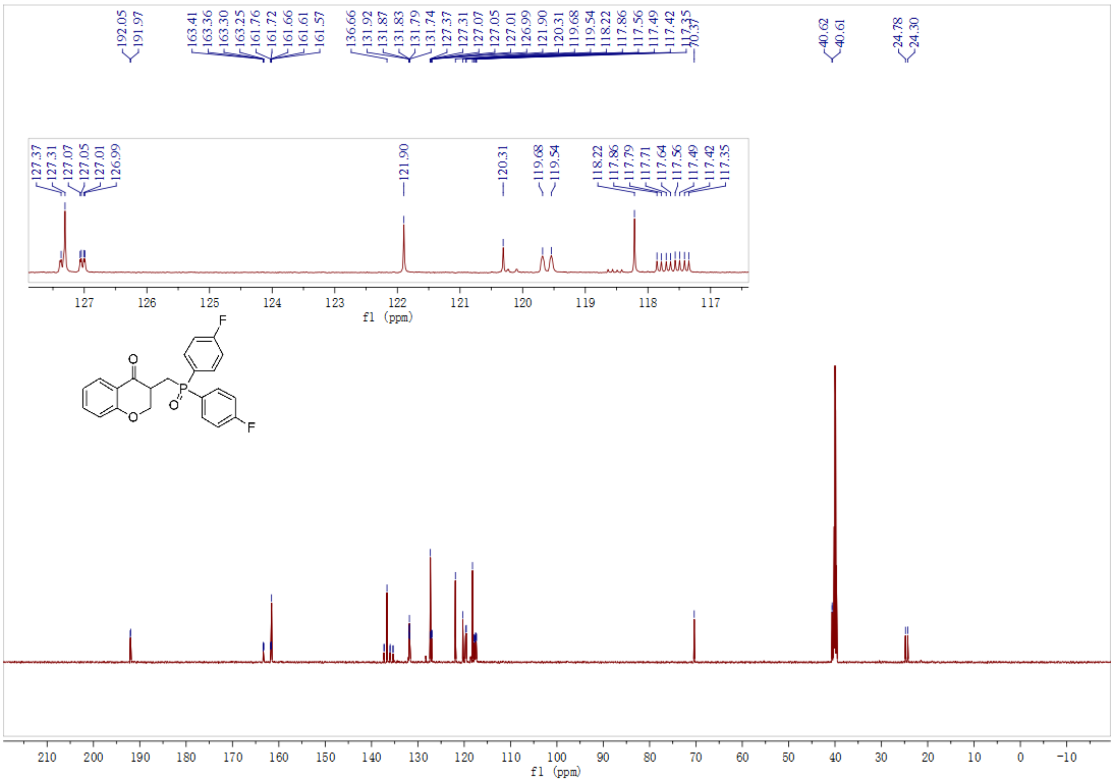

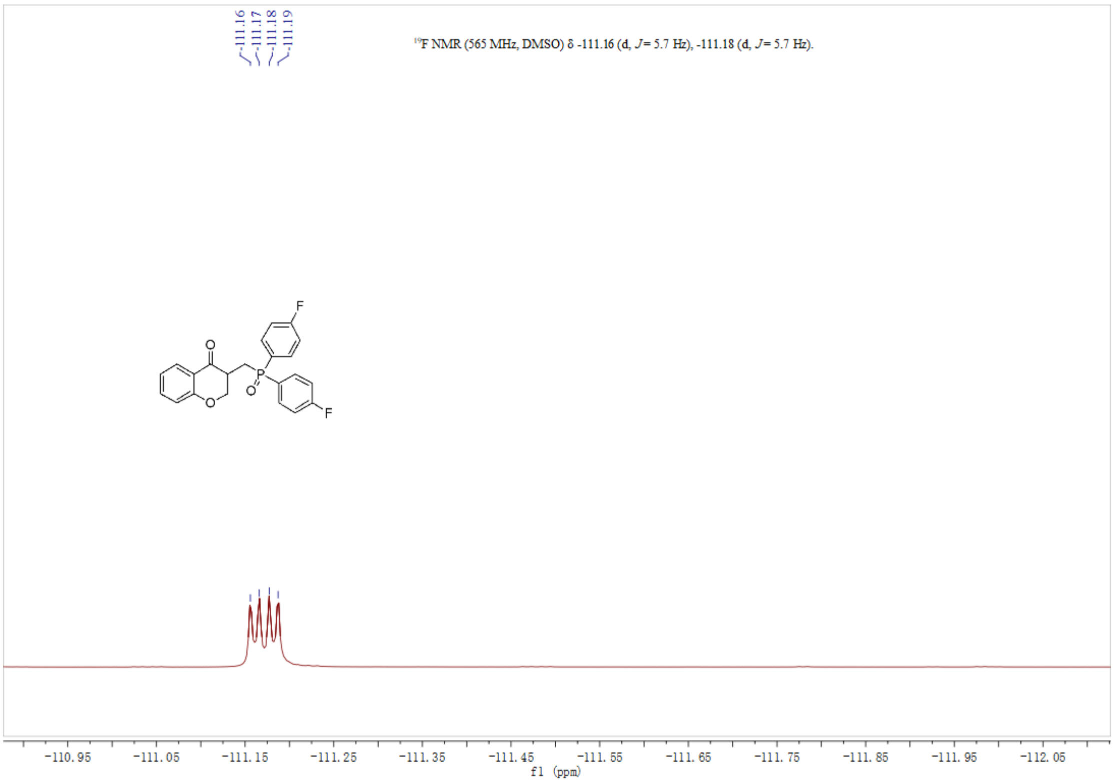

-111.16
-111.17
-111.18
-111.19
¹⁹F NMR (565 MHz, DMSO) δ -111.16 (d, J = 5.7 Hz), -111.18 (d, J = 5.7 Hz).
O
F
P
O
O
F
-110.95
-111.05
-111.15
-111.25
-111.35
-111.45
-111.55
-111.65
-111.75
-111.85
-111.95
-112.05
f1 (ppm)

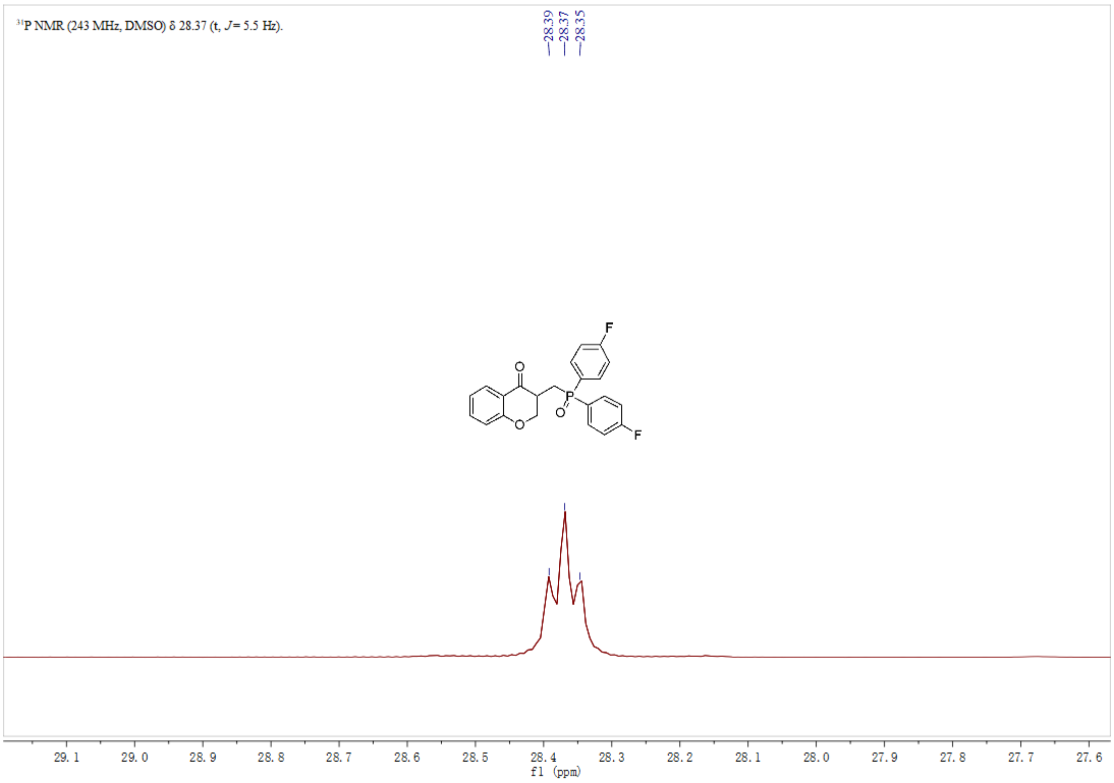

³¹P NMR (243 MHz, DMSO) δ 28.37 (t, J = 5.5 Hz).
28.39
28.37
28.35
O
F
P
O
O
F
29.1
29.0
28.9
28.8
28.7
28.6
28.5
28.4
28.3
28.2
28.1
28.0
27.9
27.8
27.7
27.6
f1 (ppm)

Compound 3ah ^{1}H NMR, ^{13}C NMR and ^{31}P NMR

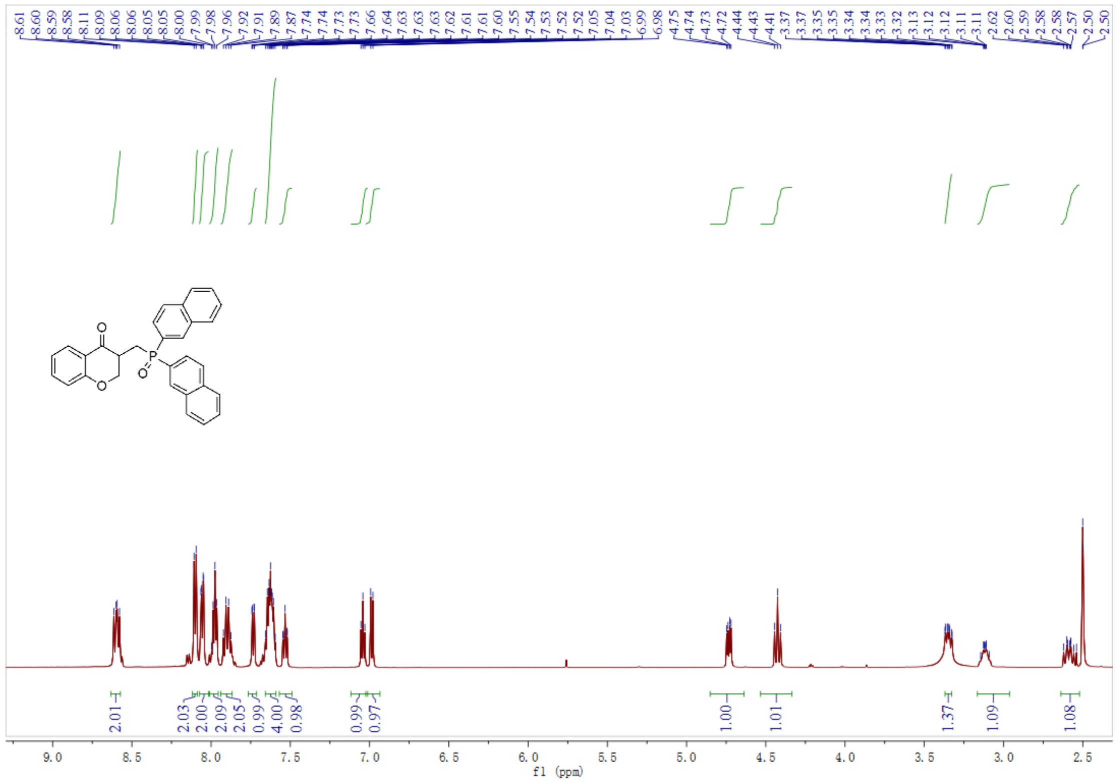

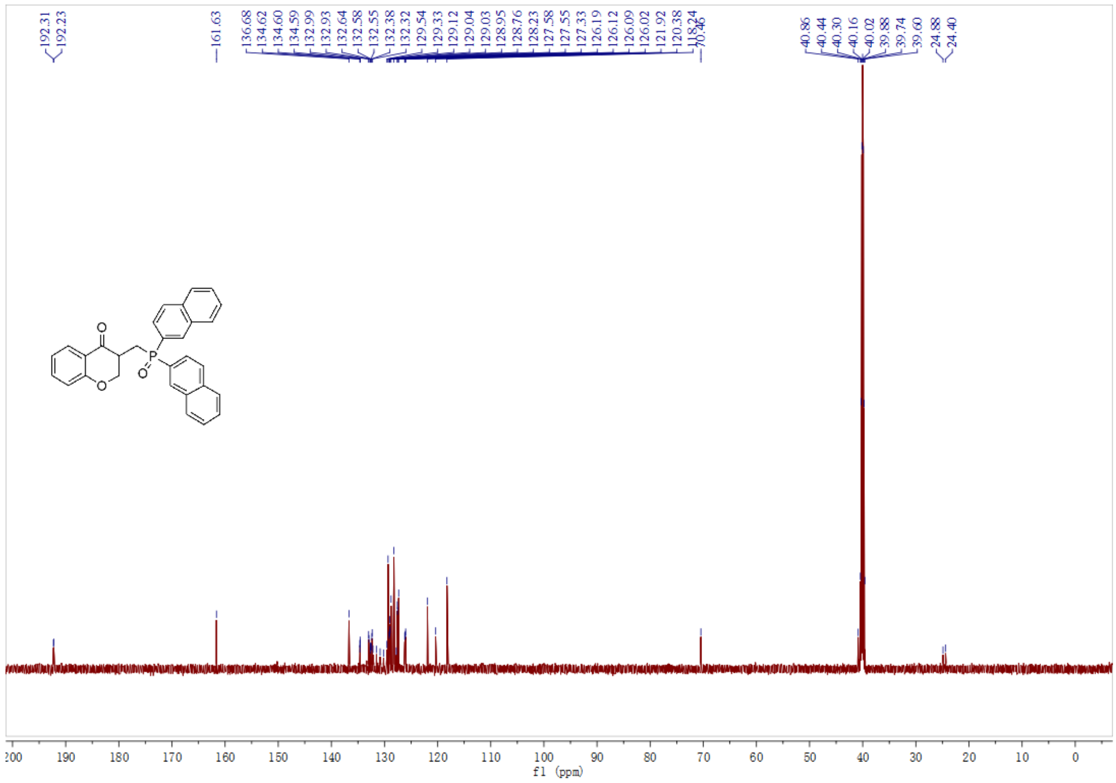

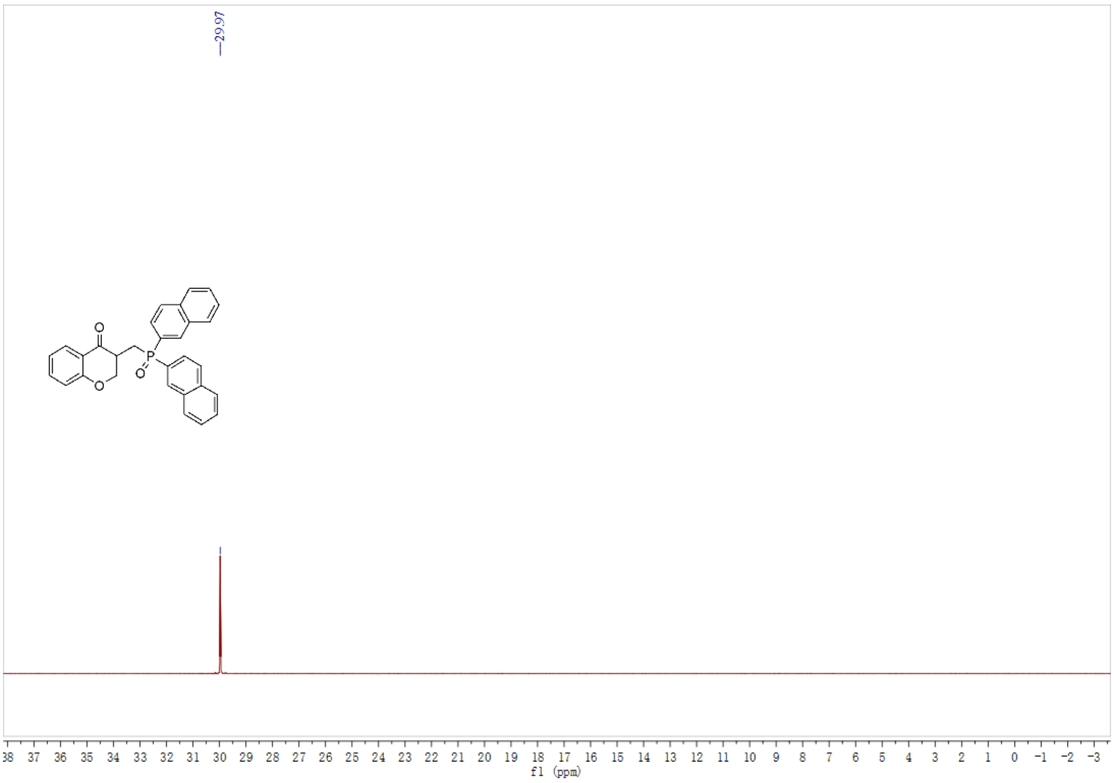
29.97
38 37 36 35 34 33 32 31 30 29 28 27 26 25 24 23 22 21 20 19 18 17 16 15 14 13 12 11 10 9 8 7 6 5 4 3 2 1 0 -1 -2 -3
f1 (ppm)

Compound 3ai ^{1}H NMR, ^{13}C NMR and ^{31}P NMR

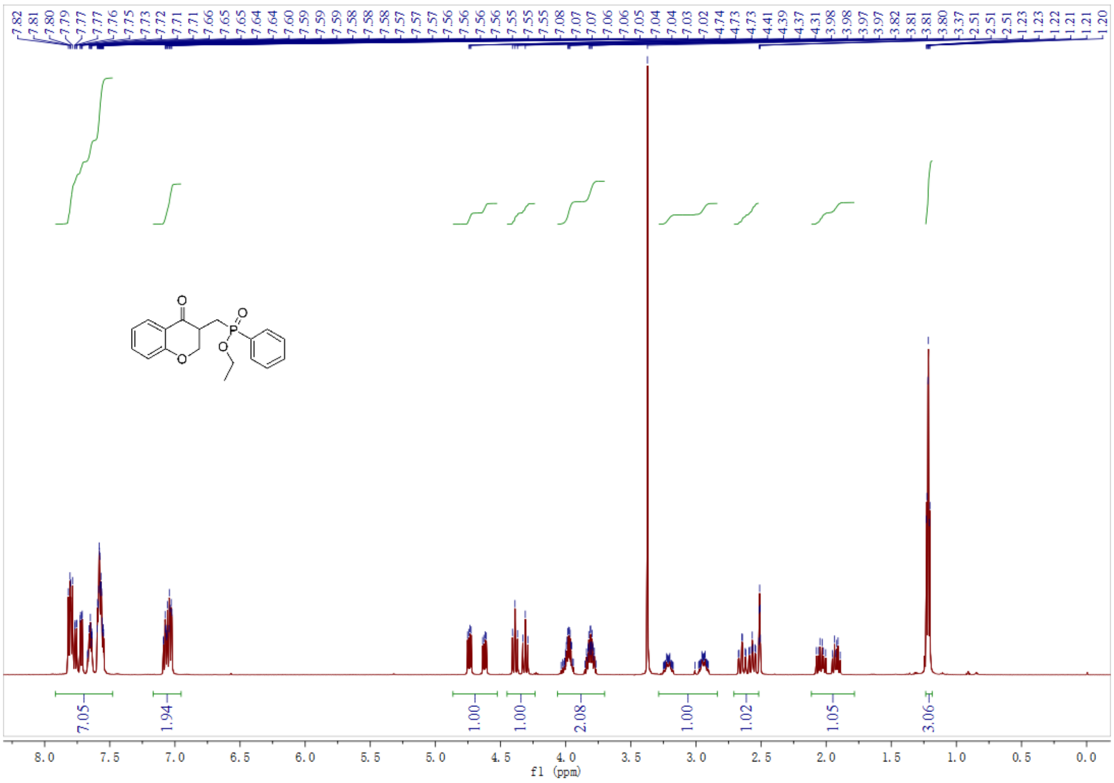

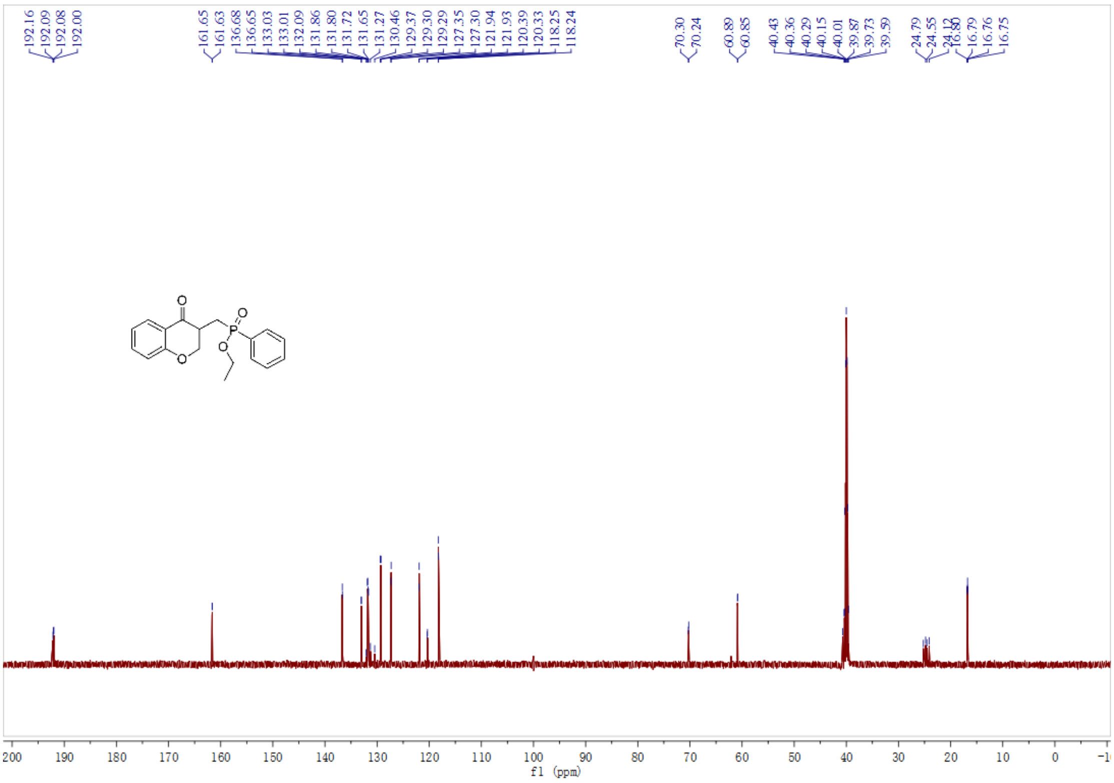

192.16
192.09
192.08
192.00
161.65
161.63
136.68
136.65
133.03
133.01
132.09
131.86
131.80
131.72
131.65
131.27
130.46
129.37
129.30
129.29
127.35
127.30
121.94
121.93
120.39
120.33
118.25
118.24
70.30
70.24
60.89
60.85
40.43
40.36
40.29
40.15
40.01
39.87
39.73
39.59
24.79
24.55
24.12
16.80
16.79
16.76
16.75
O
O
P
O
O
f1 (ppm)

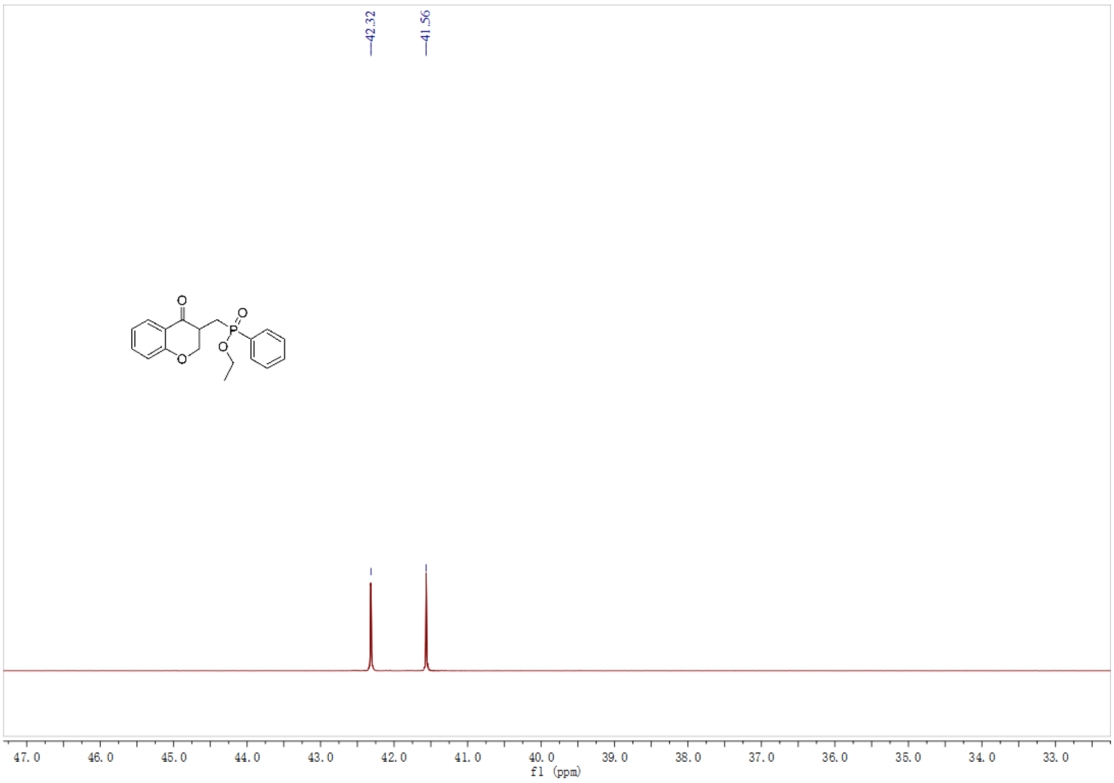

42.32
41.56
O
O
P
O
O
f1 (ppm)

Compound 3aj ^{1}H NMR, ^{13}C NMR and ^{31}P NMR

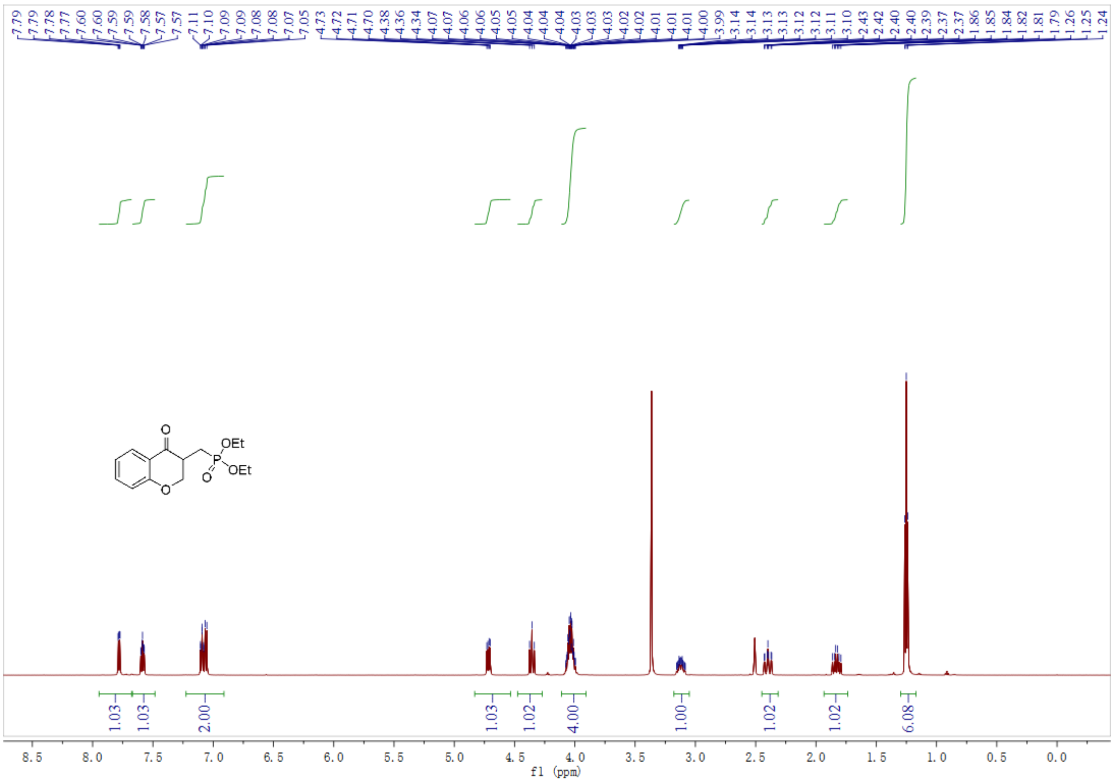

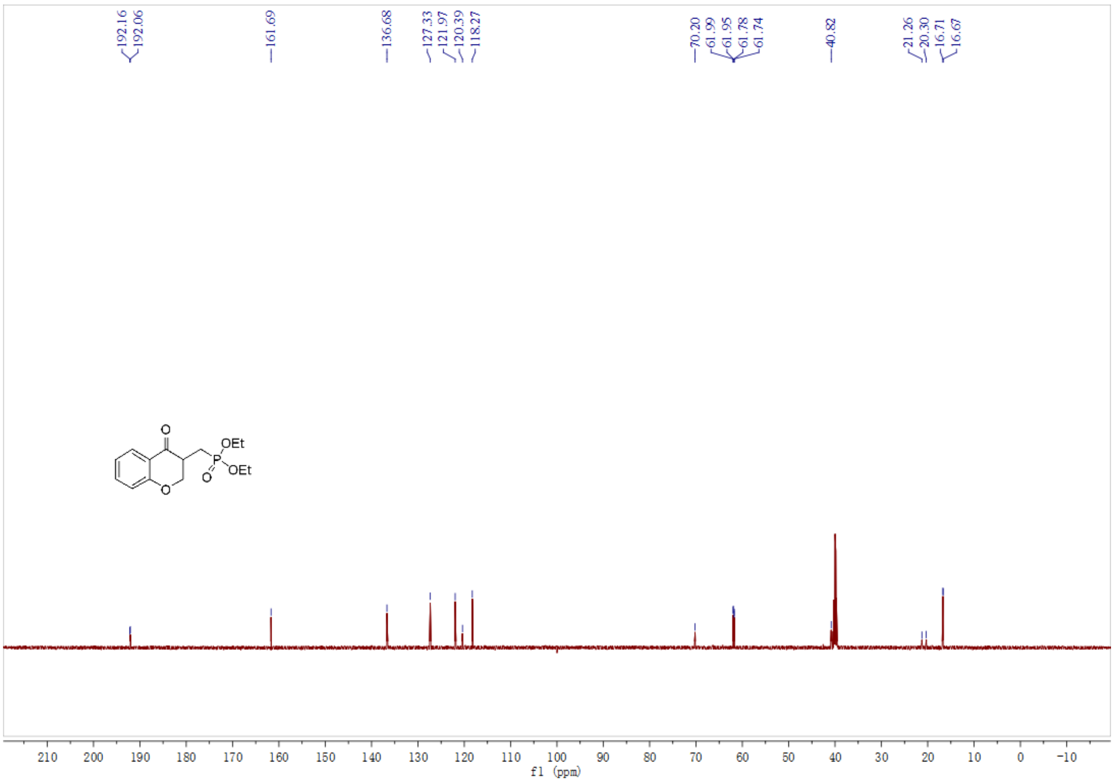

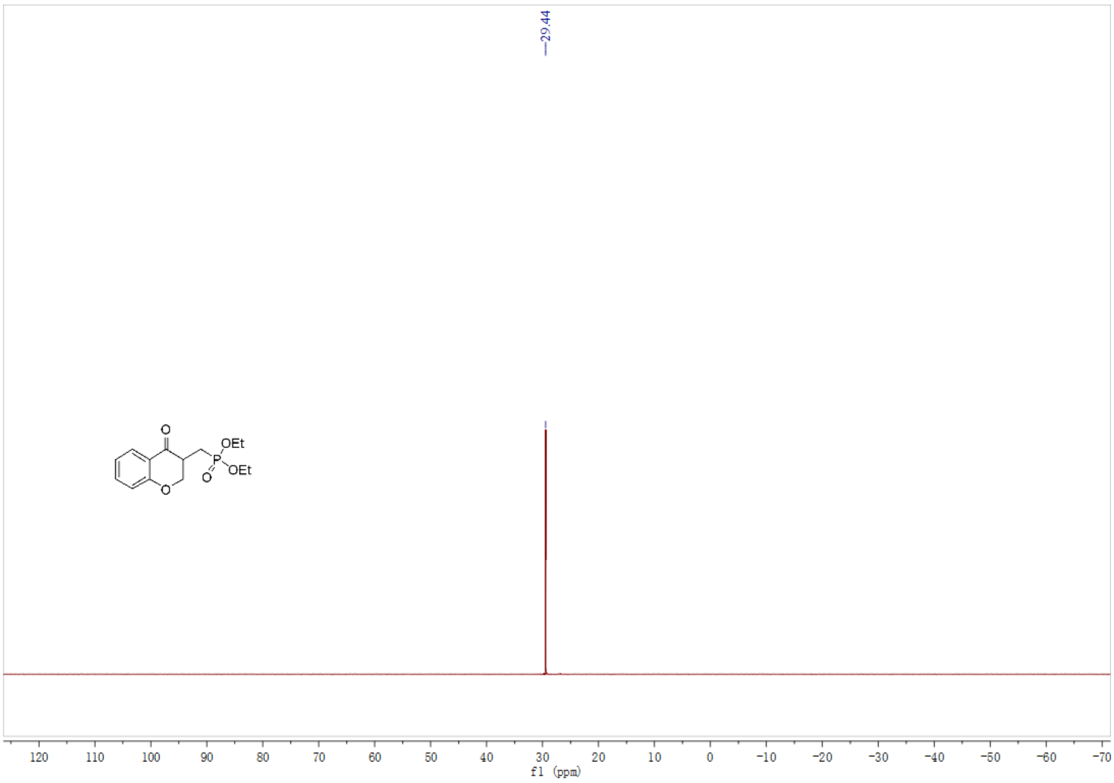

29.44
OEt
OEt
O
O
O
P
120 110 100 90 80 70 60 50 40 30 20 10 0 -10 -20 -30 -40 -50 -60 -70
f1 (ppm)

Compound 4 ^{1}H NMR, ^{13}C NMR, ^{13}C dept135 NMR and ^{31}P NMR

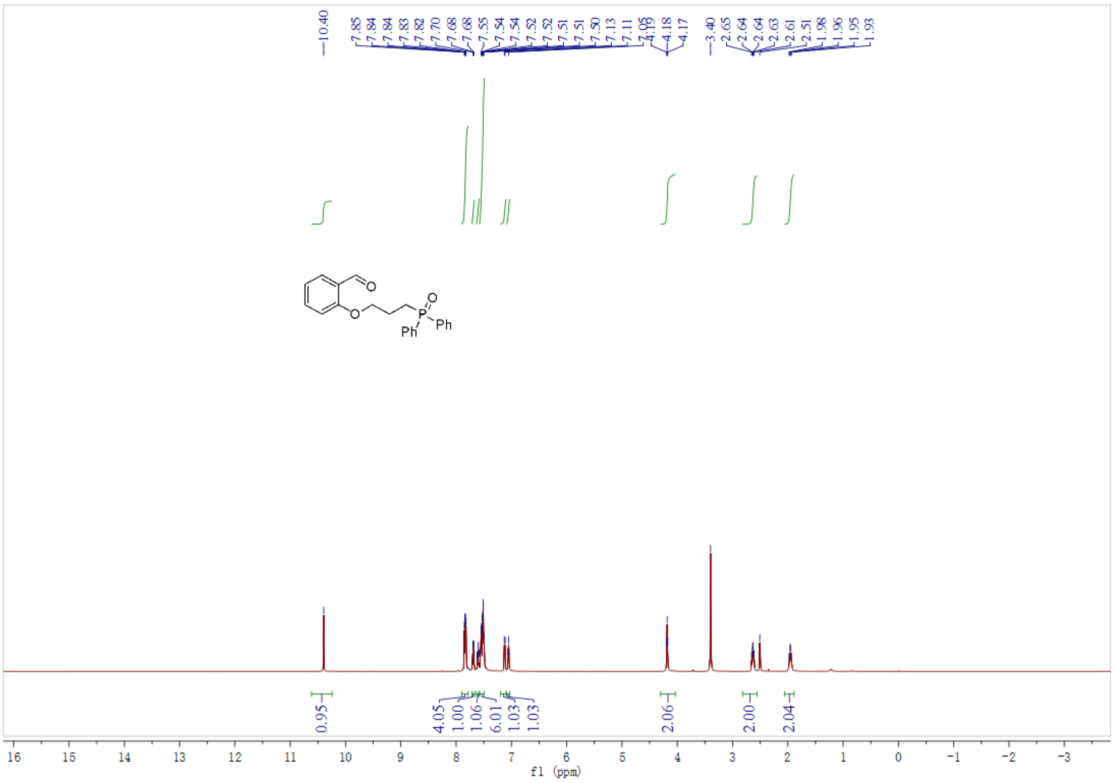

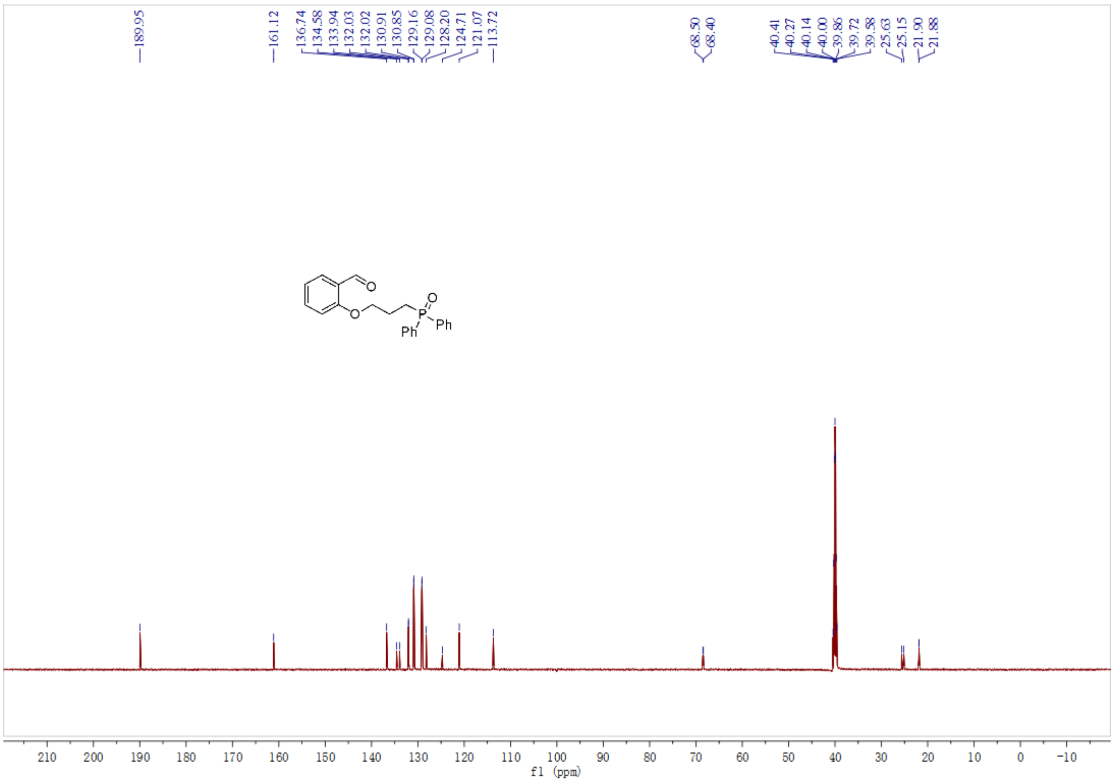

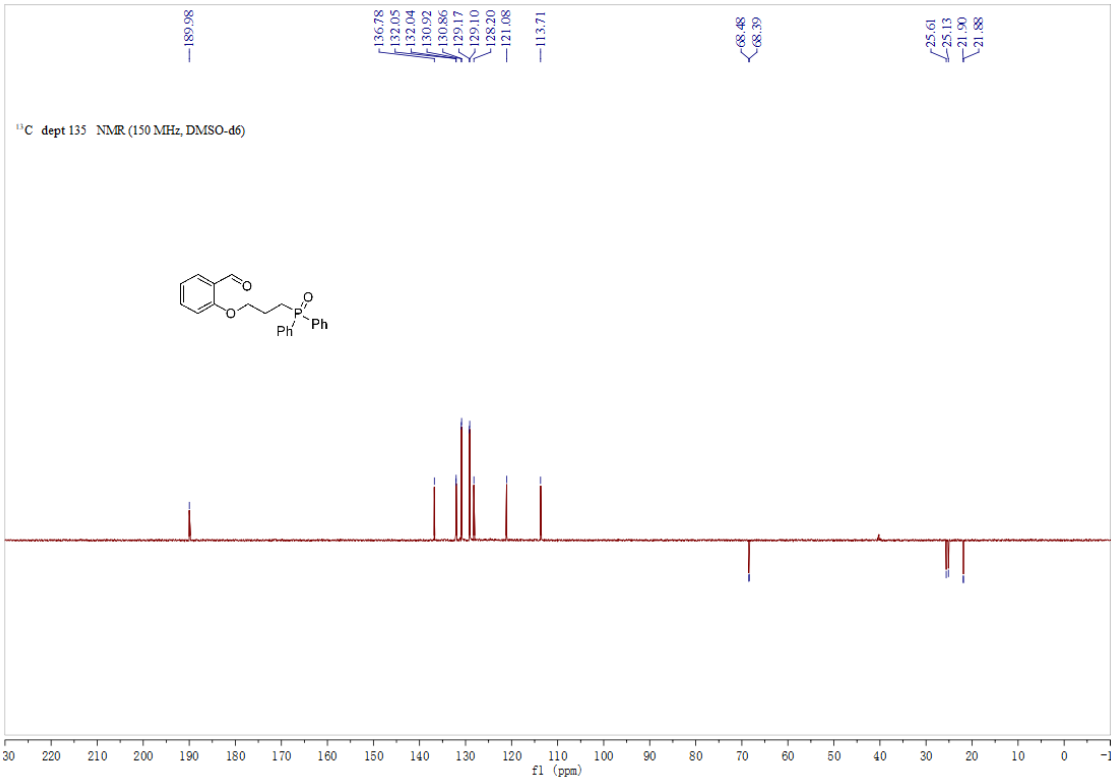
189.98
136.78
132.05
132.04
130.92
130.86
129.17
129.10
128.20
121.08
113.71
68.48
68.39
25.61
25.13
21.90
21.88
13C dept 135 NMR (150 MHz, DMSO-d6)
O
O
P
O
Ph
Ph
f1 (ppm)

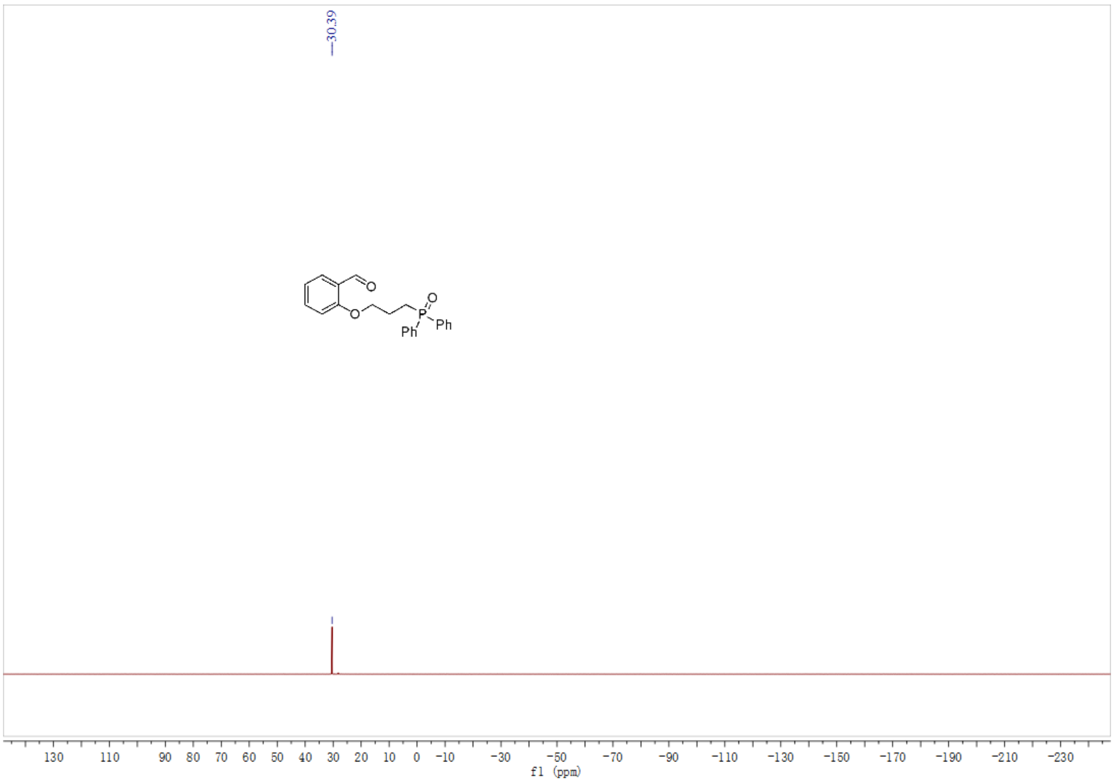
30.39
O
O
P
O
Ph
Ph
f1 (ppm)

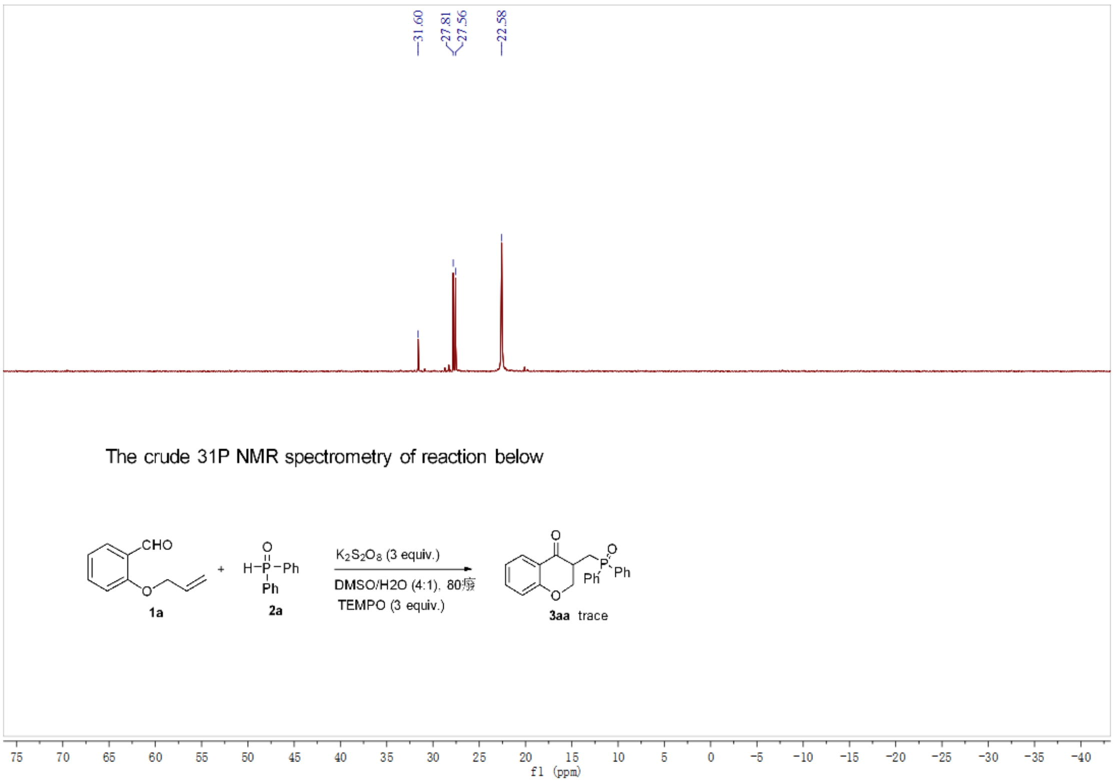

31.60
27.81
27.56
22.58
The crude 31P NMR spectrometry of reaction below
CHO
1a
2a
$K_2S_2O_8$ (3 equiv.)
DMSO/H2O (4:1), 80
TEMPO (3 equiv.)
3aa trace
f1 (ppm)

7 X-ray crystallography data for 3aa and 3ba

Compound **3aa**:

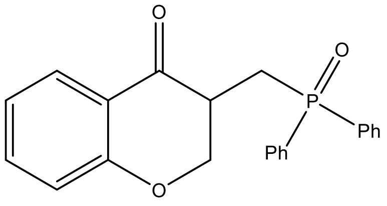

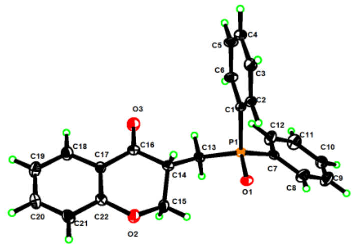

3aa CCDC 2002878

Table 1 Crystal data and structure refinement for 3aa.

Identification code	3aa
Empirical formula	$C_{22}H_{19}O_3P$
Formula weight	362.34
Temperature/K	170.0
Crystal system	orthorhombic
Space group	$P2_12_12_1$
a/Å	8.4345(8)
b/Å	13.1057(12)
c/Å	16.7585(17)
α/°	90
β/°	90
γ/°	90
Volume/Å^3	1852.5(3)
Z	4
ρ_{calc}g/cm^3	1.299
μ/mm^{-1}	0.167
F(000)	760.0
Crystal size/mm^3	0.16 × 0.12 × 0.08
Radiation	MoKα (λ = 0.71073)
2Θ range for data collection/°	3.946 to 53.484
Index ranges	$-8 \leq h \leq 10$, $-16 \leq k \leq 16$, $-18 \leq l \leq 20$
Reflections collected	13092
Independent reflections	3805 [R_{int} = 0.0776, R_{sigma} = 0.0911]
Data/restraints/parameters	3805/0/236
Goodness-of-fit on F^2	1.191
Final R indexes [I>=2σ (I)]	R_1 = 0.0700, wR_2 = 0.1100
Final R indexes [all data]	R_1 = 0.1115, wR_2 = 0.1240
Largest diff. peak/hole / e Å^{-3}	0.45/-0.37
Flack parameter	0.5(2)

Table 2 Fractional Atomic Coordinates (×10^4) and Equivalent Isotropic Displacement Parameters (Å^2×10^3) for 3aa. U_{eq} is defined as 1/3 of of the trace of the orthogonalised U_{IJ} tensor.

Atom	*x*	*y*	*z*	U(eq)
P1	8431.5(16)	4870.9(10)	5549.6(9)	22.4(3)
O1	9480(4)	4154(3)	5113(2)	26.4(9)
O2	10597(5)	6176(3)	3183(2)	38.3(11)
O3	6971(5)	7652(3)	4322(3)	45.3(12)
C14	8759(6)	6239(4)	4313(3)	23.3(13)
C17	8598(7)	7504(4)	3183(3)	24.9(13)
C1	6369(6)	4556(3)	5397(3)	21.2(12)
C2	6044(6)	3653(4)	5003(3)	25.1(13)
C7	8839(6)	4871(4)	6602(3)	24.1(12)
C16	8024(6)	7195(4)	3973(4)	27.4(14)
C6	5126(6)	5168(4)	5646(3)	30.3(13)
C22	9844(7)	6973(4)	2816(3)	28.6(14)
C13	8642(6)	6172(4)	5225(3)	25.0(13)
C4	3250(7)	3984(4)	5095(4)	31.6(14)
C3	4486(7)	3373(4)	4847(4)	30.6(14)
C15	10461(7)	6162(4)	4037(3)	34.2(15)
C18	7904(7)	8310(4)	2770(4)	37.3(16)
C8	9832(8)	4128(5)	6900(4)	42.5(17)
C12	8239(8)	5585(5)	7125(4)	42.6(17)
C21	10345(8)	7227(5)	2051(3)	37.0(16)
C20	9634(8)	8028(5)	1665(4)	38.9(16)
C5	3574(6)	4879(4)	5494(4)	34.0(13)
C10	9654(7)	4830(5)	8211(4)	40.4(16)
C19	8419(9)	8575(4)	2022(4)	41.9(17)
C11	8646(9)	5570(5)	7927(4)	47.6(18)
C9	10234(8)	4107(5)	7702(4)	47.4(18)

Table 3 Anisotropic Displacement Parameters (Å^2×10^3) for 3aa. The Anisotropic displacement factor exponent takes the form: $-2\pi^2[h^2a^{*2}U_{11}+2hka^*b^*U_{12}+\ldots]$.

Atom	U_{11}	U_{22}	U_{33}	U_{23}	U_{13}	U_{12}
P1	16.7(6)	22.3(7)	28.3(8)	-0.6(6)	0.1(7)	-0.2(6)
O1	22(2)	27(2)	30(2)	-3.8(17)	3.3(18)	4.7(16)
O2	44(3)	40(3)	32(3)	5(2)	10(2)	19(2)
O3	46(3)	47(3)	42(3)	6(2)	15(2)	22(2)
C14	24(3)	21(3)	25(3)	-6(2)	3(3)	1(2)

C17	27(3)	21(3)	26(3)	1(2)	-1(3)	-3(2)
C1	16(3)	22(3)	25(3)	1(2)	-1(2)	-2(2)
C2	21(3)	22(3)	32(4)	2(2)	0(3)	2(2)
C7	20(3)	24(3)	29(3)	0(2)	2(2)	-5(2)
C16	21(3)	29(3)	32(4)	-4(3)	1(3)	0(2)
C6	21(3)	27(3)	43(4)	-6(3)	3(3)	3(2)
C22	30(3)	28(3)	28(3)	1(3)	3(3)	-3(3)
C13	21(3)	26(3)	29(3)	1(2)	1(3)	-6(2)
C4	21(3)	35(3)	39(4)	6(3)	-5(3)	-6(3)
C3	31(3)	21(3)	39(4)	-2(3)	-3(3)	-6(2)
C15	39(4)	36(3)	28(4)	7(3)	7(3)	10(3)
C18	45(4)	33(3)	35(4)	3(3)	6(3)	11(3)
C8	41(4)	43(4)	44(5)	-2(3)	0(3)	15(3)
C12	50(4)	45(4)	33(4)	3(3)	-5(4)	13(3)
C21	44(4)	39(4)	29(4)	-2(3)	12(3)	3(3)
C20	52(4)	38(4)	27(4)	7(3)	1(3)	-5(3)
C5	19(3)	39(3)	44(4)	-2(3)	8(3)	6(3)
C10	36(3)	59(4)	26(4)	8(3)	-8(3)	-4(4)
C19	53(4)	34(3)	39(4)	8(3)	-5(4)	8(4)
C11	62(5)	55(4)	26(4)	-10(3)	-2(4)	8(4)
C9	48(4)	60(4)	34(4)	6(3)	-6(4)	20(4)

Table 4 Bond Lengths for 3aa.

Atom	Atom	Length/Å	Atom	Atom	Length/Å
P1	O1	1.483(4)	C2	C3	1.390(7)
P1	C1	1.806(5)	C7	C8	1.379(8)
P1	C7	1.797(5)	C7	C12	1.378(8)
P1	C13	1.799(5)	C6	C5	1.386(7)
O2	C22	1.370(6)	C22	C21	1.389(8)
O2	C15	1.436(7)	C4	C3	1.379(8)
O3	C16	1.221(6)	C4	C5	1.377(7)
C14	C16	1.509(7)	C18	C19	1.371(8)
C14	C13	1.534(7)	C8	C9	1.387(9)
C14	C15	1.512(7)	C12	C11	1.388(8)
C17	C16	1.466(8)	C21	C20	1.371(8)
C17	C22	1.402(8)	C20	C19	1.386(9)
C17	C18	1.392(7)	C10	C11	1.374(8)
C1	C2	1.382(7)	C10	C9	1.366(9)
C1	C6	1.384(7)			

Table 5 Bond Angles for 3aa.

Atom	Atom	Atom	Angle/°	Atom	Atom	Atom	Angle/°
O1	P1	C1	111.1(2)	O3	C16	C17	122.5(5)
O1	P1	C7	111.7(2)	C17	C16	C14	115.8(5)
O1	P1	C13	113.1(2)	C1	C6	C5	120.2(5)
C7	P1	C1	108.8(2)	O2	C22	C17	122.0(5)
C7	P1	C13	106.1(2)	O2	C22	C21	117.1(5)
C13	P1	C1	105.6(2)	C21	C22	C17	120.9(5)
C22	O2	C15	114.8(4)	C14	C13	P1	111.2(3)
C16	C14	C13	113.4(4)	C5	C4	C3	119.4(5)
C16	C14	C15	109.3(4)	C4	C3	C2	120.3(5)
C15	C14	C13	111.2(4)	O2	C15	C14	112.4(5)
C22	C17	C16	120.4(5)	C19	C18	C17	120.9(6)
C18	C17	C16	121.4(5)	C7	C8	C9	120.9(6)
C18	C17	C22	118.2(5)	C7	C12	C11	121.0(6)
C2	C1	P1	117.0(4)	C20	C21	C22	119.0(6)
C2	C1	C6	119.3(5)	C21	C20	C19	121.0(6)
C6	C1	P1	123.7(4)	C4	C5	C6	120.6(5)
C1	C2	C3	120.3(5)	C9	C10	C11	119.7(6)
C8	C7	P1	118.1(4)	C18	C19	C20	119.9(6)
C12	C7	P1	123.6(4)	C10	C11	C12	119.9(6)
C12	C7	C8	118.2(5)	C10	C9	C8	120.3(6)
O3	C16	C14	121.6(5)				

Table 6 Hydrogen Atom Coordinates (Å×10^4) and Isotropic Displacement Parameters (Å^2×10^3) for 3aa.

Atom	*x*	*y*	*z*	U(eq)
H14	8171.58	5642.61	4086.63	28
H2	6889.8	3223.11	4838.69	30
H6	5337.46	5786.58	5920.83	36
H13A	7718.26	6574.26	5409.68	30
H13B	9607.39	6470.95	5466.49	30
H4	2184.71	3790.26	4991.48	38
H3	4271.57	2756.4	4569.09	37
H15A	11073.16	6738.4	4261.64	41
H15B	10928.17	5521.03	4243.6	41
H18	7063.22	8682.45	3009.42	45
H8	10247.66	3622.92	6551.27	51
H12	7536.05	6095.55	6932.95	51
H21	11169.05	6851.58	1799.98	44

H20	9979.08	8210.55	1144.64	47
H5	2726.43	5302.82	5666.33	41
H10	9945.13	4822.29	8758.98	48
H19	7944.11	9131.34	1748.54	50
H11	8228.89	6070.1	8279.78	57
H9	10916.15	3587.84	7898.83	57

Compound **3ba :**

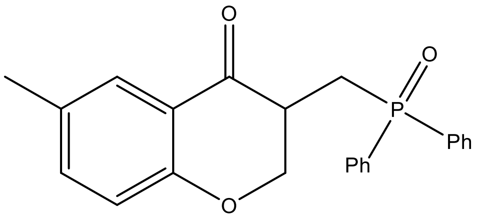

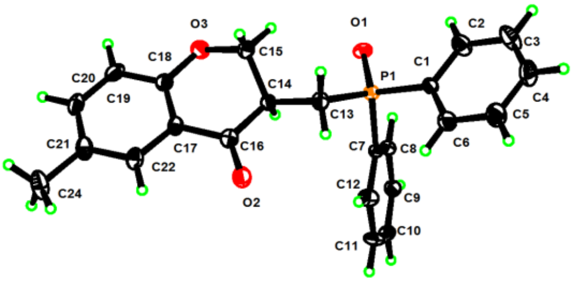

3ba CCDC 2002879

Table 1 Crystal data and structure refinement for 3ba.

Identification code	3ba
Empirical formula	$C_{23}H_{21}O_3P$
Formula weight	376.37
Temperature/K	170.0
Crystal system	monoclinic
Space group	$P2_1/c$
a/Å	14.3475(13)
b/Å	8.5440(7)
c/Å	17.3370(13)
α/°	90
β/°	113.471(3)
γ/°	90
Volume/Å^3	1949.4(3)
Z	4
ρ_{calc}g/cm^3	1.282
μ/mm^{-1}	0.161
F(000)	792.0
Crystal size/mm^3	0.15 × 0.12 × 0.08
Radiation	MoKα (λ = 0.71073)
2Θ range for data collection/°	4.816 to 52.774
Index ranges	$-17 \le h \le 17, -10 \le k \le 9, -21 \le l \le 18$

Reflections collected	13708
Independent reflections	3934 [R_{int} = 0.1109, R_{sigma} = 0.1304]
Data/restraints/parameters	3934/0/245
Goodness-of-fit on F^2	1.087
Final R indexes [I>=2σ (I)]	R_1 = 0.0839, wR_2 = 0.1179
Final R indexes [all data]	R_1 = 0.1692, wR_2 = 0.1462
Largest diff. peak/hole / e Å^{-3}	0.50/-0.45

Table 2 Fractional Atomic Coordinates (×10^4) and Equivalent Isotropic Displacement Parameters (Å^2×10^3) for 3ba. U_{eq} is defined as 1/3 of of the trace of the orthogonalised U_{IJ} tensor.

Atom	*x*	*y*	*z*	U(eq)
P1	7972.3(8)	4173.5(13)	3471.8(7)	26.3(3)
O1	8574.2(19)	3192(3)	4213.2(16)	32.3(7)
O2	4793(2)	5311(4)	2869.9(18)	43.4(8)
O3	5921(2)	2110(4)	4786.8(18)	43.5(8)
C7	8087(3)	6233(5)	3730(2)	23.5(9)
C8	8931(3)	6708(5)	4429(2)	30.4(10)
C14	6211(3)	3763(5)	3747(2)	26.4(10)
C13	6629(3)	3754(5)	3050(2)	30.4(10)
C18	4975(3)	2735(5)	4577(3)	33.1(11)
C17	4557(3)	3826(5)	3928(2)	28.6(10)
C1	8372(3)	3950(5)	2614(2)	27.0(10)
C16	5136(3)	4379(5)	3448(3)	32.4(11)
C10	8403(3)	9376(5)	4179(3)	34.1(11)
C22	3584(3)	4402(6)	3764(2)	35.9(11)
C9	9081(3)	8264(5)	4650(3)	32.6(11)
C11	7554(3)	8921(5)	3489(3)	37.6(12)
C6	7896(3)	4692(6)	1850(3)	43.0(12)
C21	3027(3)	3927(6)	4212(3)	38.9(12)
C15	6277(3)	2144(5)	4123(3)	38.8(12)
C12	7390(3)	7358(5)	3263(3)	35.9(11)
C19	4430(4)	2262(5)	5042(3)	41.5(12)
C2	9219(3)	3034(6)	2746(3)	43.9(13)
C20	3475(3)	2850(5)	4856(3)	40.5(12)
C5	8246(4)	4510(6)	1219(3)	52.0(14)
C4	9088(4)	3608(6)	1354(3)	54.7(15)
C3	9575(4)	2866(7)	2111(3)	60.2(16)
C24	1963(3)	4554(7)	3997(3)	61.6(17)

Table 3 Anisotropic Displacement Parameters ($Å^2×10^3$) for 3ba. The Anisotropic displacement factor exponent takes the form: $-2\pi^2[h^2a^{*2}U_{11}+2hka^*b^*U_{12}+…]$.

Atom	U_{11}	U_{22}	U_{33}	U_{23}	U_{13}	U_{12}
P1	27.6(6)	23.2(6)	28.3(6)	0.0(5)	11.4(5)	0.3(5)
O1	33.4(16)	26.7(18)	32.0(16)	5.6(13)	7.9(13)	2.6(13)
O2	35.1(17)	57(2)	39.4(18)	17.8(17)	16.7(15)	9.9(16)
O3	51(2)	45(2)	43.3(19)	15.6(16)	27.5(16)	14.8(16)
C7	28(2)	19(2)	26(2)	1.1(17)	14.4(19)	2.4(18)
C8	27(2)	28(3)	36(3)	6(2)	13(2)	6(2)
C14	27(2)	24(2)	30(2)	-3.3(19)	14.1(18)	-2.5(18)
C13	31(2)	31(3)	30(2)	-7.6(19)	13.3(19)	-5(2)
C18	37(3)	28(3)	37(3)	-4(2)	18(2)	-3(2)
C17	29(2)	34(3)	26(2)	-4(2)	13.7(18)	-5(2)
C1	25(2)	27(3)	31(2)	-2(2)	13.1(18)	1.9(19)
C16	33(2)	35(3)	29(2)	-6(2)	11(2)	-5(2)
C10	43(3)	21(3)	42(3)	-1(2)	21(2)	-2(2)
C22	34(2)	49(3)	25(2)	-3(2)	12.4(19)	-7(2)
C9	30(2)	29(3)	38(3)	-5(2)	12(2)	-4(2)
C11	47(3)	24(3)	38(3)	3(2)	13(2)	14(2)
C6	50(3)	44(3)	43(3)	7(2)	27(2)	10(2)
C21	33(2)	54(3)	32(2)	-10(2)	16(2)	-10(2)
C15	47(3)	36(3)	44(3)	7(2)	29(2)	4(2)
C12	39(3)	35(3)	26(2)	-1(2)	5(2)	4(2)
C19	61(3)	29(3)	44(3)	3(2)	31(3)	-4(2)
C2	39(3)	56(4)	36(3)	1(2)	14(2)	12(2)
C20	49(3)	40(3)	45(3)	-11(2)	32(2)	-16(2)
C5	59(3)	66(4)	38(3)	8(3)	28(3)	12(3)
C4	57(3)	74(4)	47(3)	1(3)	35(3)	3(3)
C3	49(3)	81(4)	57(4)	-6(3)	28(3)	22(3)
C24	32(3)	104(5)	53(3)	2(3)	21(2)	1(3)

Table 4 Bond Lengths for 3ba.

Atom	Atom	Length/Å	Atom	Atom	Length/Å
P1	O1	1.489(3)	C17	C16	1.469(6)
P1	C7	1.807(4)	C17	C22	1.397(5)
P1	C13	1.804(4)	C1	C6	1.379(6)
P1	C1	1.805(4)	C1	C2	1.385(5)
O2	C16	1.220(5)	C10	C9	1.373(5)
O3	C18	1.366(5)	C10	C11	1.380(5)
O3	C15	1.433(5)	C22	C21	1.378(6)

C7	C8	1.389(5)	C11	C12	1.385(6)
C7	C12	1.391(5)	C6	C5	1.383(6)
C8	C9	1.377(6)	C21	C20	1.391(6)
C14	C13	1.549(5)	C21	C24	1.517(6)
C14	C16	1.512(5)	C19	C20	1.371(6)
C14	C15	1.516(5)	C2	C3	1.393(6)
C18	C17	1.398(6)	C5	C4	1.373(6)
C18	C19	1.390(6)	C4	C3	1.372(6)

Table 5 Bond Angles for 3ba.

Atom	Atom	Atom	Angle/°	Atom	Atom	Atom	Angle/°
O1	P1	C7	111.74(17)	C6	C1	C2	119.0(4)
O1	P1	C13	113.38(18)	C2	C1	P1	118.0(3)
O1	P1	C1	112.73(18)	O2	C16	C14	122.0(4)
C13	P1	C7	105.75(18)	O2	C16	C17	122.8(4)
C13	P1	C1	106.48(18)	C17	C16	C14	115.1(4)
C1	P1	C7	106.21(19)	C9	C10	C11	119.5(4)
C18	O3	C15	114.3(3)	C21	C22	C17	122.5(4)
C8	C7	P1	117.5(3)	C10	C9	C8	120.5(4)
C8	C7	C12	118.7(4)	C10	C11	C12	120.6(4)
C12	C7	P1	123.7(3)	C1	C6	C5	120.8(4)
C9	C8	C7	120.7(4)	C22	C21	C20	117.4(4)
C16	C14	C13	113.2(3)	C22	C21	C24	120.7(4)
C16	C14	C15	110.2(3)	C20	C21	C24	121.9(4)
C15	C14	C13	110.7(3)	O3	C15	C14	112.5(3)
C14	C13	P1	111.5(3)	C11	C12	C7	120.0(4)
O3	C18	C17	122.6(4)	C20	C19	C18	119.8(4)
O3	C18	C19	117.4(4)	C1	C2	C3	120.0(4)
C19	C18	C17	120.0(4)	C19	C20	C21	122.1(4)
C18	C17	C16	120.7(4)	C4	C5	C6	120.0(5)
C22	C17	C18	118.2(4)	C3	C4	C5	120.0(5)
C22	C17	C16	121.1(4)	C4	C3	C2	120.1(5)
C6	C1	P1	123.0(3)				

Table 6 Hydrogen Atom Coordinates (Å×10^4) and Isotropic Displacement Parameters (Å^2×10^3) for 3ba.

Atom	*x*	*y*	*z*	U(eq)
H8	9408.81	5950.41	4758.08	36
H14	6654.19	4472.78	4204.32	32
H13A	6262.68	4545.69	2618.42	36
H13B	6505.76	2715.28	2773.73	36

H10	8518.3	10451.36	4325.77	41
H22	3296.45	5148.44	3327.25	43
H9	9658.32	8569.72	5132.97	39
H11	7077.24	9685.88	3167.27	45
H6	7321.24	5335.06	1756.8	52
H15A	5868.74	1406.8	3675.61	47
H15B	6992.41	1786.02	4345.06	47
H12	6801.23	7054.86	2789.12	43
H19	4717.2	1532.5	5488.36	50
H2	9556.66	2519.05	3270.03	53
H20	3108.47	2511.84	5176.52	49
H5	7902.76	5009.56	691.09	62
H4	9334.51	3496.51	922.71	66
H3	10155.56	2237.92	2202.94	72
H24A	1977.3	5316.29	4424.34	92
H24B	1509.41	3687.56	3984.39	92
H24C	1715.64	5062.44	3445.36	92
